# A multi-omics data simulator for complex disease studies and its application to evaluate multi-omics data analysis methods for disease classification

**DOI:** 10.1093/gigascience/giz045

**Published:** 2019-04-26

**Authors:** Ren-Hua Chung, Chen-Yu Kang

**Affiliations:** Division of Biostatistics and Bioinformatics, Institute of Population Health Sciences, National Health Research Institutes, No. 35, Keyan Road, Zhunan, 350, Taiwan

**Keywords:** multi-omics data, complex disease study, simulation tool

## Abstract

**Background:**

An integrative multi-omics analysis approach that combines multiple types of omics data including genomics, epigenomics, transcriptomics, proteomics, metabolomics, and microbiomics has become increasing popular for understanding the pathophysiology of complex diseases. Although many multi-omics analysis methods have been developed for complex disease studies, only a few simulation tools that simulate multiple types of omics data and model their relationships with disease status are available, and these tools have their limitations in simulating the multi-omics data.

**Results:**

We developed the multi-omics data simulator OmicsSIMLA, which simulates genomics (i.e., single-nucleotide polymorphisms [SNPs] and copy number variations), epigenomics (i.e., bisulphite sequencing), transcriptomics (i.e., RNA sequencing), and proteomics (i.e., normalized reverse phase protein array) data at the whole-genome level. Furthermore, the relationships between different types of omics data, such as methylation quantitative trait loci (SNPs influencing methylation), expression quantitative trait loci (SNPs influencing gene expression), and expression quantitative trait methylations (methylations influencing gene expression), were modeled. More importantly, the relationships between these multi-omics data and the disease status were modeled as well. We used OmicsSIMLA to simulate a multi-omics dataset for breast cancer under a hypothetical disease model and used the data to compare the performance among existing multi-omics analysis methods in terms of disease classification accuracy and runtime. We also used OmicsSIMLA to simulate a multi-omics dataset with a scale similar to an ovarian cancer multi-omics dataset. The neural network–based multi-omics analysis method ATHENA was applied to both the real and simulated data and the results were compared. Our results demonstrated that complex disease mechanisms can be simulated by OmicsSIMLA, and ATHENA showed the highest prediction accuracy when the effects of multi-omics features (e.g., SNPs, copy number variations, and gene expression levels) on the disease were strong. Furthermore, similar results can be obtained from ATHENA when analyzing the simulated and real ovarian multi-omics data.

**Conclusions:**

OmicsSIMLA will be useful to evaluate the performace of different multi-omics analysis methods. Sample sizes and power can also be calculated by OmicsSIMLA when planning a new multi-omics disease study.

## Introduction

Complex diseases such as hypertension, type 2 diabetes, and autism are caused by multiple genetic and environmental factors [1]. Genome-wide association studies have identified many genetic variants (i.e., single-nucleotide polymorphisms [SNPs]) associated with the complex diseases. However, it remains difficult to understand the roles of the associated SNPs in the molecular pathophysiology of the disease and how the SNPs interact with other SNPs in a biological network [2]. With the advance of high-throughput sequencing technology such as next-generation sequencing and massive parallel technology such as mass spectrometry, multiple types of omics data (i.e., multi-omics data) including genomics, epigenomics, transcriptomics, proteomics, metabolomics, and microbiomics are rapidly generated [3]. Because a single type of data generally cannot capture the complexity of molecular events causing the disease, an integrative approach to combining the multi-omics data would be ideal to help elucidate the pathophysiology of the disease [2].

Integrative methods to combine multi-omics data for disease studies have been developed rapidly [4–8]. They can be generally classified into 2 categories: multi-staged and meta-dimensional approaches [9]. The multi-staged approach aims to first identify relationships between the multi-omics data and then test the associations between the multi-omics data and the phenotype. For example, Jennings et al. [7] constructed a Bayesian hierarchical model consisting of 2 stages. The first stage partitioned gene expression into factors accounted for by methylation, copy number variation (CNV), and other unknown causes. These factors were subsequently used as predictors for clinical outcomes in the second-stage model. One advantage of this approach is that the causal relationships between multi-omics data can be modeled. In contrast, the meta-dimensional approach combines the multi-omics data simultaneously. Raw or the transformed data from the multi-omics data are combined into a single matrix for the analysis. This approach allows for a more flexible inference of the relationships among the multi-omics data, without the assumptions of the causal relationships between these data.

Although many multi-omics analysis methods for disease studies are available, they were generally evaluated by simulations with data generated specifically to the methods. To compare the performance among these methods, it is necessary to use the same simulated multi-omics dataset with disease status. Furthermore, when a multi-omics study is being planned, sample size estimation to ensure sufficient power also becomes important [3]. This also requires a simulation tool that simulates realistic multi-omics data structures and models the architecture of the complex disease. However, current simulation tools for disease studies have mainly focused on simulating a certain type of omics data. For example, >25 simulators are available for simulating genetic data with phenotypic trait, according to the Genetic Simulation Resources website [10]. Tools such as WGBSSuite [11] and pWGBSSimla [12] can simulate whole-genome bisulphite sequencing (WGBS) data in case-control samples. Moreover, tools such as Polyester [13] and SimSeq [14] simulate RNA sequencing (RNA-seq) data with differential gene expression between 2 groups of samples.

There are only a few available tools that can simulate multi-omics data and allow relationships among different data types to be modeled. One of them is HIBACHI [15], which provides a prototype to simulate genetic interactions under a biological architecture. The biological framework includes 6 genetic variants: 1 variant in a protein-coding gene that changes an amino acid, 2 variants that are regulatory variants in a promoter and an enhancer, 2 variants that code for transcription factors binding to the promoter and the enhancer, and 1 variant in an microRNA gene involved in post-translational regulation for the protein-coding gene. A mathematical framework is then used in HIBACHI to generate phenotypic values based on the biological framework followed by a liability threshold model to generate the disease status. Hence, HIBACHI simulates genotypes and phenotypes under the complex biological and mathematical models, but it cannot generate other types of omics data. Another tool is InterSIM [16], which simulates methylation rates and normalized gene and protein expression levels based on the ovarian cancer (OV) data from The Cancer Genome Atlas (TCGA) project [17]. The correlations within and between each data type are also modeled on the basis of the correlation structures observed in the OV data. A more recently developed tool is MOSim [18], which simulates more multi-omics data types, including RNA-seq, assay for transposase-accessible chromatin sequencing, chromatin immunoprecipitation sequencing, microRNA sequencing, and WGBS data. In contrast to InterSIM, the regulatory relationships (i.e., activation and repression effects) between the gene expression and other types of data (e.g., CpGs, transcription factors, and microRNAs) are more specifically modeled in MOSim. However, genomics data, the relationships between genomics data and other types of omics data, and the relationships between genomics data and the disease status are not simulated and modeled by InterSIM and MOSim.

Here, we developed the multi-omics data simulator OmicsSIMLA [19], which simulates genomics data including SNPs and CNVs, epigenomics data such as WGBS data, transcriptomics data (i.e., RNA-seq), and proteomics data such as the normalized reverse phase protein array (RPPA) data at a whole-genome level. Furthermore, the relationships between different types of omics data, such as methylation quantitative trait loci ([meQTLs] SNPs influencing methylation), expression quantitative trait loci ([eQTLs] SNPs influencing gene expression), and expression quantitative trait methylation ([eQTM] methylation influencing gene expression), were specifically modeled. More importantly, the relationships between these multi-omics data and disease status were modeled as well. The disease models in OmicsSIMLA are flexible so that the main effects and/or interaction effects (either risk or protective) of SNPs and CNVs on the disease can be specified. Differential methylation and differential gene and protein expression between cases and controls can also be simulated. We demonstrated the usefulness of OmicsSIMLA by simulating a multi-omics dataset for breast cancer under a hypothetical disease model and compared the performance among existing multi-omics analysis tools based on the data. We also simulated a multi-omics dataset with a scale similar to the TCGA OV data (except for the methylation data, where a smaller set of CpGs was simulated), and the effects of the multi-omics data on the phenotype were modeled based on the parameters estimated from the real OV data. We then compared the results from a multi-omics analysis method when applied to both the real and simulated OV data.

## Results

**Figure 1:**
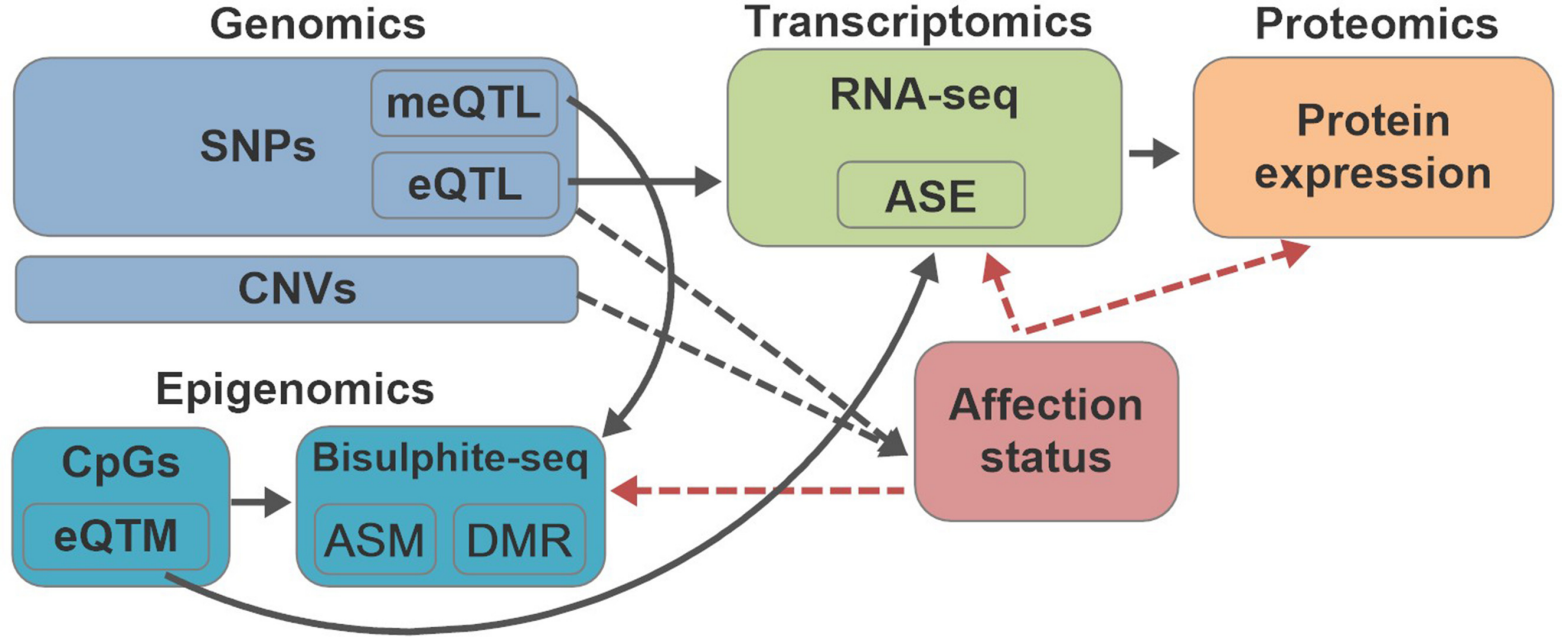
Simulation framework of OmicsSIMLA. The black solid arrows represent the relationships among different types of omics data. The black dotted arrows represent the causal effects of genomics data to the disease. The red dotted arrows represent the retrospective simulations of the methylation, gene expression, and protein expression levels conditional on the disease status.

Figure 1 shows the framework of OmicsSIMLA. The genomics data that can be simulated include SNPs and CNVs. Genotypes at SNPs in unrelated and/or family samples are simulated on the basis of the SeqSIMLA2 algorithm [20]. CNV status (i.e., a deletion, normal, 1 duplication, or 2 duplications) on a chromosome is simulated on the basis of the user-specified chromosomal regions and CNV frequencies. Affection status of each sample is determined by a logistic penetrance function conditional on the causal SNPs and CNVs, and/or the interactions among the causal SNPs. The epigenomics data are the methylated and total read counts at CpGs based on bisulphite sequencing, simulated using the pWGBSSimla algorithm incorporating methylation profiles for 29 human cell and tissue types [12]. Allele-specific methylation (ASM), in which paternal and maternal alleles have different methylation rates, and differentially methylated regions (DMRs), where the same CpGs in the region have different methylation rates among different cell types, can also be simulated. Furthermore, the transcriptomics data (i.e., RNA-seq read counts) are simulated with a parametric model assuming a negative-binomial (NB) distribution. Finally, the mass-action kinetic action model [21] is used to simulate proteomics data at a certain time point incorporating the gene expression data. Some SNPs can be specified as meQTLs and eQTLs, and some CpGs can be specified as eQTM. Allele-specific expression (ASE), which alleles in a gene have different expression levels caused by *cis*-eQTL, can also be simulated. The differential methylation, gene expression, and protein expression levels between cases and controls are simulated conditional on the affection status.

To simulate the SNP data, external reference sequences (i.e., haplotypes) generated by an external sequence generator, such as COSI [22] or HAPGEN2 [23], are required. Using HAPGEN2, we compiled several reference sequence files for the African, Asian, and European populations similar to the linkage disequilibrium structures and allele frequencies of the variants on chromosome 1 in the 1000 Genomes Project data [24]. For the CNV simulations, a CNV information file, which contains the CNV types (i.e., deletion, normal, or duplication), CNV frequencies, and odds ratios (ORs) of the CNVs for the disease, is required. We compiled CNV profiles based on the frequencies of 2,884 focal CNVs observed in the TCGA data for 33 cancers. The optional files for simulating the genomics data include a recombination file, which specifies the recombination rates among SNPs; a pedigree file, which specifies the pedigree structures if family data are simulated; and a proband file, which specifies the affection status of the family members. For the WGBS simulations, we compiled profiles of 29 human cell and tissue types such as liver, kidney, and colon [12]. We also compiled profiles of normal and tumor tissue types for 31 cancers to simulate the RNA-seq data. Finally, we compiled profiles of tumor tissues for 26 cancers to simulate the protein expression data. Therefore, the user can easily specify the tissue types and the numbers of samples to simulate the CNV, WGBS, RNA-seq, and protein expression data. The formats of the profiles are clearly described in the OmicsSIMLA user manual so that the user can alternatively compile profiles based on his/her own data. Further details on how the profiles are compiled and how OmicsSIMLA generates data based on the profiles are described in the Methods section.

Several options are available in OmicsSIMLA to flexibly model the relationships between the multi-omics data and the disease. For example, the ORs for the main effects and pairwise interaction effects of selected SNPs can be specified. An additive, dominant, or recessive model can be assumed for the main effects, and several interaction models can be specified, as described in the Methods section. The user can also specify the proportions of methylated (i.e., methylation rates > 70%), unmethylated (i.e., methylation rates < 30%), and partially methylated (i.e., methylation rates between 30% and 70%) CpGs that have different methylation rates between cases and controls, and the difference in the methylation rates can also be specified. The fold changes of the gene expression levels for the differentially expressed genes between cases and controls can also be specified. Several options are also available to model the relationships between the multi-omics data. For example, the fold changes of the methylation rates influenced by meQTLs can be specified. Similarly, fold changes of the gene expression levels influenced by eQTLs and eQTMs can be specified. A user-friendly web interface is provided [25] to conveniently specify the abovementioned parameters. All of the input files and parameters that are required and optional to OmicsSIMLA are clearly described on the web interface.

**Figure 2:**
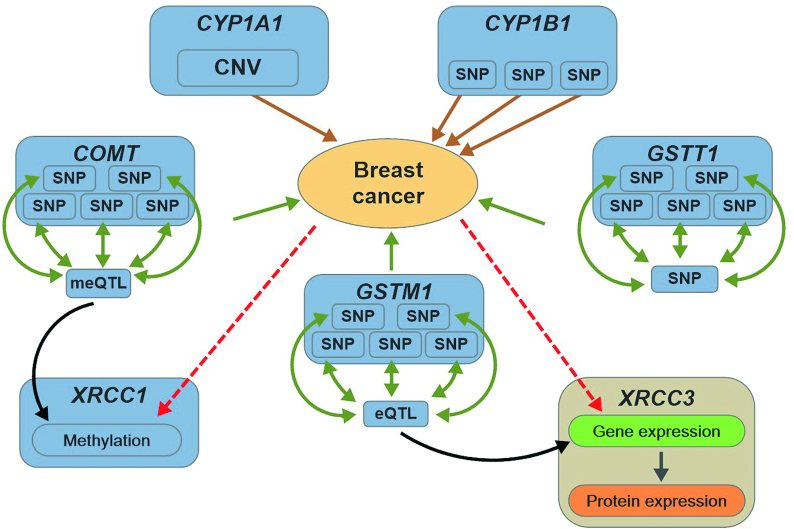
Hypothetical pathways involved in breast cancer. The brown solid arrows represent the main effects of SNPs and CNVs on the disease, while the green solid arrows represent the interaction effects of SNPs on the disease. The black solid arrows represent the regulatory effects of the meQTL and eQTL on methylation and gene expression, respectively. The red dotted arrows represent the retrospective simulations of the methylation, gene expression, and protein expression levels conditional on the disease status.

Using OmicsSIMLA, we simulated a multi-omics dataset based on hypothetical pathways for breast cancer as described in Ritchie et al. [9] and illustrated in Figure 2. The data included a deletion with a protective effect in the *CYP1A1* gene, 3 common SNPs with risk effects in the *CYP1B1* gene, 5 rare SNPs in the *COMT* gene, which had interaction effects with an meQTL for the *XRCC1* gene, and 5 rare SNPs in the *GSTM1* gene, which also had interaction effects with an eQTL affecting the gene and protein expression of the *XRCC3* gene. Finally, 5 rare SNPs in the *GSTT1* gene also had interaction effects with an SNP in a regulatory region. A total of 2,022 SNPs in the 4 genes (i.e., *CYP1B1*, *COMT*, *GSTM1*, and *GSTT1*) and a regulatory region consisting of the meQTL, eQTL, and the SNP interacting with *GSTT1*, 1 CNV in *CYP1A1*, 688 CpGs in *XRCC1*, and gene and protein expression levels for 100 genes (including the expression for *XRCC3* and 99 other hypothetical genes in the pathways) were simulated. More details about the simulations can be found in the Methods section.

Based on the simulated datasets, we compared the performance of methods from the 2 categories of multi-omics analysis methods (i.e., multi-staged and meta-dimensional approaches) for disease prediction by measuring the area under the curve (AUC). The SNPs, CNV, methylation levels at CpGs, and gene and protein expression levels were used as the features for the prediction. The disease status served as the label for classification, and the prediction accuracy was measured based on the numbers of cases and controls that were correctly predicted. For the multi-staged method, we implemented the 3-stage method [26]. Briefly, significant SNPs and CNV associated with the disease (i.e., association *P*-values <0.05) were first selected. The significant SNPs and CNV were then tested for associations with each feature in the methylation and gene and protein expression data, and the significant features were selected. Finally, a logistic regression prediction model was constructed based on these significant features. Further details on how the 3-stage method was implemented are provided in the Supplementary methods. The meta-dimensional methods we used included the random forest–based method (RFomics), a graph-based integration method (CANetwork) [5], and a model-based integration method (ATHENA) [4]. The RFomics combines the preprocessed multi-omics data in a single matrix for constructing the prediction model. As described in the Supplementary methods, a gene-based risk score is calculated based on SNPs for each gene. Then the risk scores and other multi-omics data are normalized so that they can be evaluated on the same scale by the random forest (RF) algorithm. In contrast, CANetwork calculates a graph matrix to measure the distance between samples using the composite association network algorithm [27], and the prediction model is created on the basis of the distance matrix using the graph-based semi-supervised learning algorithm [28]. Finally, ATHENA uses grammatical evolution neural networks (GENNs), which optimize artificial neural networks based on genetic programming, to construct a meta-dimensional model from multi-omics data for prediction. The parameters for RFomics and ATHENA used in our simulations are shown in Supplementary Table S1.

Table 1 shows the AUC for the 4 methods under 3 scenarios. Scenario 1 had 500 cases and 500 controls in the training set, and 100 cases and 100 controls in the validation set. Scenario 2 had the same sample sizes as those in Scenario 1, but the multi-omics data had weaker effects on the disease compared with Scenario 1. The effects of the multi-omics data were the same in Scenario 3 as those in Scenario 1, but Scenario 3 had larger sample size (i.e., 1,500 cases and 1,500 controls in the training data and 500 cases and 500 controls in the validation data). More details of the 3 scenarios are provided in the Methods section. Prediction models for the 4 methods were created on the basis of the training data, and their prediction accuracies were evaluated by the validation data. In Scenarios 1 and 3, ATHENA had a significantly higher AUC than the other 3 methods. The 3-stage method had an AUC similar to RFomics, and CANetwork had the lowest AUC. In Scenario 2, where the effects on the disease were weaker, the 4 methods had similar AUCs. Table 2 shows the runtime for the 4 methods. In Scenario 1, RFomics and CANetwork had similar performance, whereas ATHENA had >500 times the runtime of RFomics and CANetwork. In Scenario 3, CANetwork was the most efficient method followed by the RFomics and the 3-stage method, and ATHENA had a significantly longer runtime than the other 3 methods.

**Table 1:** Area under the curve (AUC) for the 3-stage, RFomics, CANetwork, and ATHENA methods under 3 scenarios

Mean AUC (SE)^1^				
Scenario	3-Stage	RFomics	CANetwork	ATHENA
1	0.821 (0.028)	0.825 (0.028)	0.626 (0.037)	0.964 (0.017)
2	0.501 (0.042)	0.511 (0.038)	0.529 (0.027)	0.509 (0.041)
3	0.825 (0.013)	0.845 (0.013)	0.679 (0.019)	0.969 (0.005)

1 Estimated on the basis of 100 batches.

**Table 2:** Runtime for the 3-stage, RFomics, CANetwork, and ATHENA methods under Scenarios 1 and 3

Mean runtime (seconds)				
Scenario	3-stage	RFomics	CANetwork	ATHENA
1	72.90	37.78	40.54	39,533.91
3	230.32	143.91	94.16	113,872.50

1 Estimated on the basis of 100 batches..

**Figure 3:**
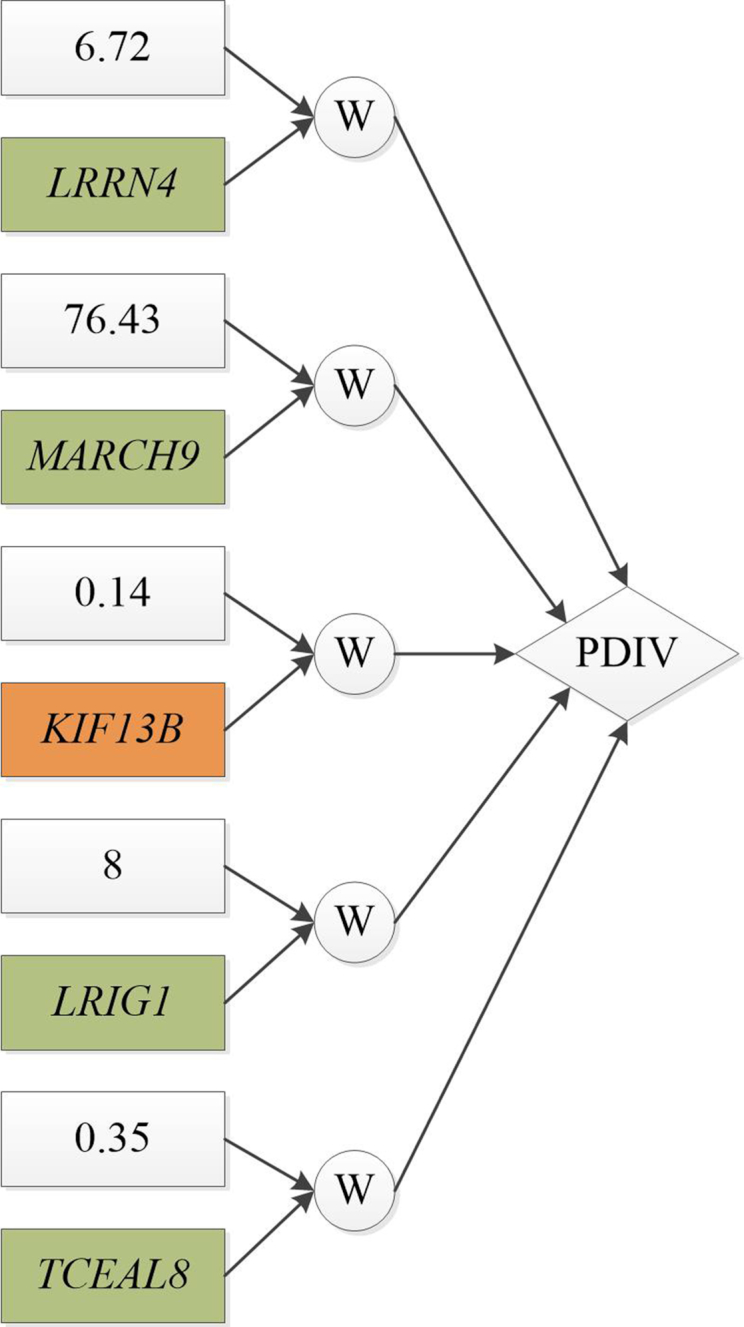
The GENN model with the best AUC for the TCGA OV dataset. Green and orange boxes represent gene expression and methylation features, respectively. W is the weight associated with the feature, and PDIV is a division activation node.

**Figure 4:**
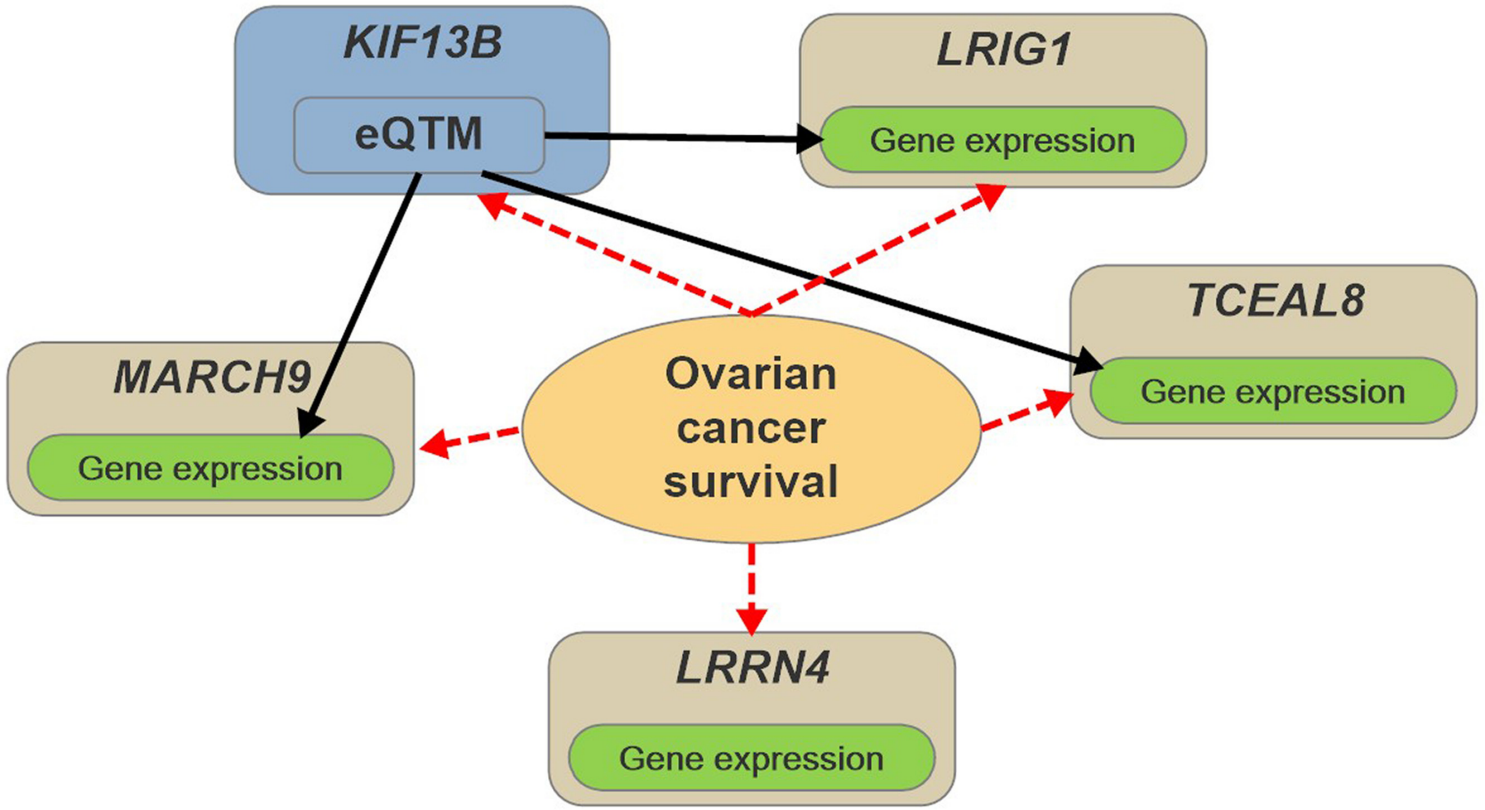
Hypothetical model for the survival time (short-term and long-term) of OV. The black solid arrows represent the regulatory effects of the eQTM on gene expression. The red dotted arrows represent the retrospective simulations of the methylation and gene expression levels conditional on the survival status.

**Figure 5:**
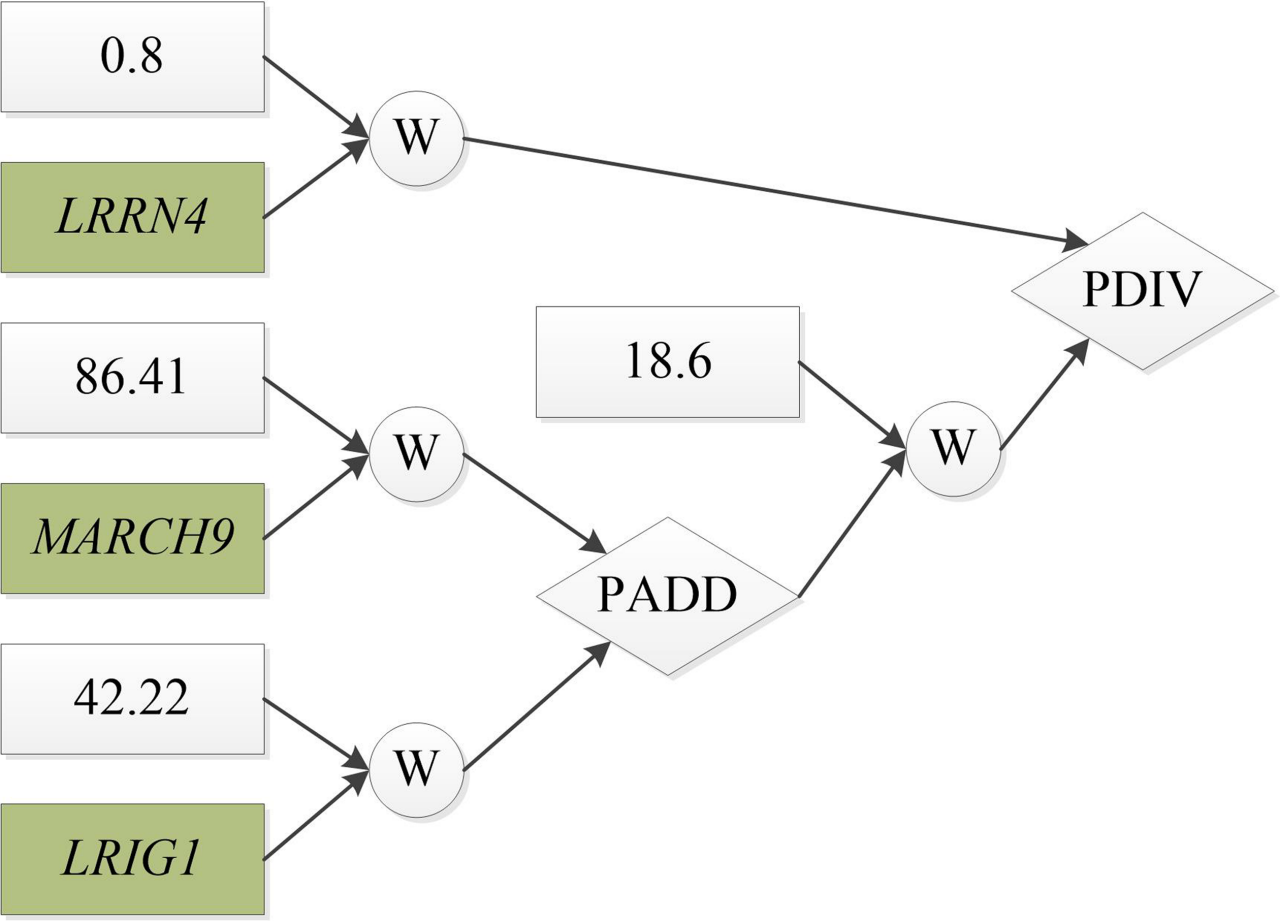
The GENN model constructed by ATHENA using the simulated OV data, which has a similar structure to the GENN model in Figure 3. W is the weight associated with the feature, and PADD and PDIV are an addition and division activation node, respectively.

We also used OmicsSIMLA to simulate a multi-omics dataset based on the TCGA OV data. The TCGA OV data included focal CNV, methylation, RNA-seq, and RPPA data in 66 individuals with short-term survival (i.e., survival time of $\leq$3 years) and 107 individuals with long-term survival (i.e., survival time of >3 years). Further details of the OV data are provided in the Methods section. Our simulation results showed that ATHENA had a higher AUC than the other 3 multi-omics analysis methods; hence, we applied ATHENA to the TCGA OV data several times with different random seeds to identify a GENN model that can classify the short-term and long-term survivals with the highest AUC. As shown in Figure   3, the best GENN model constructed by ATHENA (having an AUC of 0.826) comprised 5 features, including methylation of a CpG at the *KIF13B* gene and gene expression levels at the *LRRN4*, *MARCH9*, *LRIG1*, and *TCEAL8* genes. By examining the correlation structures in the 5 features, we hypothesized that the correlations among the gene expression levels of *MARCH9*, *LRIG1*, and *TCEAL8* were caused by the CpG at *KIF13B* (i.e., an eQTM), and the gene expression of *LRRN4* exhibited an independent effect on the survival time. We then used OmicsSIMLA to simulate similar numbers of focal CNVs and genes with RNA-seq and RPPA data as those in the OV data. We also simulated methylation levels at CpGs on chromosome 1. A CpG with differential methylation was specified as an eQTM, which affected the gene expression of 3 genes. The 3 genes were also differentially expressed. Furthermore, an independent gene was specified to be differentially expressed as well. The model for the survival time is illustrated in Figure 4. A total of 50 replicates of 500 cases (i.e., the short-term survival group) and 500 controls (i.e., the long-term survival group) were simulated. Each replicate included 2,884 focal CNVs, 2,753 CpGs, gene expression levels for 12,004 genes, and protein expression levels for 200 genes. Finally, ATHENA was applied to the 50 replicates. We found that up to 3 of the 5 causal features can be selected in the same model by ATHENA in the 50 replicates. The mean AUC calculated from 5-fold cross-validation in ATHENA over the 50 replicates was 0.757, which was comparable to the AUC of 0.826 calculated from the real data. Figure 5 shows a GENN model from 1 of the 50 replicates that connected the features with a PDIV node similar to the one in Figure 3. The results demonstrated that OmicsSIMLA can simulate multi-omics data with a scale similar to the real dataset and incorporate a model with similar effects on the survival time.

## Discussion

We have developed OmicsSIMLA, which simulates multi-omics data (i.e., genomics, epigenomics, transcriptomics, and proteomics data) with disease status. OmicsSIMLA simulates multiple types of omics data while the relationships between different types of omics data and the relationships between the omics data and the disease are modeled. As the development of integrative methods for analyzing multi-omics data has attracted substantial interest from researchers, OmicsSIMLA will be useful to simulate benchmark datasets for comparisons of these methods. Furthermore, as more and more disease studies take advantage of multi-omics data, OmicsSIMLA will also be useful for power calculations and sample size estimations when planning a new study.

Performing simulation studies using OmicsSIMLA has several advantages and disadvantages as compared with using HIBACHI, InterSIM, and MOSim. For example, the mathematical framework in HIBACHI for the 6 genetic variants and an environmental factor allows the user to generate complicated genetic models, including high-order gene-gene interactions, but only pairwise gene-gene interactions are modeled in OmicsSIMLA. However, an advantage of OmicsSIMLA over HIBACHI is that not only genomics data and disease status but also other types of omics data can be simulated. On the other hand, InterSIM models the correlations within and among the methylation, gene expression, and protein expression data based on correlation structures observed in the TCGA OV data. OmicsSIMLA can model the correlations of methylation levels at local CpGs [12]. The gene and protein expression levels for different genes are generally independently simulated in OmicsSIMLA. The correlations of gene and protein expression levels between different genes can be modeled by a common regulatory variant, such as an eQTL or an eQTM in OmicsSIMLA. By contrast, MOSim is more flexible in simulating different experimental designs, such as different numbers of experimental groups, data at different time points, and different numbers of replicates under an experimental condition. However, InterSIM and MOSim do not simulate genomics data; hence, eQTLs and meQTLs cannot be generated. From the above discussion, we emphasized that the 4 simulators have their own capabilities, advantages, and disadvantages; hence, the choice of a proper simulator must be based on the purpose of the study performed.

We used OmicsSIMLA to simulate a multi-omics dataset for breast cancer based on hypothetical pathways. Four analysis tools were compared using the dataset. The results showed that the neural network–based method ATHENA achieved the highest AUC when the effects of the multi-omics features on the disease were strong. However, the AUCs of the 4 methods were similar when the effects were modest. Furthermore, RFomics and CANetwork had comparable runtimes, and ATHENA was the most computationally expensive approach. In practice, ATHENA is the ideal tool to perform multi-omics data analysis because of its high AUC if the execution time is acceptable.

We also used OmicsSIMLA to simulate a multi-omics dataset with a scale similar to the TCGA OV data. The effects of the multi-omics features on the phenotype were estimated and modeled on the basis of the OV data. As shown in the simulations of the breast cancer pathways and the simulations based on the OV data, 3 main components are generally required to perform the simulations in OmicsSIMLA. These include a biological model (i.e., the disease model), reference sequences and profiles, and parameter values to model the effects of the multi-omics features on the disease and to model the relationships between multi-omics data. The biological model is usually hypothesized on the basis of the literature results and observations in real data. Profiles such as the CNV, methylation, gene expression, and protein expression profiles are then required to simulate the multi-omics data. Because we have compiled profiles for many tissue types, the user can conveniently choose the profiles to perform the simulations. Note that the profiles were compiled using the tumor tissue data from the TCGA project, except for the methylation profiles and some of the gene expression profiles. If multi-omics data for other tissue types are available, the user can also compile the profiles based on the data following the instructions in the user manual. To simulate genomics data, we compiled several sets of reference sequences for the African, Asian, and European populations based on allele frequencies and linkage disequilibrium structures in human sequences. Finally, several parameters are needed in OmicsSIMLA to specify the disease model and to model the relationships among the multi-omics data; recommended values of some of these parameters are provided in the user manual. For example, the ORs of common SNPs (i.e., minor allele frequencies [MAFs] ≥ 5%) for complex diseases were generally observed to be between 0.5 and 2 according to the GWAS (genome-wide association studies) catalog [29], and the ORs of rare SNPs (MAFs < 5%) can be a function of the MAFs [30]. Note that performing the simulations using the pre-compiled profiles, reference sequences, and recommended parameter values is simplified, but the user can still opt to customize the profiles and reference sequences with specific parameter values to flexibly perform the simulations.

Currently, OmicsSIMLA focuses on simulating the dichotomous trait (i.e., affection status). Because studies for quantitative traits are also important, it is our future work to extend OmicsSIMLA to simulate quantitative traits based on the classic quantitative genetics model [31]. Furthermore, environmental factors and the interactions between genes and environments can also play important roles in complex disease etiology. Therefore, simulating exposome data such as climate and air quality data and modeling their interactions with genes are also important in the future extensions of OmicsSIMLA.

## Conclusions

In conclusion, we developed a useful multi-omics data simulator, OmicsSIMLA, for complex disease studies. Benchmark datasets can be simulated by OmicsSIMLA for evaluating different multi-omics data analysis methods for disease studies. OmicsSIMLA can also be used to estimate sample sizes and statistical power when designing a new multi-omics disease study. OmicsSIMLA is freely available [19].

## Methods

### Simulation of DNA sequences

The SeqSIMLA2 package [20] is integrated in OmicsSIMLA to generate DNA sequences in unrelated/related individuals. Similar to SeqSIMLA2, OmicsSIMLA expects a set of external reference sequences (i.e., haplotypes) generated by an external sequence generator such as COSI [22] or HAPGEN2 [23] that has been widely adopted in genetics studies. Generally, a set of ≥10,000 reference sequences are expected. Optional files consisting of recombination rate information and pedigree structures are also accepted. A gene-dropping algorithm assuming random mating with crossovers is performed based on the reference sequences, recombination rates, and pedigree structures to generate haplotypes in each individual.

### Simulation of CNVs

For the simulation of CNVs, we considered 4 CNV states including deletion (D), normal (N), 1 duplication (U), and 2 duplications (UU) on a chromosome. Therefore, there are 10 types of CNV states on the 2 chromosomes in an individual, as shown in Supplementary Table S2, and the total copy numbers on the 2 chromosomes range from 0 to 6. During meiosis, we use the single-copy crossover model, assuming all crossovers occurred between CNVs [32]. We compiled profiles of CNV frequencies of D and U for 2,884 focal CNVs observed in the TCGA data for 33 cancers. Further details on how the CNV profiles were generated are provided in the Supplementary methods. Alternatively, the user can provide frequencies and ranges of the 4 CNV states for different CNV regions.

### Simulation of affection status

Genetic variants, including SNPs and CNVs, are used to determine the affection status of an individual based on a logistic penetrance function as follows:

\begin{eqnarray*}
{\rm{logit}}(P(\mathrm{affected})) &=& \mathop \beta \nolimits_0 + \sum\limits_{i \in \Omega } {\mathop \beta \nolimits_{{C_{i1}}} {C_{i1}}} + \sum\limits_{i \in \Omega } {\mathop \beta \nolimits_{{C_{i2}}} {C_{i2}}} \nonumber \\
&& + \sum\limits_{j \in \Psi } {\mathop \beta \nolimits_{{G_j}} {G_j}} + \sum\limits_{m,n \in \Upsilon } {\mathop \beta \nolimits_{mn} {G_{mn}}},
\end{eqnarray*}

where *P*(affected) is the probability of being affected; $\mathop \beta \nolimits_0 $ determines the baseline prevalence; $\Omega $, $\Psi $, and $\Upsilon $ are sets of causal CNVs, SNPs with main effects, and SNPs with interaction effects, respectively, specified by the user; *C_i1_* and *C_i2_* are the CNV states for the first and second haplotypes at CNV *i*, respectively; *G_j_* is the genotype coding at SNP *j*; and *G_mn_* is the genotype coding at SNPs *m* and *n*. *C_i1_ and C_i2_* have values of –1, 0, 1, and 2 for CNV states *D*, *N*, *U*, and *UU*, respectively, where *N* is the baseline state. The coding of *G_j_* is based on a dominant, additive, or recessive model, and the coding of *G_mn_* is based on several interaction models. If SNP *j* is in a CNV region, allelic CNV [33] is considered in the coding of *G_j_*. More details of the coding of *G_j_* and *G_mn_* are provided in the Supplementary methods. The parameters $\mathop \beta \nolimits_C $ and $\mathop \beta \nolimits_G $ are the effect sizes of the main effects for CNVs and SNPs, respectively, and $\mathop \beta \nolimits_{mn} $ determines the effect size of the interaction effect between SNPs *m* and *n*. These parameters are specified by the user.

### Simulation of DNA methylation data

The pWGBSSimla package [12] is integrated into OmicsSIMLA to generate the WGBS data. The pWGBSSimla algorithm simulates data using methylation profiles generated based on 41 WGBS datasets for 29 human cell and tissue types. The profiles contain the information for each CpG, such as its distance to the next site, methylation rate, methylation status (i.e., methylated, unmethylated, and fuzzily methylated), and read counts for each type of methylation status. CpGs and the distances between the CpGs are first determined on the basis of the profiles, and then the total read count and methylated read count are simulated for each CpG. Methylation level at a CpG influenced by an meQTL is simulated on the basis of a genotype-specific methylation probability, which is the methylation rate of the CpG in the profiles multiplied by a user-specified ratio. Furthermore, ASMs are simulated on the basis of father- and mother-specific methylation rates for paternal and maternal alleles, respectively. Finally, a DMR is generated by simulating the same genomic region using profiles for different cell or tissue types. OmicsSIMLA currently simulates methylation data for CpGs on the same chromosome per run. Multiple runs of OmicsSIMLA can be executed to simulate CpGs on different chromosomes. More details of the pWGBSSimla algorithm can be found in Chung and Kang [12].

### Simulation of RNA-seq data

We implemented a parametric simulation procedure for simulating the RNA-seq data similar to that described in Benidt and Nettleton [14]. An NB distribution with mean ${\mu _{ij}}$ and dispersion parameter ${\omega _i}$ is used to simulate the read count for gene *i* in individual *j*. The mean is calculated as ${\mu _{ij}} = {\lambda _i}{c_j}$, where ${\lambda _i}$ is the common mean for gene *i* and ${c_j}$ is the individual-specific normalization factor for individual *j*. The individual-specific normalization factor is used to model systematic variations among individuals due to technical variation [34]. We compiled RNA-seq profiles for normal and tumor tissues of 31 cancers based on the TCGA whole-genome RNA-seq data. For each tissue type, the profiles comprised a vector ***c*** of individual-specific normalization factors calculated based on the TCGA samples and vectors $\boldsymbol{\lambda }$ and $\boldsymbol{\omega }$ for genes across the genome. Further details on how the RNA-seq profiles were generated are provided in the Supplementary methods. Note that when there are no technical variations to simulate, the user can replace the vector ***c*** with a vector of ***1***, where the length of the vector is the number of samples to be simulated. The parameters ${\lambda _i}$ and ${\omega _i}$ are then randomly sampled with replacement from $\boldsymbol{\lambda }$ and $\boldsymbol{ \omega }$. If more samples than those in the TCGA data are simulated, we use the smoothed bootstrap procedure [35] to calculate $c_j^*$ for individual *j*, and ${\mu _{ij}}$ is calculated as ${\lambda _i}c_j^*$. More details of the calculation of $c_j^*$ are also provided in the Supplementary methods. The user can specify *n* differentially expressed (DE) genes between cases and controls and their fold changes, and the read count for DE gene *i* in individual *j* is simulated on the basis of an NB distribution with mean ${f_i}{\mu _{ij}}$ and dispersion parameter ${\omega _i}$, where *f_i_* is the fold change for gene *i*.

#### Simulation of eQTL and allele-specific reads

We followed the procedure in the simulation study by Sun [36] to simulate eQTL and read counts for ASE. For eQTL *l* with a user-specified fold change *h_l_*, the means for the 3 genotypes *AA*, *Aa*, and *aa* at the eQTL are ${\mu _{ij}}$, ${h_l}{\mu _{ij}}$, and $(2{h_l} - 1){\mu _{ij}}$, respectively, and the dispersion parameter is ${\omega _i}$ in the NB distribution for gene *i* influenced by the eQTL. ASE for a gene caused by a *cis*-eQTL is simulated by assuming that reads were mapped to heterozygous SNPs (i.e., allele-specific reads) in the gene. A *cis*-eQTL refers to the eQTL being located in the *cis*-regulatory elements of the gene. Because the alleles at the *cis*-eQTL can be in the same haplotype as the alleles of the gene, ASE can be observed using the allele-specific reads of the gene. Furthermore, only heterozygous SNPs can be tested for *cis*-eQTL with the allele-specific reads. Therefore, we simulate allele-specific reads for heterozygous eQTLs. Assuming *t_ij_* is the total read count for gene *i* in individual *j*, the total number of allele-specific reads is calculated as 0.005*t_ij_*, where 0.005 was estimated from real data by Sun [36]. Furthermore, also suggested by Sun [36], the number of allele-specific reads for a haplotype is simulated using a β-binomial distribution with a mean determined by the effect size of the *cis*-eQTL and an overdispersion parameter of 0.1. The effect size is defined as log_2_(expression of the alternative allele at the eQTL/expression of the reference allele at the eQTL) [37] for a heterozygous *cis*-eQTL and is set to 0 for a homozygous *cis*-eQTL.

#### Simulation of eQTM

We used linear regression to model the relationship between gene expression and methylation:

$\mu _i^{\prime} = E({y_{ij}}) = {\alpha _i} + {\beta _i}{x_{ij}}$, where *y_ij_* and *x_ij_* are the RNA-seq read count and the proportion of methylated reads, respectively, for gene *i* influenced by methylation in individual *j*. Assuming that the NB parameters for gene *i* are ${\mu _i}$ and ${\phi _i}$, the parameter ${\alpha _i}$ is specified as ${\mu _i}$, and ${\beta _i}$ is assumed to follow a normal distribution with a mean and a standard deviation specified by the user. Then the gene expression of gene *i* is simulated by an NB distribution with parameters of $\mu _i^{\prime}$ and ${\phi _i}$.

### Protein expression simulation

We assumed that the protein expression level for protein *k* at a time point *t* in sample *j* follows a normal distribution with a mean ${\eta _{kjt}}$ and a standard deviation ${\tau _k}$ after normalization. We used the mass-action kinetic action model [21] to simulate protein expression at a certain time point. The mean ${\eta _{kj,t + 1}}$ for the protein expression at time *t*+1 was determined as follows:

\begin{equation*}
{\eta _{kj,t + 1}} = {\eta _{kjt}} + ({x_{kjt}}\kappa _{jt}^s - {\eta _{kjt}}\kappa _{jt}^d),
\end{equation*}

where ${x_{kjt}}$ is the normalized gene expression for the gene encoding protein *k*, and $\kappa _{jt}^s$ and $\kappa _{jt}^d$ are the protein synthesis and degradation rates, respectively, in individual *j* at time *t*. The normalized gene expression ${x_{kjt}}$ is calculated using the median absolute deviation scale normalization [38] based on the RNA-seq data simulated from the previous section. Similar to the simulation study in Teo et al. [21], $\kappa _{jt}^d$ is fixed to be 1, and $\kappa _{jt}^s$ with a default value of 1 can be changed by the user. We compiled protein expression profiles consisting of a vector of standard deviations $\tau $ for 26 cancers from the TCGA project. The standard deviations were estimated from the level 4 protein expression data of each tissue type with >50 patients. The level 4 data consisted of protein expression data that have been normalized across the samples as well as across the proteins, and a replication-based method was used to account for differences in protein expression among different batches. More details about the generation of the profiles are provided in the Supplementary methods. The parameter ${\tau _j}$ is then randomly sampled with replacement from $\tau $.

### Simulation studies based on the hypothetical breast cancer disease model

We used OmicsSIMLA to evaluate the performance of the 3-staged method, RFomics, CANetwork, and ATHENA. A hypothetical disease model for breast cancer involving multi-omics data [9] was simulated, as shown in Figure 2. To be more specific, a deletion with a frequency of 20%, which had a protective effect with an OR of 0.67, in the *CYP1A1* gene and 3 common variants, which had main effects (ORs = 1.5) with MAFs > 10%, in the *CYP1B1* gene were simulated. We also simulated 5 rare variants with MAFs < 3% in the *COMT* gene, which had interaction effects (ORs = 5) with an meQTL for the *XRCC1* gene. The CpG in *XRCC1* influenced by the meQTL caused a difference in methylation rates of 10% between cases and controls. Furthermore, we simulated 5 rare variants in the *GSTM1* gene, which had interaction effects (ORs = 5) with a *cis*-eQTL for the *XRCC3* gene, and 5 rare variants in the *GSTT1* gene, which had interaction effects (ORs = 5) with an SNP located in the same region as that of the meQTL and eQTL. The eQTL caused a fold change of 1.5 in the *XRCC3* gene expression compared with the reference genotype, and a fold change of 1.5 was simulated for the differential gene expression of *XRCC3* between cases and controls. In summary, the total variables consisted of 200, 687, 264, and 176 SNPs in the *CYP1B1*, *COMT*, *GSTM1*, and *GSTT1* genes, respectively, and 695 SNPs harboring the meQTL, eQTL, and the SNP interacting with GSTT1 in the regulatory region, a variable for CNV status in *CYP1A1*, methylation levels at 688 CpGs in *XRCC1*, and gene and protein expression levels for 100 genes and their encoded proteins. More details for generating the reference sequences in the genes and the simulations for each omics data type are provided in the Supplementary methods.

We simulated a training dataset consisting of 500 cases and 500 controls as well as a validation dataset consisting of 100 cases and 100 controls. The training dataset was used by the 3-staged method, RFomics, CANetwork, or ATHENA, to construct a prediction model. The validation dataset was then used to calculate the AUC based on the prediction model. Note that a 5-fold cross-validation was performed in ATHENA, and a best model based on the testing dataset (i.e., 1 of the 5 random 20% of the training dataset) was created for each cross-validation. The model with the highest AUC based on the testing dataset was selected and applied to the validation dataset. This simulation scenario was referred to as Scenario 1. We also simulated a scenario with weaker genetic effects (Scenario 2) and a scenario with larger sample size (Scenario 3). More details about Scenarios 2 and 3 are provided in the Supplementary methods. For each scenario, 100 batches of training and validation datasets were simulated, and the AUC for each algorithm was averaged over the 100 batches.

### Simulation studies based on the TCGA OV data

We also used OmicsSIMLA to simulate a multi-omics dataset based on the OV data from the TCGA project. The data were downloaded using RTCGAToolbox [39], an R package that allows the retrieval of the TCGA pre-processed data from the Firehose pipeline [40]. A total of 173 tumor samples with clinical (i.e., the survival time), CNV, methylation, RNA-seq, and RPPA data available were extracted. In accordance with the definition used by Kim et al. [41], patients with a survival time shorter than 3 years were referred to as short-term survivors, whereas patients with a survival time longer than 3 years were referred to as long-term survivors. The CNV data consisted of the discrete CNV statuses at focal CNVs based on GISTIC2 [42] calls in 2,884 genes. The methylation data had methylation rates at 25,794 CpG sites in 13,157 genes. The RNA-seq data comprised the RNA-Seq by Expectation Maximization (RSEM) [43] counts of 17,946 genes, and the RPPA data comprised the normalized protein expression data of 204 genes.

Each of the features in each type of omics data was first tested for association with the survival time (i.e., short-term and long-term survival) by fitting a logistic regression model. For each type of omics data, the 50 most significant features sorted via the association *P*-values were used in ATHENA. The parameter values shown in Supplementary Table S1 were specified in ATHENA. We then examined the pairwise correlations among the 5 features identified by the best model shown in Figure 3 from ATHENA. We found that the gene expression of LRRN4 had low Pearson correlation coefficients with the other 4 features. However, there were significant correlations between the methylation level of KIF13B and the gene expression levels of MARCH9 and TCEAL8. There were also significant correlations among the gene expression levels of MARCH9, LRIG1, and TCEAL8. The Pearson correlation coefficients among the 5 features are presented in Supplementary Table S3. To establish the correlations between methylation and gene expression features as well as the correlations among gene expression features using OmicsSIMLA, we hypothesized that the methylation of KIF13B was an eQTM, which affected the gene expression levels of MARCH9, LRIG1, and TCEAL8. We selected a CpG with a similar methylation rate (i.e., 2%) to that in KIF13B. The CpG was assumed to have a different methylation rate (i.e., 2.3%) in the simulated long-term survival group compared to the 2% rate in the short-term survival group. The rates were similar to those observed in the OV data. Furthermore, the parameter *β*, which was used to model the relationship between the methylation of the eQTM and gene expression levels of 3 genes, was assumed to have a mean of 50 and a standard deviation of 20. The parameters were estimated from the OV data. The fold changes of the 3 genes influenced by the eQTM were all specified to be 0.6 in the short-term survival group relative to the long-term survival group. Finally, the fold change of the independent gene expression was specified to be 2 in the short-term survival group relative to the long-term survival group. The fold changes were also similar to those observed in the OV data. The simulations were performed based on the CNV, methylation, gene expression, and protein expression profiles compiled according to the TCGA OV data. Further details on how the profiles were generated are provided in the Supplementary methods. A total of 50 batches of data were simulated, with each having 2,884 focal CNVs, 2,753 CpGs on chromosome 1, gene expression levels for 12,004 genes, and protein expression levels for 200 genes in 500 cases (i.e., the short-term survival group) and 500 controls (i.e., the long-term survival group).

## Availability of supporting source code and requirements

Project name: OmicsSIMLA

Project home page: https://omicssimla.sourceforge.io

Operating system: Linux

Programming language: C++

Other requirements: C++11 compiler and Eigen and boost libraries if directly compiling the source code

License: GPL-3.0

RRID:SCR_017011

## Availability of supporting data and materials

The simulated datasets supporting the conclusions of this article are available from the OmicsSIMLA website [19]. Snapshots of the code and other supporting data are available in the *GigaScience* repository, GigaDB [44].

## Additional files

Supplementary Methods:

The implementation of the three-stage method

Coding of *G_j_*and *G_mn_*

Generation of the CNV profiles

Generation of the RNA-seq profiles

Calculating the individual-specific normalization factor

Generation of the RPPA profiles

Calculating the risk score of a gene in RFomics

Simulation studies for the hypothetical breast cancer pathways

Generation of the TCGA ovarian cancer profiles

Supplementary Tables:

Table S1. Parameters for ATHENA and random forest

Table S2. CNV states considered in OmicsSIMLA

Table S3. Pearson correlation coefficients among the features selected by ATHENA

## Abbreviations

ASM: allele-specific methylation; AUC: area under the curve; CNV: copy number variation; DE: differentially expressed; DMR: differentially methylated region; eQTL: expression quantitative trait locus; eQTM: expression quantitative trait methylation; GENN: grammatical evolution neural network; MAF: minor allele frequency; meQTL: methylation quantitative trait locus; NB: negative-binomial; OR: odds ratio; OV: ovarian cancer; RF: random forest; RNA-seq: RNA sequencing; RPPA: reverse phase protein array; SNP: single-nucleotide polymorphism; TCGA: The Cancer Genome Atlas; WGBS: whole-genome bisulphite sequencing.

## Competing interests

The authors declare that they have no competing interests.

## Funding

This work has been supported by a grant from the Ministry of Science and Technology (MOST 106-2221-E-400-005-MY3) of Taiwan.

## Authors’ contributions

R.H.C. and C.Y.K. both designed the framework of the simulation tool and implemented the software. R.H.C. designed the simulation study and C.Y.K. performed the simulation analysis. Both authors read and approved the final manuscript.

## Supplementary Material

GIGA-D-18-00397_Original_Submission.pdf

GIGA-D-18-00397_Revision_1.pdf

GIGA-D-18-00397_Revision_2.pdf

Response_to_Reviewer_Comments_Original_Submission.pdf

Response_to_Reviewer_Comments_Revision_1.pdf

Reviewer_1_Report_Original_Submission -- Dokyoon Kim 11/11/2018 Reviewed

Reviewer_1_Report_Revision_1 -- Dokyoon Kim 3/18/2019 Reviewed

Reviewer_2_Report_Original_Submission -- Shefali Setia Verma 11/11/2018 Reviewed

Reviewer_2_Report_Revision_1 -- Shefali Setia Verma 3/14/2019 Reviewed

Supplemental File
