# Supplementary material for: A multi-omics data simulator for complex disease studies and its application to evaluate multi-omics data analysis methods for disease classification
Source: Gigascience. 2019 Apr 26;8(5):giz045. doi: 10.1093/gigascience/giz045 (PMC6486474; doi:10.1093/gigascience/giz045)
Supplement: GIGA-D-18-00397_Original_Submission.pdf [file giz045_giga-d-18-00397_original_submission.pdf]

GigaScience

A multi-omics data simulator for complex disease studies and its application to evaluate multi-omics data analysis methods for disease classification

--Manuscript Draft--

Manuscript Number:	GIGA-D-18-00397
Full Title:	A multi-omics data simulator for complex disease studies and its application to evaluate multi-omics data analysis methods for disease classification
Article Type:	Research
Funding Information:	Ministry of Science and Technology, Taiwan (MOST 106-2221-E-400-005-MY3) — Dr. Ren-Hua Chung
Abstract:	Background<br><br>An integrative multi-omics analysis approach that combines multiple types of omics data including genomics, epigenomics, transcriptomics, proteomics, metabolomics, and microbiomics, has become increasing popular for understanding the pathophysiology of complex diseases. Although many multi-omics analysis methods have been developed for complex disease studies, there is no simulation tool that simulates multiple types of omics data and models their relationships with disease status. Without such a tool, it is difficult to evaluate the multi-omics analysis methods on the same scale and to estimate the sample size or power when planning a new multi-omics disease study.<br><br>Results<br><br>We developed a multi-omics data simulator OmicsSIMLA, which simulates genomics (i.e., SNPs and copy number variations), epigenomics (i.e., whole-genome bisulphite sequencing), transcriptomics (i.e., RNA-seq), and proteomics (i.e., normalized reverse phase protein array) data at the whole-genome level. Furthermore, the relationships between different types of omics data, such as meQTLs (SNPs influencing methylation), eQTLs (SNPs influencing gene expression), and eQTM (methylation influencing gene expression), were modeled. More importantly, the relationships between these multi-omics data and the disease status were modeled as well. We used OmicsSIMLA to simulate a multi-omics dataset for breast cancer under a hypothetical disease model, and used the data to compare the performance among existing multi-omics analysis methods in terms of disease classification accuracy and run time.<br><br>Conclusions<br><br>Our results demonstrated that complex disease mechanisms can be simulated by OmicsSIMLA, and a random forest-based method showed the highest prediction accuracy when the multi-omics data were properly normalized. OmicsSIMLA can be downloaded at https://omicssimla.sourceforge.io.
Corresponding Author:	Ren-Hua Chung<br>National Health Research Institutes<br>Zhunan, Miaoli TAIWAN
Corresponding Author Secondary Information:	
Corresponding Author's Institution:	National Health Research Institutes
Corresponding Author's Secondary Institution:	
First Author:	Ren-Hua Chung
First Author Secondary Information:	
Order of Authors:	Ren-Hua Chung

Powered by Editorial Manager® and ProduXion Manager® from Aries Systems Corporation

	Chen-Yu Kang
**Order of Authors Secondary Information:**	
**Additional Information:**	
**Question**	**Response**
Are you submitting this manuscript to a special series or article collection?	No
**Experimental design and statistics**<br><br>Full details of the experimental design and statistical methods used should be given in the Methods section, as detailed in our Minimum Standards Reporting Checklist. Information essential to interpreting the data presented should be made available in the figure legends.<br><br>Have you included all the information requested in your manuscript?	Yes
**Resources**<br><br>A description of all resources used, including antibodies, cell lines, animals and software tools, with enough information to allow them to be uniquely identified, should be included in the Methods section. Authors are strongly encouraged to cite Research Resource Identifiers (RRIDs) for antibodies, model organisms and tools, where possible.<br><br>Have you included the information requested as detailed in our Minimum Standards Reporting Checklist?	Yes
**Availability of data and materials**<br><br>All datasets and code on which the conclusions of the paper rely must be either included in your submission or deposited in publicly available repositories (where available and ethically appropriate), referencing such data using a unique identifier in the references and in	Yes

the “Availability of Data and Materials” section of your manuscript.<br><br>Have you have met the above requirement as detailed in our Minimum Standards Reporting Checklist?	

Manuscript
Click here to download Manuscript OmicsSIMLA_paper_final.docx

A multi-omics data simulator for complex disease studies and its application to evaluate multi-omics data analysis methods for disease classification

Ren-Hua Chung[1*], Chen-Yu Kang[1]

[1]Division of Biostatistics and Bioinformatics, Institute of Population Health Sciences, National Health Research Institutes, Zhunan, Taiwan

*Corresponding author: Ren-Hua Chung, PhD

Address: No 35, Keyan Road, Zhunan, Miaoli, 350, Taiwan

Tel: 886-37-246-166 #36105

Fax: 886-37-586-467

Email: rchung@nhri.org.tw

Abstract

Background

An integrative multi-omics analysis approach that combines multiple types of omics data including genomics, epigenomics, transcriptomics, proteomics, metabolomics, and microbiomics, has become increasing popular for understanding the pathophysiology of complex diseases. Although many multi-omics analysis methods have been developed for complex disease studies, there is no simulation tool that simulates multiple types of omics data and models their relationships with disease status. Without such a tool, it is difficult to evaluate the multi-omics analysis methods on the same scale and to estimate the sample size or power when planning a new multi-omics disease study.

Results

We developed a multi-omics data simulator OmicsSIMLA, which simulates genomics (i.e., SNPs and copy number variations), epigenomics (i.e., whole-genome bisulphite sequencing), transcriptomics (i.e., RNA-seq), and proteomics (i.e., normalized reverse phase protein array) data at the whole-genome level. Furthermore, the relationships between different types of omics data, such as meQTLs (SNPs influencing methylation), eQTLs (SNPs influencing gene expression), and eQTM (methylation influencing gene expression), were modeled. More importantly, the relationships between these multi-omics data and the disease status were modeled as well. We used OmicsSIMLA to simulate a multi-omics dataset for breast cancer

under a hypothetical disease model, and used the data to compare the performance among existing multi-omics analysis methods in terms of disease classification accuracy and run time.

Conclusions

Our results demonstrated that complex disease mechanisms can be simulated by OmicsSIMLA, and a random forest-based method showed the highest prediction accuracy when the multi-omics data were properly normalized. OmicsSIMLA can be downloaded at https://omicssimla.sourceforge.io.

Keywords

Multi-omics data, complex disease study, simulation tool

Introduction

Complex diseases such as hypertension, type 2 diabetes, and autism are caused by multiple genetic and environmental factors [1]. Genome-wide association studies have identified many genetic variants (i.e., SNPs) associated with the complex diseases. However, it remains difficult to understand the roles of the associated SNPs in the molecular pathophysiology of the disease and how the SNPs interact with other SNPs in a biological network [2]. With the advancement of high-throughput sequencing technology such as next-generation sequencing

(NGS) and massive parallel technology such as mass spectrometry, multiple types of omics data (i.e., multi-omics data) including genomics, epigenomics, transcriptomics, proteomics, metabolomics, and microbiomics are rapidly generated [3]. As a single type of data generally cannot capture the complexity of molecular events causing the disease, an integrative approach to combining the multi-omics data would be ideal to help elucidate the pathophysiology of the disease [2].

Integrative methods to combine multi-omics data for disease studies have been developed rapidly [4-8]. They can be generally classified into two categories: multi-staged and meta-dimensional approaches [9]. The multi-staged approach aims to first identify relationships between the multi-omics data, and then test the associations between the multi-omics data and the phenotype. For example, Jennings et al. [7] constructed a Bayesian hierarchical model consisting of two stages. The first stage partitioned gene expression into factors accounted by methylation, copy number variation (CNV), and other unknown causes. These factors were subsequently used as predictors for clinical outcomes in the second stage model. One advantage of this approach is that the causal relationships between multi-omics data can be modeled. In contrast, the meta-dimensional approach combines the multi-omics data simultaneously. Raw or the transformed data from the multi-omics data are combined into a single matrix for the analysis. This approach allows for a more flexible inference of the

relationships among the multi-omics data, without the assumptions of the causal relationships between these data.

Although many multi-omics analysis methods for disease studies are available, they were generally evaluated by simulations with data generated specifically to the methods. To compare the performance among these methods, it is necessary to use the same simulated multi-omics dataset with disease status. However, current simulation tools for disease studies mainly focused on simulating a certain type of omics data. For example, more than 25 simulators are available for simulating genetic data with phenotypic trait, according to the Genetic Simulation Resources website (https://popmodels.cancercontrol.cancer.gov/gsr/). Tools such as WGBSSuite [10] and pWGBSSimla [11] can simulate whole-genome bisulphite sequencing (WGBS) data in case-control samples. Moreover, tools such as Polyester [12] and SimSeq [13] simulate RNA-seq data with differential gene expression between two groups of samples. To our knowledge, there is currently no simulation tool that is capable of simulating a variety of omics data types and modeling the complex relationships between the data and the disease. Furthermore, sample size estimation when planning a multi-omics study to ensure sufficient power also becomes important [3]. This also requires a simulation tool that simulates realistic multi-omics data structures and models the architecture of the complex disease.

Here, we developed the multi-omics data simulator OmicsSIMLA, which simulates genomics data including SNPs and CNVs, epigenomics data such as the WGBS data, transcriptomics data (i.e., RNA-seq), and proteomics data such as the normalized reverse phase protein array (RPPA) data at a whole-genome level. Furthermore, the relationships between different types of omics data, such as meQTLs (SNPs influencing methylation), eQTLs (SNPs influencing gene expression), and eQTM (methylation influencing gene expression), were modeled. More importantly, the relationships between these multi-omics data and disease status were modeled as well. The disease models in OmicsSIMLA are flexible so that the main effects and/or interaction effects (either risk or protective) of SNPs and CNVs on the disease can be specified. Differential methylation and differential gene and protein expression between cases and controls can also be simulated. We demonstrated the usefulness of OmicsSIMLA by simulating a multi-omics dataset for breast cancer under a hypothetical disease model, and compared the performance among existing multi-omics analysis tools based on the data.

Results

Figure 1 shows the framework of OmicsSIMLA. The genomics data that can be simulated include SNPs and CNVs. Genotypes at SNPs in unrelated and/or family samples are simulated based on the SeqSIMLA2 algorithm [14]. CNV status (i.e., a deletion, normal, one

duplication or two duplications) on a chromosome is simulated based on the user-specified chromosomal regions and CNV frequencies. Affection status of each sample is determined by a logistic penetrance function conditional on the causal SNPs and CNVs, and/or the interactions among the causal SNPs. The epigenomics data are the methylated and total read counts at CpGs based on bisulphite sequencing, simulated using the pWGBSSimla algorithm incorporating methylation profiles for 29 human cell and tissue types [11]. Allele-specific methylation (ASM), in which paternal and maternal alleles have different methylation rates, and differentially methylated region (DMR), where the same CpGs in the region have different methylation rates among different cell types, can also be simulated. Furthermore, the transcriptomics data (i.e., RNA-seq read counts) are simulated with a parametric model assuming a negative-binomial distribution. Finally, the mass-action kinetic action model [15] is used to simulate proteomics data at a certain time point incorporating the gene expression data. Some SNPs can be specified as meQTLs and eQTLs, and some CpGs can be specified as eQTM. Allele-specific expression (ASE), which alleles in a gene have different expression levels, caused by cis-eQTL can also be simulated. The differential methylation, gene expression, and protein expression levels between cases and controls are simulated conditional on the affection status.

Using OmicsSIMLA, we simulated a multi-omics dataset based on hypothetical pathways for

breast cancer as described in Ritchie et al. [9] and illustrated in Figure 2. The data included a deletion with a protective effect in the CYP1A1 gene, 3 common SNPs with risk effects in the CYP1B1 gene, 5 rare SNPs in the COMT gene, which had interaction effects with a meQTL for the XRCC1 gene, and 5 rare SNPs in the GSTM1 gene, which also had interaction effects with an eQTL affecting the gene and protein expression of the XRCC3 gene. Finally, 5 rare SNPs in the GSTT1 gene also had interaction effects with a SNP in a regulatory region. A total of 2,022 SNPs in the four genes (i.e., CYP1B1, COMT, GSTM1, and GSTT1) and a regulatory region consisting of the meQTL, eQTL, and the SNP interacting with GSTT1, 1 CNV in CYP1A1, 688 CpGs in XRCC1, and gene and protein expression levels for 100 genes (including the expression for XRCC3 and 99 other hypothetical genes in the pathways) were simulated. More details about the simulations can be found in the Methods section.

We compared the performance of three multi-omics data analysis methods for disease prediction using the area under the curve (AUC) measures. The three methods included the random forest-based method (RFomics), a graph-based integration method (CANetwork) [5], and a model-based integration method (ATHENA) [4]. The RFomics combines the preprocessed multi-omics data in a single matrix for constructing the prediction model. As described in the Methods section, a gene-based risk score is calculated based on SNPs for

each gene. Then the risk scores and other multi-omics data are normalized so that they can be evaluated on the same scale by the RF algorithm. In contrast, CANetwork calculates a graph matrix to measure the distance between samples using the composite association network algorithm [16], and the prediction model is created based on the distance matrix using the graph-based semi-supervised learning algorithm [17]. Finally, ATHENA creates a neural network model for each type of omics data and a final integrative model is generated based on these models.

Table 1 shows the area under the curve (AUC) for the three methods under three scenarios. Scenario 1 had 500 cases and 500 controls in the training set, and 100 cases and 100 controls in the validation set. Scenario 2 had the same sample sizes as those in Scenario 1, but the multi-omics data had less strong effects on the disease compared to Scenario 1. The effects of the multi-omics data were the same in Scenarios 3 as those in Scenario 1, but Scenarios 3 had larger sample size (i.e., 1,500 cases and 1,500 controls in the training data and 500 cases and 500 controls in the validation data). More details of the three scenarios are provided in the Methods section. Prediction models for the three methods were created based on the training dataset, and their prediction accuracies were evaluated by the validation dataset. As seen in Table 1, RFomics has the highest AUC in all 3 scenarios followed by ATHENA and CANetwork. Table 2 shows the run time for the three methods. In Scenario 1, RFomics and

CANetwork had similar performance, while ATHENA required more than 20-times the runtime of RFomics and CANetwork. In Scenario 3, CANetwork was the most efficient method followed by RFomics, and ATHENA also required significantly more time than the other two methods.

Discussion

We have developed OmicsSIMLA, which simulates multi-omics data (i.e., genomics, epigenomics, transcriptomics, and proteomics data) with disease status. In contrast to the current omics data simulators that mainly focused on simulating one type of omics data, OmicsSIMLA simulates multiple types of omics data while the relationships between different types of omics data and the relationships between the omics data and the disease are modeled. As the development of integrative methods for analyzing multi-omics data has attracted substantial interest from researchers, OmicsSIMLA will be very useful to simulate benchmark datasets for comparisons of these methods. Furthermore, as more and more disease studies take advantages of multi-omics data, OmicsSIMLA will also be very useful for power calculations and sample size estimations when planning a new study.

We used OmicsSIMLA to simulate a multi-omics dataset for breast cancer based on hypothetical pathways. Three analysis tools were compared using the dataset. The results

suggest that when the different types of data were properly normalized on the same scale, the RF-based method (i.e., RFomics) achieved the highest AUC. Furthermore, RFomics had comparable runtime efficiency as that of CANetwork, while ATHENA was computationally expensive. Therefore, RFomics can potentially be a useful analysis tool for disease prediction using multi-omics data.

Currently, OmicsSIMLA focuses on simulating the dichotomous trait (i.e., affection status). As studies for quantitative traits are also important, it is our future work to extend OmicsSIMLA to simulate quantitative traits based on the classic quantitative genetics model [18]. Furthermore, environmental factors and the interactions between genes and environments can also play important roles in complex disease etiology. Therefore, simulating exposome data such as the climate and air quality data and modeling their interactions with genes are also important in the future extensions of OmicsSIMLA.

Conclusions

In conclusion, we developed a useful multi-omics data simulator, OmicsSIMLA, for complex disease studies. Benchmark datasets can be simulated by OmicsSIMLA for evaluating different multi-omics data analysis methods for disease studies. OmicsSIMLA can also be used to estimate sample sizes and statistical power when designing a new multi-omics disease

study. As many parameters can be adjusted in OmicsSIMLA, a user-friendly web interface is provided at https://omicssimla.sourceforge.io/generateCommand.html to conveniently specify these parameters.

Methods

Simulation of DNA sequences

The SeqSIMLA2 package [14] is integrated in OmicsSIMLA to generate DNA sequences in unrelated/related individuals. Similar to SeqSIMLA2, OmicsSIMLA expects a set of external reference sequences (i.e., haplotypes) generated by an external sequence generator, such as COSI [19] or HAPGEN2 [20] that has been widely adopted in genetics studies. Generally, a set of 10,000 or more reference sequences are expected. Optional files consisting of recombination rate information and pedigree structures are also accepted. A gene dropping algorithm assuming random mating with crossovers is performed based on the reference sequences, recombination rates, and pedigree structures to generate haplotypes in each individual.

Simulation of CNVs

For the simulation of CNVs, we considered four CNV states including deletion (D), normal (N), one duplication (U), and two duplications (UU) on a chromosome. Therefore, there are 10 types of CNV states on the two chromosomes in an individual, as

shown in Supplementary Table S1, and the total copy numbers on the two chromosomes range from 0 to 6. The user will provide frequencies and ranges of the four CNV states. During meiosis, we use the single-copy crossover model, assuming all crossovers occurred between CNVs [21].

Simulation of affection status

Genetic variants, including SNPs and CNVs, are used to determine the affection status of an individual based on a logistic penetrance function as follows:

$$\text{logit}(P(affected)) = \beta_0 + \sum_{i \in \Omega} \beta_{C_{i1}} C_{i1} + \sum_{i \in \Omega} \beta_{C_{i2}} C_{i2} + \sum_{j \in \Psi} \beta_{G_j} G_j + \sum_{m,n \in \Upsilon} \beta_{mn} G_{mn}$$

where $P(affected)$ is the probability of being affected, β_0 determines the baseline prevalence, Ω, Ψ, and Υ are sets of causal CNVs, SNPs with main effects, and SNPs with interaction effects, respectively, specified by the user, C_{i1} and C_{i2} are the CNV states for the first and second haplotypes at CNV *i*, respectively, G_j is the genotype coding at SNP *j*, and G_{mn} is the genotype coding at SNPs *m* and *n*. C_{i1} *and* C_{i2} have values of -1, 0, 1, and 2 for CNV states *D*, *N*, *U*, and *UU*, respectively, where *N* is the baseline state. The coding of G_j is based on a dominant, additive or recessive model, and the coding of G_{mn} is based on several interaction models. If SNP *j* is in a CNV region, allelic CNV [22] is considered in the coding of G_j. More details of the coding of G_j and G_{mn} are provided in Supplementary methods. The parameters β_C and β_G are the effect sizes of the main effects for CNVs and SNPs, respectively, and β_{mn} determines the effect size of the interaction effect between SNPs *m*

and *n*. These parameters are specified by the user.

Simulation of DNA methylation data

The pWGBSSimla package [11] is integrated into OmicsSIMLA to generate the WGBS data. The pWGBSSimla algorithm simulates data using methylation profiles generated based on 41 WGBS datasets for 29 human cell and tissue types. The profiles contain the information for each CpG, such as its distance to the next site, methylation rate, methylation status (i.e., methylated, unmethylated, and fuzzily methylated), and read counts for each type of methylation status. CpGs and the distances between the CpGs are first determined based on the profiles, and then the total read count and methylated read count are simulated for each CpG. Methylation level at a CpG influenced by a meQTL is simulated based on a genotype-specific methylation probability, which is the methylation rate of the CpG in the profiles multiplied by a ratio following an exponential distribution. Furthermore, ASMs are simulated based on father- and mother-specific methylation rates for paternal and maternal alleles, respectively. Finally, a DMR is generated by simulating the same genomic region using profiles for different cell or tissue types. More details of the pWGBSSimla algorithm can be found in Chung and Kang [11].

Simulation of RNA-seq data

We implemented a parametric simulation procedure for simulating the RNA-seq data similar to that described in Benidt and Nettleton [13]. A negative binomial (NB)

distribution with mean μ_{ij} and dispersion parameter ω_i is used to simulate the read count for gene *i* in individual *j*. The mean is calculated as $\mu_{ij} = \lambda_i c_j$, where λ_i is the common mean for gene *i* and c_j is the individual-specific normalization factor for individual *j*. The parameters $\boldsymbol{\lambda}$, $\boldsymbol{c}$, and $\boldsymbol{\omega}$ for all genes were estimated using the R package edgeR [23] based on a whole genome RNA-seq dataset consisting of 103 normal tissues in patients with breast cancer from The Cancer Genome Atlas (TCGA) project [24]. The parameters λ_i and ω_i are randomly sampled with replacement from $\boldsymbol{\lambda}$ and $\boldsymbol{\omega}$. If more than 103 samples are simulated, we use the smoothed bootstrap procedure [25] to calculate c_j^* for individual *j*, and μ_{ij} is calculated as $\lambda_i c_j^*$. More details of the calculation of c_j^* are provided in Supplementary methods. The user can specify *n* differentially expressed (DE) genes between cases and controls and their fold changes, and the read count for DE gene *i* in individual *j* is simulated based on a NB distribution with mean $f_i \mu_{ij}$ and dispersion parameter ω_i, where f_i is the fold change for gene *i*.

Simulation of eQTL and allele-specific reads

We followed the procedure in the simulation study in Sun [26] to simulate eQTL and read counts for ASE. For eQTL *l* with a user-specified fold change h_l, the means for the three genotypes *AA*, *Aa*, and *aa* at the eQTL are μ_{ij}, $h_l \mu_{ij}$, and $(2h_l - 1)\mu_{ij}$, respectively, and the dispersion parameter is ω_i in the NB distribution for gene *i* influenced by the eQTL. ASE for a gene caused by a cis-eQTL is simulated by assuming reads were mapped to

heterozygous SNPs (i.e., allele-specific reads) in the gene. A cis-eQTL refers to the eQTL being located in the cis-regulatory elements of the gene. Because the alleles at the cis-eQTL can be in the same haplotype as the alleles of the gene, ASE can be observed using the allele-specific reads of the gene. Furthermore, only heterozygous SNPs can be tested for cis-eQTL with the allele-specific reads. Therefore, we simulate allele-specific reads for heterozygous eQTLs. Assuming t_{ij} is the total read count for gene *i* in individual *j*, the total number of allele-specific reads is calculated as $0.005t_{ij}$, where 0.005 was estimated from real data by Sun [26]. Furthermore, also suggested by Sun [26], the number of allele-specific reads for a haplotype is simulated using a beta-binomial distribution with a mean determined by the effect size of the cis-eQTL and an overdispersion parameter of 0.1. The effect size is defined as $\log_2$(expression of the alternative allele at the eQTL/expression of the reference allele at the eQTL) [27] for a heterozygous cis-eQTL and is set to 0 for a homozygous cis-eQTL.

Simulation of eQTM

We used linear regression to model the relationship between gene expression and methylation:

$\mu_i^{'} = E(y_{ij}) = \alpha_i + \beta_i x_{ij}$, where y_{ij} and x_{ij} are the RNA-seq read count and the proportion of methylated reads, respectively, for gene *i* influenced by methylation in individual *j*. Assuming that the NB parameters for gene *i* are μ_i and ϕ_i, the parameter α_i is specified as μ_i, and β_i is assumed to follow a normal distribution with a mean and a standard deviation specified

by the user. Then the gene expression of gene *i* is simulated by an NB distribution with parameters of $\mu_i^{'}$ and ϕ_i.

Protein expression simulation

We assumed that the protein expression level for protein *k* at a time point *t* in sample *j* follows a normal distribution with a mean η_{kjt} and a standard deviation τ_k after normalization. We used the mass-action kinetic action model [15] to simulate protein expression at a certain time point. The mean $\eta_{kj,t+1}$ for the protein expression at time *t*+1 was determined as follows:

$$\eta_{kj,t+1} = \eta_{kjt} + (x_{kjt}\kappa_{jt}^{s} - \eta_{kjt}\kappa_{jt}^{d}),$$

where x_{kjt} is the normalized gene expression for the gene encoding protein *k*, and κ_{jt}^{s} and κ_{jt}^{d} are the protein synthesis and degradation rates, respectively, in individual *j* at time *t*. The normalized gene expression x_{kjt} is calculated using the median absolute deviation (MAD) scale normalization [28] based on the RNA-seq data simulated from the previous section. Similar to the simulation study in Teo et al. [15], κ_{jt}^{d} is fixed to be 1, and κ_{jt}^{s} with a default value of 1 can be changed by the user. A vector of standard deviations $\boldsymbol{\tau}$ were estimated from the level 4 protein expression data of primary tumor tissue in 874 breast cancer patients from the TCGA project downloaded from the cancer proteome atlas (TCPA) [29] website. The level 4 data consist of protein expression data for 224 proteins that have been normalized across the samples as well as across the proteins, and a replication-based method was used to

account for differences in protein expression among different batches. The parameter τ_j is then randomly sampled with replacement from $\boldsymbol{\tau}$.

A random-forest based method for integrating multi-omics data for disease studies

Multi-omics data can have different data types (e.g., discrete data for SNP genotypes, categorical data for CNV statuses, and continuous data for proportions of methylated reads, RNA-seq read counts, and normalized protein expression) and different variations (e.g., three possible values of 0, 1, and 2 for minor allele counts at SNPs, and real numbers ranging between 0 and 1 for the proportions of methylated reads). When developing a method for integrating these data, it is important to account for the properties of different data types so that the analysis results would not be biased toward certain variables [9]. We developed a preprocessing algorithm for the multi-omics data. A gene-based risk score, which is a weighted sum of the numbers of risk alleles at SNPs in the gene, for each individual is constructed. The weights are the effect sizes of the risk alleles at the SNPs. More details for calculating the risk score are provided in Supplementary methods. Then each variable from different omics data, including the gene-based risk scores, CNV statuses of genes, methylation proportions at CpGs, gene and protein expression levels, is normalized so that it has a mean 0 and a standard deviation of 1. The normalized variables are then used in RF for classification.

Simulation studies

We used OmicsSIMLA to evaluate the performance of the proposed RF-based method, compared with CANetwork and ATHENA. A hypothetical disease model for breast cancer involving multi-omics data [9] was simulated, as shown in Figure 2. To be more specific, a deletion with a frequency of 20%, which had a protective effect with an odds ratio (OR) of 0.67, in the CYP1A1 gene and 3 common variants, which had main effects (ORs = 1.5) with minor allele frequencies (MAFs) > 10%, in the CYP1B1 gene were simulated. We also simulated 5 rare variants with MAFs < 3% in the COMT gene, which had interaction effects (ORs = 5) with a meQTL for the XRCC1 gene. The CpG in XRCC1 influenced by the meQTL caused a difference in methylation rates of 10% between cases and controls. Furthermore, we simulated 5 rare variants in the GSTM1 gene, which had interaction effects (ORs = 5) with a cis-eQTL for the XRCC3 gene, and 5 rare variants in the GSTT1 gene, which had interaction effects (ORs = 5) with a SNP located in the same region as that of the meQTL and eQTL. The eQTL caused a fold change of 1.5 in the XRCC3 gene expression compared to the reference genotype, and a fold change of 1.5 was simulated for the differential gene expression of XRCC3 between cases and controls. In summary, the total variables consisted of 200, 687, 264, and 176 SNPs in the CYP1B1, COMT, GSTM1, and GSTT1 genes, respectively, and 695 SNPs harboring the meQTL, eQTL, and the SNP interacting with GSTT1 in the regulatory region, a variable for CNV status in CYP1A1, methylation levels at 688 CpGs in XRCC1, and gene and protein expression levels for 100

genes and their encoded proteins. More details for generating the reference sequences in the genes and the simulations for each omics data type are provided in Supplementary methods.

We simulated a training dataset consisting of 500 cases and 500 controls as well as a validation dataset consisting of 100 cases and 100 controls. The training dataset was used by RFomics, CANetwork, or ATHENA to construct a prediction model. The validation dataset was then used to calculate the AUC based on the prediction model. Note that a 5-fold cross-validation was performed in ATHENA, and a best model based on the testing dataset (i.e., one of the five random 20% of the training dataset) was created for each cross-validation. The model with the highest AUC based on the testing dataset was selected and applied to the validation dataset. This simulation scenario was referred to as Scenario 1. We also simulated a scenario with less strong genetic effects (Scenario 2) and a scenario with larger sample size (Scenario 3). More details about Scenarios 2 and 3 are provided in Supplementary methods. For each scenario, 1,000 batches of training and validation datasets were simulated, and the AUC for each algorithm was averaged over the 1,000 batches.

Availability of supporting source code and requirements

Project name: OmicsSIMLA

Project home page: https://omicssimla.sourceforge.io

Operating system: Linux

Programming language: C++

Other requirements: C++11 compiler and Eigen and boost libraries if directly compiling the source code.

License: GPL-3.0

Availability of supporting data

The simulated datasets supporting the conclusions of this article are available from the OmicsSIMLA website (https://omicssimla.sourceforge.io/download.html).

Declarations

Funding

This work has been supported by a grant from the Ministry of Science and Technology (MOST 106-2221-E-400-005-MY3) in Taiwan.

Authors' contributions

RHC and CYK both designed the framework of the simulation tool and implemented the software. RHC designed the simulation study and CYK performed the simulation analysis. Both authors read and approved the final manuscript.

Competing interests

The authors declare that they have no competing interests.

References

1. Timpson NJ, Greenwood CMT, Soranzo N, Lawson DJ and Richards JB. Genetic architecture: the shape of the genetic contribution to human traits and disease. Nature reviews Genetics. 2018;19 2:110-24. doi:10.1038/nrg.2017.101.
2. Karczewski KJ and Snyder MP. Integrative omics for health and disease. Nature reviews Genetics. 2018;19 5:299-310. doi:10.1038/nrg.2018.4.
3. Hasin Y, Seldin M and Lusis A. Multi-omics approaches to disease. Genome biology. 2017;18 1:83. doi:10.1186/s13059-017-1215-1.
4. Holzinger ER, Dudek SM, Frase AT, Pendergrass SA and Ritchie MD. ATHENA: the analysis tool for heritable and environmental network associations. Bioinformatics. 2014;30 5:698-705. doi:10.1093/bioinformatics/btt572.
5. Yan KK, Zhao H and Pang H. A comparison of graph- and kernel-based -omics data integration algorithms for classifying complex traits. BMC bioinformatics. 2017;18 1:539. doi:10.1186/s12859-017-1982-4.
6. Ruffalo M, Koyuturk M and Sharan R. Network-Based Integration of Disparate Omic Data To Identify "Silent Players" in Cancer. PLoS computational biology. 2015;11 12:e1004595. doi:10.1371/journal.pcbi.1004595.
7. Jennings EM, Morris JS, Carroll RJ, Manyam GC and Baladandayuthapani V. Bayesian methods for expression-based integration of various types of genomics data. EURASIP J Bioinform Syst Biol. 2013;2013 1:13. doi:10.1186/1687-4153-2013-13.
8. Tyekucheva S, Marchionni L, Karchin R and Parmigiani G. Integrating diverse genomic data using gene sets. Genome biology. 2011;12 10:R105. doi:10.1186/gb-2011-12-10-r105.
9. Ritchie MD, Holzinger ER, Li R, Pendergrass SA and Kim D. Methods of integrating data to uncover genotype-phenotype interactions. Nature reviews Genetics. 2015;16 2:85-97. doi:10.1038/nrg3868.
10. Rackham OJ, Dellaportas P, Petretto E and Bottolo L. WGBSSuite: simulating whole-genome bisulphite sequencing data and benchmarking differential DNA methylation analysis tools. Bioinformatics. 2015;31 14:2371-3. doi:10.1093/bioinformatics/btv114.
11. Chung R-H and Kang C-Y. pWGBSSimla: a profile-based whole-genome bisulphite sequencing data simulator incorporating methylation QTLs, allele-specific methylations and differentially methylated regions. bioRxiv. 2018; doi:10.1101/390633.
12. Frazee AC, Jaffe AE, Langmead B and Leek JT. Polyester: simulating RNA-seq datasets with differential transcript expression. Bioinformatics. 2015;31 17:2778-84. doi:10.1093/bioinformatics/btv272.
13. Benidt S and Nettleton D. SimSeq: a nonparametric approach to simulation of RNA-

sequence datasets. Bioinformatics. 2015;31 13:2131-40. doi:10.1093/bioinformatics/btv124.

14. Chung RH, Tsai WY, Hsieh CH, Hung KY, Hsiung CA and Hauser ER. SeqSIMLA2: simulating correlated quantitative traits accounting for shared environmental effects in user-specified pedigree structure. Genetic epidemiology. 2015;39 1:20-4. doi:10.1002/gepi.21850.
15. Teo G, Vogel C, Ghosh D, Kim S and Choi H. A Mass-Action-Based Model for Gene Expression Regulation in Dynamic Systems. Cambridge University Press; 2015.
16. Mostafavi S, Ray D, Warde-Farley D, Grouios C and Morris Q. GeneMANIA: a real-time multiple association network integration algorithm for predicting gene function. Genome biology. 2008;9 Suppl 1:S4. doi:10.1186/gb-2008-9-s1-s4.
17. Tsuda K, Shin H and Scholkopf B. Fast protein classification with multiple networks. Bioinformatics. 2005;21 Suppl 2:ii59-65. doi:10.1093/bioinformatics/bti1110.
18. Falconer DS and Mackay TF. Quantitative genetics. San Francisco: Benjamin Cummings; 1996.
19. Schaffner SF, Foo C, Gabriel S, Reich D, Daly MJ and Altshuler D. Calibrating a coalescent simulation of human genome sequence variation. Genome research. 2005;15 11:1576-83. doi:10.1101/gr.3709305.
20. Su Z, Marchini J and Donnelly P. HAPGEN2: simulation of multiple disease SNPs. Bioinformatics. 2011;27 16:2304-5. doi:10.1093/bioinformatics/btr341.
21. Hartasanchez DA, Valles-Codina O, Braso-Vives M and Navarro A. Interplay of interlocus gene conversion and crossover in segmental duplications under a neutral scenario. G3. 2014;4 8:1479-89. doi:10.1534/g3.114.012435.
22. Usher CL and McCarroll SA. Complex and multi-allelic copy number variation in human disease. Briefings in functional genomics. 2015;14 5:329-38. doi:10.1093/bfgp/elv028.
23. Robinson MD, McCarthy DJ and Smyth GK. edgeR: a Bioconductor package for differential expression analysis of digital gene expression data. Bioinformatics. 2010;26 1:139-40. doi:10.1093/bioinformatics/btp616.
24. Cancer Genome Atlas Research N. Comprehensive genomic characterization defines human glioblastoma genes and core pathways. Nature. 2008;455 7216:1061-8. doi:10.1038/nature07385.
25. Efron B and Tibshirani RJ. An Introduction to the Bootstrap. Chapman and Hall/CRC; 1993.
26. Sun W. A statistical framework for eQTL mapping using RNA-seq data. Biometrics. 2012;68 1:1-11. doi:10.1111/j.1541-0420.2011.01654.x.
27. Mohammadi P, Castel SE, Brown AA and Lappalainen T. Quantifying the regulatory effect size of cis-acting genetic variation using allelic fold change. Genome research.

2017;27 11:1872-84. doi:10.1101/gr.216747.116.

28. Fundel K, Kuffner R, Aigner T and Zimmer R. Normalization and gene p-value estimation: issues in microarray data processing. Bioinform Biol Insights. 2008;2:291-305.

29. Li J, Lu Y, Akbani R, Ju Z, Roebuck PL, Liu W, et al. TCPA: a resource for cancer functional proteomics data. Nature methods. 2013;10 11:1046-7. doi:10.1038/nmeth.2650.

Figures

Figure 1. Simulation framework of OmicsSIMLA. The black solid lines represent the relationships among different types of omics data. The black dotted lines represent the causal effects of genomics data to the disease. The red dotted lines represent the retrospective simulations of the methylation, gene expression and protein expression levels conditional on the disease status.

Figure 2. Hypothetical pathways involved in breast cancer. The brown solid lines represent the main effects of SNPs and CNVs on the disease, while the green solid lines represent the interaction effects of SNPs on the disease. The black sold lines represent the regulatory effects of the meQTL and eQTL on methylation and gene expression, respectively. The red dotted lines represent the retrospective simulations of the methylation, gene expression and protein expression levels conditional on the disease status.

Tables

Table 1. Area under the curve (AUC) for RFomics, CANetwork, and ATHENA under different scenarios

	RFomics	CANetwork	ATHENA
Scenario 1	0.861 (0.026)[1]	0.596 (0.042)	0.831 (0.042)
Scenario 2	0.566 (0.041)	0.529 (0.029)	0.559 (0.068)
Scenario 3	0.876 (0.012)	0.649 (0.019)	0.835 (0.031)

[1]The mean AUC and its standard error estimated based on 1,000 batches

Table 2. Run time (in seconds) for RFomics, CANetwork, and ATHENA under Scenarios 1 and 3

	RFomics	CANetwork	ATHENA
Scenario 1	37.78	40.54	823.14
Scenario 3	143.91	94.16	2195.95

[1]The mean time (in seconds) was estimated based on 100 batches

Click here to download Figure Figure1.jpg

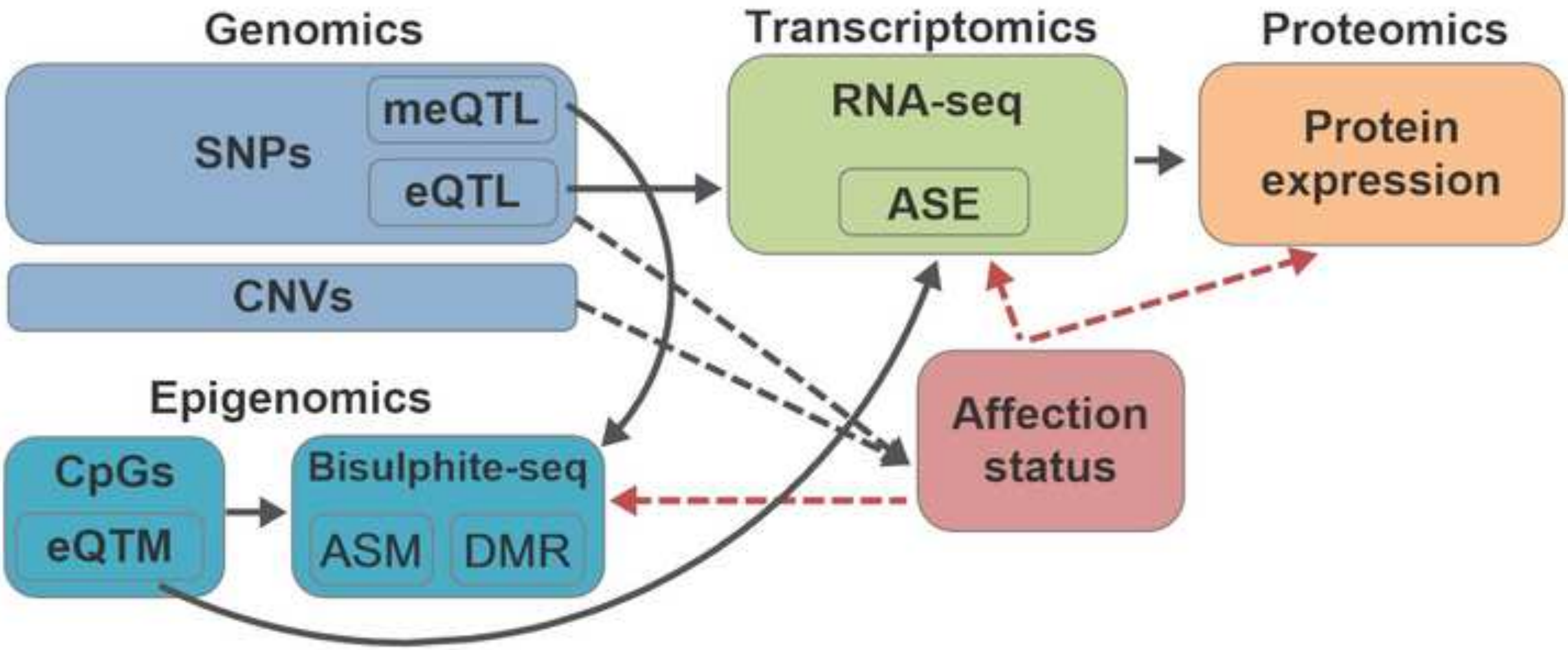

Figure 2

Click here to download Figure Figure2.jpg

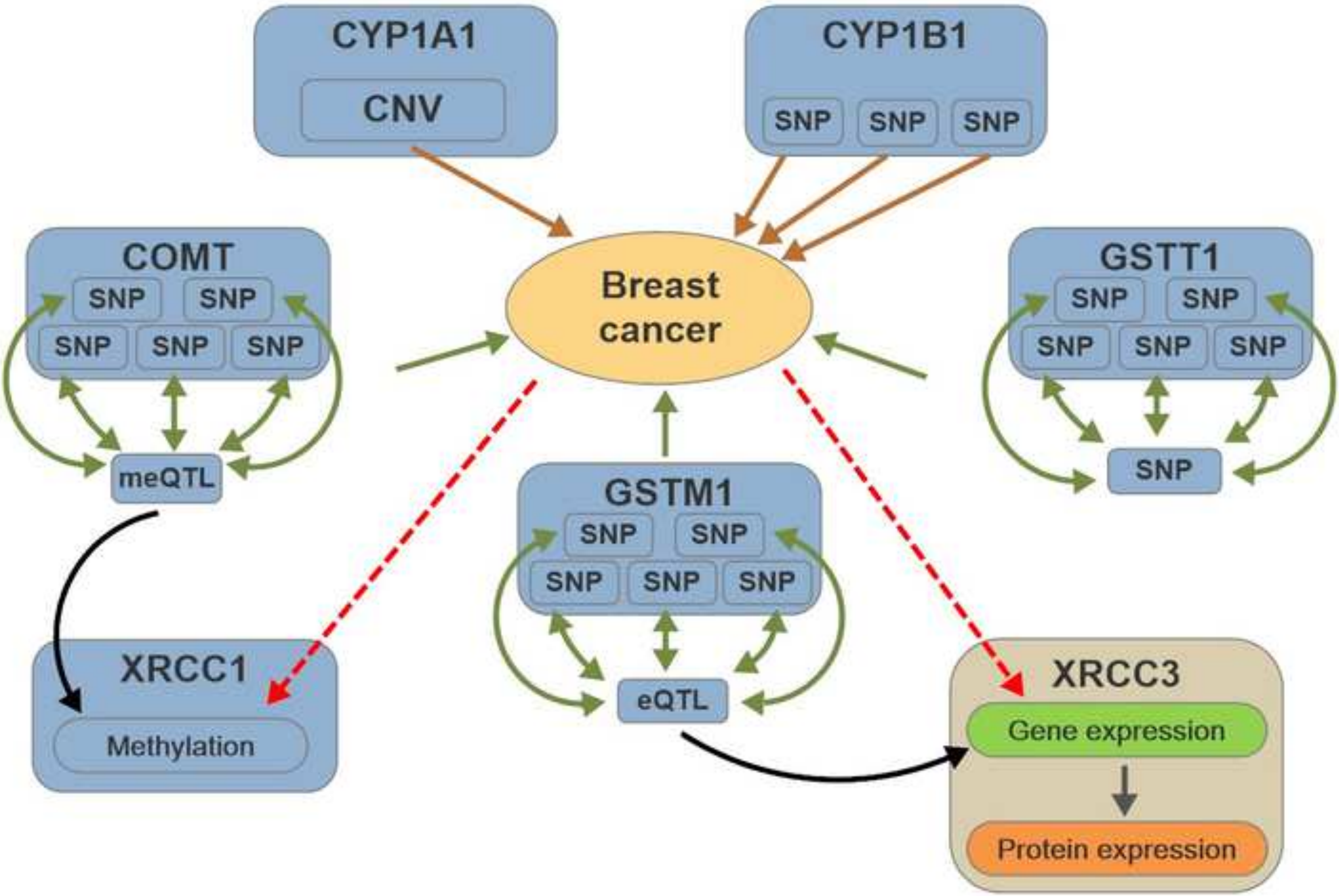

Click here to access/download
Supplementary Material
Supplementary_material.docx
