# Supplementary material for: A multi-omics data simulator for complex disease studies and its application to evaluate multi-omics data analysis methods for disease classification
Source: Gigascience. 2019 Apr 26;8(5):giz045. doi: 10.1093/gigascience/giz045 (PMC6486474; doi:10.1093/gigascience/giz045)
Supplement: GIGA-D-18-00397_Revision_1.pdf [file giz045_giga-d-18-00397_revision_1.pdf]

GigaScience

A multi-omics data simulator for complex disease studies and its application to evaluate multi-omics data analysis methods for disease classification

--Manuscript Draft--

**Manuscript Number:**	GIGA-D-18-00397R1
**Full Title:**	A multi-omics data simulator for complex disease studies and its application to evaluate multi-omics data analysis methods for disease classification
**Article Type:**	Research
**Funding Information:**	Ministry of Science and Technology, Taiwan (MOST 106-2221-E-400-005-MY3) — Dr. Ren-Hua Chung
**Abstract:**	Background<br><br>An integrative multi-omics analysis approach that combines multiple types of omics data including genomics, epigenomics, transcriptomics, proteomics, metabolomics, and microbiomics, has become increasing popular for understanding the pathophysiology of complex diseases. Although many multi-omics analysis methods have been developed for complex disease studies, only a few simulation tools that simulate multiple types of omics data and models their relationships with disease status are available and these tools have their limitations in simulating the multi-omics data.<br><br>Results<br><br>We developed a multi-omics data simulator OmicsSIMLA, which simulates genomics (i.e., SNPs and copy number variations (CNVs)), epigenomics (i.e., bisulphite sequencing), transcriptomics (i.e., RNA-seq), and proteomics (i.e., normalized reverse phase protein array) data at the whole-genome level. Furthermore, the relationships between different types of omics data, such as meQTLs (SNPs influencing methylation), eQTLs (SNPs influencing gene expression), and eQTM (methylation influencing gene expression), were modeled. More importantly, the relationships between these multi-omics data and the disease status were modeled as well. We used OmicsSIMLA to simulate a multi-omics dataset for breast cancer under a hypothetical disease model, and used the data to compare the performance among existing multi-omics analysis methods in terms of disease classification accuracy and runtime. We also used OmicsSIMLA to simulate a multi-omics dataset with a scale similar to an ovarian cancer multi-omics dataset. The neural network-based multi-omics analysis method, ATHENA, was applied to both the real and simulated data and the results were compared.<br><br>Conclusions<br><br>Our results demonstrated that complex disease mechanisms can be simulated by OmicsSIMLA, and ATHENA showed the highest prediction accuracy when the effects of multi-omics features (e.g., SNPs, CNVs, and gene expression levels) on the disease were strong. Furthermore, similar results can be obtained from ATHENA when analyzing the simulated and real ovarian multi-omics data.
**Corresponding Author:**	Ren-Hua Chung<br>National Health Research Institutes<br>Zhunan, Miaoli TAIWAN
**Corresponding Author Secondary Information:**	
**Corresponding Author's Institution:**	National Health Research Institutes
**Corresponding Author's Secondary Institution:**	
**First Author:**	Ren-Hua Chung

Powered by Editorial Manager® and ProduXion Manager® from Aries Systems Corporation

First Author Secondary Information:	
Order of Authors:	Ren-Hua Chung
	Chen-Yu Kang
Order of Authors Secondary Information:	
Response to Reviewers:	Editor's comments:<br>1.The manuscript addresses an important problem. However, overall, you need to provide more practical examples in order to demonstrate its validity. Because there are several papers that already demonstrate the data integration effect predicting outcomes in a TCGA dataset, we ask that you simulate multi-omics data using OmicsSIMLA and compare the results between the simulation data and real dataset based on previous literature. You also need to highlight how OmicsSIMLA compares to other similar tools, for example, HIBACHI (https://www.ncbi.nlm.nih.gov/pmc/articles/PMC4270828/).<br>Response:<br>Thank the Editor for the comments. We used OmicsSIMLA to simulate a multi-omics dataset based on the ovarian cancer (OV) data, consisting of CNVs, methylation, gene expression, and protein expression data, downloaded from the TCGA project. More details can be found in the response to the first comment of Reviewer 1. We have also highlighted how OmicsSIMLA compares to other similar tools, which can be seen in the response to the first comment of Reviewer 2.<br><br>2.In addition, please register any new software application in the SciCrunch.org database to receive a RRID (Research Resource Identification Initiative ID) number, and include this in your manuscript. This will facilitate tracking, reproducibility and re-use of your tool.<br>Response:<br>We have registered our software in the SciCrunch database with a RRID of SCR_017011. We have also included the RRID in the manuscript.<br><br>Reviewer 1's comments:<br>1.The evaluation scheme for the proposed simulation tool is not strong. Simulating multi-omics data based on the hypothetical example is great, but they should provide more practical examples in order to demonstrate its validity. There have been many papers that show the data integration effect predicting outcomes using TCGA dataset. So, I would suggest that they should simulate multi-omics data using OmicsSIMLA and compared the results between the simulation data and real dataset based on the previous literature.<br>Response:<br>Thank the Reviewer for the very constructive comments. We have used OmicsSIMLA to simulate a multi-omics dataset based on the ovarian cancer (OV) data, consisting of CNVs, methylation, gene expression, and protein expression data, downloaded from the TCGA project. We first applied ATHENA to the TCGA OV data for classifying the survival time (i.e., long-term and short-term survival) similar to the analysis in Kim et al. (BioData Mining 2013). We then simulated a multi-omics data based on the OV data with the model identified by ATHENA. Finally, ATHENA was applied to the simulated data. Our analysis results demonstrated that OmicsSIMLA can generate data with a scale similar to the OV data, and the analysis results from ATHENA between the real and simulated data were comparable. The results for the simulations are described starting from the last paragraph on page 14, and how we performed the simulations is described from page 29.<br><br>2.As they introduced multi-staged and meta-dimensional approaches in data integration, they need to show the simulation results based on two different approaches (scenarios). Researchers have been developing data integration methods based on either multi-staged or meta-dimensional models, they might need OmicsSIMLA with different purpose.<br>Response:<br>We have included a three-staged method (Holzinger and Ritchie, Pharmacogenomics, 2012) in our simulation studies to compare with the meta-dimensional approaches. The implementation of the three-stage method is described in the last paragraph on page 12 and the Supplementary methods. The results for the comparisons of the three-staged and meta-dimensional approaches are described starting from the last

paragraph on page 13.

3.It is good to use three different machine learning methods, but specific sets of parameters (such as population size, generation number, migration, etc for ATHENA) per method are missing. Depending on different sets of parameters, results can be very different. They should provide this info in the methods section.
Response:
We have provided the parameter values we used for simulations for ATHENA and random forest in Supplementary Table S1. The Reviewer is correct that the analysis results can be very different depending on different sets of parameters. When we increased the number of demes, the number of generations, and the number of migrations in ATHENA, the AUC for ATHENA increased significantly, as shown in Table 1, and ATHENA now showed the highest AUC compared to other methods. The results are described in the last paragraph on page 13.

4.So, it is the classification problem, but what it eh label?
Response:
The label is the disease status. We have clarified this in the second paragraph on page 12.

5.I think there is a wrong subsection header name in the method section. For the normalization section: A random-forest based method for integrating multi-omics data for disease studies?
Response:
The section was intended to describe how we normalized data for random forest. Since we have now emphasized that ATHENA would be the best tool to perform multi-omics data analysis, we have moved the section to the Supplementary methods and integrated the section with “Calculating the risk score of a gene in RFomics.”

6.The manual was well described. It would be great if they add the tutorial for the hypothetical example as well as additional scenarios based on TCGA real dataset.
Response:
We have added the tutorials for the hypothetical example and the scenario based on TCGA real dataset in the user manual, which can be accessed here: https://omicssimla.sourceforge.io/simuomicsTCGA.html.

Reviewer 2’s comments:
1.This manuscript presents a very interesting expansion to existing simulation tools by adding various 'omics simulations possible. This is definitely a requirement in the field, though I want to mention that there are other tools like HIBACHI (https://www.ncbi.nlm.nih.gov/pmc/articles/PMC4270828/) which also aim to achieve similar integrated framework to mimic the biology and interactions among various omic datasets to simulate the data. Authors must revise the sentences to mention how OmicSIMLA is different and also is not the first comics simulator.
Response:
Thank the Reviewer for the very constructive comments. We have revised the statement and reviewed the currently available multi-omics data simulators, such as HIBACHI, InterSIM, and MOSim. The review is provided in the second paragraph on page 6. Furthermore, we discussed the advantages and disadvantages of using these tools starting from the last paragraph on page 16.

2.Authors do a nice job of breaking down what the required input files are for simulating each specific data type (genotype, CNV, WGBSS, gene expression, and protein expression); however, the role of "Profiles" is a bit unclear. For example, gene expression "Profile" data contains gene expression beta values, but whether a user is to input their own beta values or if there is a standard is unclear. Additionally, the gene expression profile data contains individual-specific normalization factors (mean and dispersion parameters) to create the negative binomial distribution. The file contains values for 103 normal tissue but there is no information on how to actually use or interpret this data. Please add more clarity on the same.
Response:
The beta values in the gene expression profiles are no longer used. We have removed them from the gene expression profiles. We have explained in more details about how to use or interpret the individual-specific normalization factors. The individual-specific

normalization factor is used to model the systematic variations among individuals due to technical variation. If no technical variation will be simulated, the user can replace c with a vector of 1, where the length of the vector is the number of samples to be simulated. We have clarified the use and interpretation of the normalization factors and the parameters for the negative binomial distributions in the last paragraph on page 23.

3.There is methylation profile data which is specific to 29 cell types and tissues. This is quite easy to maneuver when generating methylation simulations. There is a protein expression profile which contains standard deviations for the normal distribution. The parameters are based on 874 breast cancer patients from the TCPA. If the gene expression and protein expression profiles were also based on tissue type (maybe something like GTEx), that would be really useful.
Response:
Thank the Reviewer for the suggestion. We have compiled profiles of different cancer tissue types for the CNV, gene expression, and protein expression profiles. To be more specific, the CNV profiles are specific to 33 cancer tissues, the gene expression profiles are specific to normal and tumor tissues for 31 cancers, and the protein expression profiles are specific to 26 cancers. These profiles were compiled based on the TCGA data. We described the profiles in the last paragraph on page 9 and how we generated the profiles in the Supplementary Methods.

4.To summarize above two points, more details about the role and application of "profile" data would be helpful and why the data is skewed for certain distributions. Do users need to use the same profiles to generate simulations, and if not, does their data need to fit a certain distribution?
Response:
We provided profiles for simulating the CNV, methylation, gene expression, and protein expression data. To use the profiles, the user simply specify the tissue types and the number of samples to be simulated and OmicsSIMLA will simulate datasets based on the parameters in the profiles. Alternatively, the user can compile their own profiles with the formats and assumptions of data distributions that are clearly described in the user manual. For example, negative binomial distributions are assumed for the RNA-seq read counts, and normal distributions are assumed for the protein expression data. We described the use of the profiles starting from the last paragraph on page 9.

5) For the input file in SeqSIMLA, a variety of reference files can be input to bolster specificity of the simulated genotypes, i.e. refseq, penetrance, pedigree structure, etc. Most of these are in PLINK file format so that is good. However, what is a bit unclear is how much of this information is required to generate simulated data. For example, in HIBACHI, you could specify the bare minimum parameters to generate a simulation, of course with the drawback being that the data had no biological context. Here it is evident the data has biological context, but there should be more information for use case; how much/which type of data is required to generate the genotype simulation? It is not very clear what is "required" and what is "optional".
Response:
As there are many input files and parameters in OmicsSIMLA, some are required or optional depending on the simulation scenarios. The best way for the user to learn the required and optional files and parameters in OmicsSIMLA is to use the web interface (https://omicssimla.sourceforge.io/generateCommand.html). The required fields are marked with red * on the web interface. If certain simulations are selected by the user, more options will appear for inputs. The interface will also check the inputs from the user. If some required fields are missing from the inputs, the interface will remind the user. We have described the input files and parameters for OmicsSIMLA starting from the last paragraph on page 9 to page 11. On page 11, we encourage the user to use the web interface to learn the required and optional files and parameters for OmicsSIMLA.

6) It appears that this tool is best used if you have datasets already but are missing a specific desired dataset. For example, if you have genotype and methylation, and you know the data well enough to expect values for missing protein expression data type. Or if you have very specific requirements for the type of simulated data you want. This tool doesn't seem like the best option if you are simulating all datatypes from scratch, authors should clarify if this is the case because this point is not mentioned in the manuscript. And if this is not the case, more clarification on how all data types can be

Powered by Editorial Manager® and ProduXion Manager® from Aries Systems Corporation

	generated from scratch would be very useful.<br>Response:<br>Three main components are required to perform the simulations in OmicsSIMLA, including a biological model (i.e., the disease model), reference sequences and profiles, and parameter values to model the effects of the multi-omics features on the disease and to model the relationships between multi-omics data. A biological model should be hypothesized by the user before performing the simulations. We have compiled the reference sequences for different human populations and profiles for many tissue types so that the user can conveniently choose the appropriate files for the simulations. Furthermore, we have made suggestions for many of the parameter values based on the literatures or the observations in real data. The pre-compiled files and recommended values will minimize the efforts for the user to perform the simulation from scratch. We have discussed the best use of OmicsSIMLA starting from the last paragraph on page 18.<br><br>7) Another example of the last comment is that for the tutorial, they provide the following 3 files: 1) a reference .bim file containing 2022 SNPs, 2) a .txt file specifying site interaction effects between SNPs using an odds ratio, and 3) a .txt file containing information on CNVs between sites and the frequency of deletions/odds ratio of deletion. Therefore, it seems to me you are required to have a good amount of input data in order to generate the simulations.<br>Response:<br>Same as the response to the previous comment, we have pre-compiled several files to minimize the efforts for the user to generate the simulations.<br><br>8) For simulating gene expression data, it seems that beta values are required. It would be beneficial to mention where to obtain beta values, should these always be constant from public resources like GTex?<br>Response:<br>The beta values are no longer used. We have removed them from the profiles.<br><br>9) I used the tutorials to generate simulations: for the most part they were as expected. However, when simulating multi-omics data, I was unable to generate a .cnv file as is outlined in the tutorial under "Several files will be generated:".<br>Response:<br>We have executed the command again as described in the tutorials and it seemed that the cnv file can be generated. Please contact us for any technical supports to simulate the file.<br><br>10) Additionally, upon generating successful simulations, I received the following error every time:<br>sh: ../bin/bug_reporter: cannot execute binary file<br>However, I have not found any documentation on bug_reporter after compilation. I am not sure exactly what is meant by the error.<br>Response:<br>We have fixed the bug.
**Additional Information:**	
**Question**	**Response**
Are you submitting this manuscript to a special series or article collection?	No
**Experimental design and statistics**<br><br><br>Full details of the experimental design and statistical methods used should be given in the Methods section, as detailed in our Minimum Standards Reporting Checklist. Information essential to interpreting the data presented should be made available	Yes

in the figure legends.<br><br>Have you included all the information requested in your manuscript?	
**Resources**<br><br>A description of all resources used, including antibodies, cell lines, animals and software tools, with enough information to allow them to be uniquely identified, should be included in the Methods section. Authors are strongly encouraged to cite Research Resource Identifiers (RRIDs) for antibodies, model organisms and tools, where possible.<br><br>Have you included the information requested as detailed in our Minimum Standards Reporting Checklist?	Yes
**Availability of data and materials**<br><br>All datasets and code on which the conclusions of the paper rely must be either included in your submission or deposited in publicly available repositories (where available and ethically appropriate), referencing such data using a unique identifier in the references and in the "Availability of Data and Materials" section of your manuscript.<br><br>Have you have met the above requirement as detailed in our Minimum Standards Reporting Checklist?	Yes

Powered by Editorial Manager® and ProduXion Manager® from Aries Systems Corporation

Manuscript

Click here to view linked References

Click here to access/download;Manuscript;OmicsSIMLA_paper_final_clean.

A multi-omics data simulator for complex disease studies and its application to evaluate multi-omics data analysis methods for disease classification

Ren-Hua Chung[1*], Chen-Yu Kang[1]

[1]Division of Biostatistics and Bioinformatics, Institute of Population Health Sciences, National Health Research Institutes, Zhunan, Taiwan

*Corresponding author: Ren-Hua Chung, PhD

Address: No 35, Keyan Road, Zhunan, Miaoli, 350, Taiwan

Tel: 886-37-246-166 #36105

Fax: 886-37-586-467

Email: rchung@nhri.org.tw

// Abstract

Background

An integrative multi-omics analysis approach that combines multiple types of omics data including genomics, epigenomics, transcriptomics, proteomics, metabolomics, and microbiomics, has become increasing popular for understanding the pathophysiology of complex diseases. Although many multi-omics analysis methods have been developed for complex disease studies, only a few simulation tools that simulate multiple types of omics data and models their relationships with disease status are available and these tools have their limitations in simulating the multi-omics data.

Results

We developed a multi-omics data simulator OmicsSIMLA, which simulates genomics (i.e., SNPs and copy number variations (CNVs)), epigenomics (i.e., bisulphite sequencing), transcriptomics (i.e., RNA-seq), and proteomics (i.e., normalized reverse phase protein array) data at the whole-genome level. Furthermore, the relationships between different types of omics data, such as meQTLs (SNPs influencing methylation), eQTLs (SNPs influencing gene expression), and eQTM (methylation influencing gene expression), were modeled. More importantly, the relationships between these multi-omics data and the disease status were modeled as well. We used OmicsSIMLA to simulate a multi-omics dataset for breast cancer under a hypothetical disease model, and used the data to compare the performance among

existing multi-omics analysis methods in terms of disease classification accuracy and runtime. We also used OmicsSIMLA to simulate a multi-omics dataset with a scale similar to an ovarian cancer multi-omics dataset. The neural network-based multi-omics analysis method, ATHENA, was applied to both the real and simulated data and the results were compared.

Conclusions

Our results demonstrated that complex disease mechanisms can be simulated by OmicsSIMLA, and ATHENA showed the highest prediction accuracy when the effects of multi-omics features (e.g., SNPs, CNVs, and gene expression levels) on the disease were strong. Furthermore, similar results can be obtained from ATHENA when analyzing the simulated and real ovarian multi-omics data.

Keywords

Multi-omics data, complex disease study, simulation tool

Introduction

Complex diseases such as hypertension, type 2 diabetes, and autism are caused by multiple genetic and environmental factors [1]. Genome-wide association studies have identified many genetic variants (i.e., SNPs) associated with the complex diseases. However, it remains

difficult to understand the roles of the associated SNPs in the molecular pathophysiology of the disease and how the SNPs interact with other SNPs in a biological network [2]. With the advancement of high-throughput sequencing technology such as next-generation sequencing (NGS) and massive parallel technology such as mass spectrometry, multiple types of omics data (i.e., multi-omics data) including genomics, epigenomics, transcriptomics, proteomics, metabolomics, and microbiomics are rapidly generated [3]. As a single type of data generally cannot capture the complexity of molecular events causing the disease, an integrative approach to combining the multi-omics data would be ideal to help elucidate the pathophysiology of the disease [2].

Integrative methods to combine multi-omics data for disease studies have been developed rapidly [4-8]. They can be generally classified into two categories: multi-staged and meta-dimensional approaches [9]. The multi-staged approach aims to first identify relationships between the multi-omics data, and then test the associations between the multi-omics data and the phenotype. For example, Jennings et al. [7] constructed a Bayesian hierarchical model consisting of two stages. The first stage partitioned gene expression into factors accounted by methylation, copy number variation (CNV), and other unknown causes. These factors were subsequently used as predictors for clinical outcomes in the second stage model. One advantage of this approach is that the causal relationships between multi-omics data can

be modeled. In contrast, the meta-dimensional approach combines the multi-omics data simultaneously. Raw or the transformed data from the multi-omics data are combined into a single matrix for the analysis. This approach allows for a more flexible inference of the relationships among the multi-omics data, without the assumptions of the causal relationships between these data.

Although many multi-omics analysis methods for disease studies are available, they were generally evaluated by simulations with data generated specifically to the methods. To compare the performance among these methods, it is necessary to use the same simulated multi-omics dataset with disease status. Furthermore, sample size estimation when planning a multi-omics study to ensure sufficient power also becomes important [3]. This also requires a simulation tool that simulates realistic multi-omics data structures and models the architecture of the complex disease. However, current simulation tools for disease studies mainly focused on simulating a certain type of omics data. For example, more than 25 simulators are available for simulating genetic data with phenotypic trait, according to the Genetic Simulation Resources website (https://popmodels.cancercontrol.cancer.gov/gsr/). Tools such as WGBSSuite [10] and pWGBSSimla [11] can simulate whole-genome bisulphite sequencing (WGBS) data in case-control samples. Moreover, tools such as Polyester [12] and SimSeq [13] simulate RNA-seq data with differential gene expression

between two groups of samples.

There are only a few available tools that can simulate multi-omics data and allow relationships among different data types to be modeled. One of them is HIBACHI [14], which provides a prototype to simulate genetic interactions under a biological architecture. The biological framework includes 6 genetic variants: 1 variant in a protein-coding gene that changes an amino acid, 2 variants that are regulatory variants in a promoter and an enhancer, 2 variants that code for transcription factors binding to the promoter and the enhancer, and 1 variant in an miRNA gene involved in post-translational regulation for the protein-coding gene. A mathematical framework is then used in HIBACHI to generate phenotypic values based on the biological framework followed by a liability threshold model to generate the disease status. Hence, HIBACHI simulates genotypes and phenotypes under the complex biological and mathematical models, but it cannot generate other types of omics data. Another tool is InterSIM [15], which simulates methylation rates and normalized gene and protein expression levels based on the ovarian cancer (OV) data from The Cancer Genome Atlas (TCGA) project [16]. The correlations within and between each data type are also modeled based on the correlation structures observed in the OV data. A more recently developed tool is MOSim [17], which simulates more multi-omics data types, including RNA-seq, ATAC-seq, ChIP-seq, miRNA-seq, and WGBS data. In contrast to InterSIM, the

regulatory relationships (i.e., activation and repression effects) between the gene expression and other types of data (e.g., CpGs, transcription factors, and miRNAs) are more specifically modeled in MOSim. However, genomics data, the relationships between genomics data and other types of omics data, and the relationships between genomics data and the disease status are not simulated and modeled by InterSIM and MOSim.

Here, we developed the multi-omics data simulator OmicsSIMLA, which simulates genomics data including SNPs and CNVs, epigenomics data such as the WGBS data, transcriptomics data (i.e., RNA-seq), and proteomics data such as the normalized reverse phase protein array (RPPA) data at a whole-genome level. Furthermore, the relationships between different types of omics data, such as meQTLs (SNPs influencing methylation), eQTLs (SNPs influencing gene expression), and eQTM (methylation influencing gene expression), were specifically modeled. More importantly, the relationships between these multi-omics data and disease status were modeled as well. The disease models in OmicsSIMLA are flexible so that the main effects and/or interaction effects (either risk or protective) of SNPs and CNVs on the disease can be specified. Differential methylation and differential gene and protein expression between cases and controls can also be simulated. We demonstrated the usefulness of OmicsSIMLA by simulating a multi-omics dataset for breast cancer under a hypothetical disease model, and compared the performance among existing multi-omics analysis tools

based on the data. We also simulated a multi-omics dataset with a scale similar to the TCGA OV data (except for the methylation data where a smaller set of CpGs was simulated), and the effects of the multi-omics data on the phenotype were modeled based on the parameters estimated from the real OV data. We then compared the results from a multi-omics analysis method when applied to both the real and simulated OV data.

Results

Figure 1 shows the framework of OmicsSIMLA. The genomics data that can be simulated include SNPs and CNVs. Genotypes at SNPs in unrelated and/or family samples are simulated based on the SeqSIMLA2 algorithm [18]. CNV status (i.e., a deletion, normal, one duplication or two duplications) on a chromosome is simulated based on the user-specified chromosomal regions and CNV frequencies. Affection status of each sample is determined by a logistic penetrance function conditional on the causal SNPs and CNVs, and/or the interactions among the causal SNPs. The epigenomics data are the methylated and total read counts at CpGs based on bisulphite sequencing, simulated using the pWGBSSimla algorithm incorporating methylation profiles for 29 human cell and tissue types [11]. Allele-specific methylation (ASM), in which paternal and maternal alleles have different methylation rates, and differentially methylated region (DMR), where the same CpGs in the region have different methylation rates among different cell types, can also be simulated. Furthermore, the

transcriptomics data (i.e., RNA-seq read counts) are simulated with a parametric model assuming a negative-binomial distribution. Finally, the mass-action kinetic action model [19] is used to simulate proteomics data at a certain time point incorporating the gene expression data. Some SNPs can be specified as meQTLs and eQTLs, and some CpGs can be specified as eQTM. Allele-specific expression (ASE), which alleles in a gene have different expression levels, caused by cis-eQTL can also be simulated. The differential methylation, gene expression, and protein expression levels between cases and controls are simulated conditional on the affection status.

To simulate the SNP data, an external reference sequences (i.e., haplotypes) generated by an external sequence generator, such as COSI [20] or HAPGEN2 [21], are required. Using HAPGEN2, we compiled several reference sequence files for the African, Asian, and European populations similar to the linkage disequilibrium (LD) structures and allele frequencies of the variants on chromosome 1 in the 1000 Genomes Project data [22]. For the CNV simulations, a CNV information file, which contains the CNV types (i.e., deletion, normal, or duplication), CNV frequencies, and odds ratios (OR) of the CNVs for the disease, is required. We compiled CNV profiles based on the frequencies of 2,884 focal CNVs observed in the TCGA data for 33 cancers. The optional files for simulating the genomics data include a recombination file, which specifies the recombination rates among SNPs; a

pedigree file, which specifies the pedigree structures if family data are simulated; and a proband file, which specifies the affection status of the family members. For the WGBS simulations, we compiled profiles of 29 human cell and tissue types such as liver, kidney, and colon [11]. We also compiled profiles of normal and tumor tissue types for 31 cancers to simulate the RNA-seq data. Finally, we compiled profiles of tumor tissues for 26 cancers to simulate the protein expression data. Therefore, the user can easily specify the tissue types and the numbers of samples to simulate the CNV, WGBS, RNA-seq, and protein expression data. The formats of the profiles are clearly described in the OmicsSIMLA user manual so that the user can alternatively compile profiles based on his/her own data. Further details on how the profiles are compiled and how OmicsSIMLA generates data based on the profiles are described in the Methods section.

Several options are available in OmicsSIMLA to flexibly model the relationships between the multi-omics data and the disease. For example, the ORs for the main effects and pairwise interaction effects of selected SNPs can be specified. An additive, dominant, or recessive model can be assumed for the main effects, and several interaction models can be specified, as described in the Methods section. The user can also specify the proportions of methylated (i.e., methylation rates > 70%), unmethylated (i.e., methylation rates < 30%), and partially methylated (i.e., methylation rates between 30 and 70%) CpGs that have different

methylation rates between cases and controls, and the difference in the methylation rates can also be specified. The fold changes of the gene expression levels for the differentially expressed genes between cases and controls can also be specified. Several options are also available to model the relationships between the multi-omics data. For example, the fold changes of the methylation rates influenced by meQTLs can be specified. Similarly, fold changes of the gene expression levels influenced by eQTLs and eQTMs can be specified. A user-friendly web interface is provided at https://omicssimla.sourceforge.io/generateCommand.html to conveniently specify the abovementioned parameters. All of the input files and parameters that are required and optional to OmicsSIMLA are clearly described on the web interface.

Using OmicsSIMLA, we simulated a multi-omics dataset based on hypothetical pathways for breast cancer as described in Ritchie et al. [9] and illustrated in Figure 2. The data included a deletion with a protective effect in the CYP1A1 gene, 3 common SNPs with risk effects in the CYP1B1 gene, 5 rare SNPs in the COMT gene, which had interaction effects with a meQTL for the XRCC1 gene, and 5 rare SNPs in the GSTM1 gene, which also had interaction effects with an eQTL affecting the gene and protein expression of the XRCC3 gene. Finally, 5 rare SNPs in the GSTT1 gene also had interaction effects with a SNP in a regulatory region. A total of 2,022 SNPs in the four genes (i.e., CYP1B1, COMT, GSTM1,

and GSTT1) and a regulatory region consisting of the meQTL, eQTL, and the SNP interacting with GSTT1, 1 CNV in CYP1A1, 688 CpGs in XRCC1, and gene and protein expression levels for 100 genes (including the expression for XRCC3 and 99 other hypothetical genes in the pathways) were simulated. More details about the simulations can be found in the Methods section.

Based on the simulated datasets, we compared the performance of methods from the two categories of multi-omics analysis methods (i.e., multi-staged and meta-dimensional approaches) for disease prediction by measuring the area under the curve (AUC). The SNPs, CNV, methylation levels at CpGs, and gene and protein expression levels were used as the features for the prediction. The disease status served as the label for classification, and the prediction accuracy was measured based on the numbers of cases and controls that were correctly predicted. For the multi-staged method, we implemented the three-stage method [23]. Briefly, significant SNPs and CNV associated with the disease (i.e., association p-values less than 0.05) were first selected. The significant SNPs and CNV were then tested for associations with each feature in the methylation and gene and protein expression data, and the significant features were selected. Finally, a logistic regression prediction model was constructed based on these significant features. Further details on how the three-stage method was implemented are provided in the Supplementary methods. The meta-dimensional

methods we used included the random forest-based method (RFomics), a graph-based integration method (CANetwork) [5], and a model-based integration method (ATHENA) [4]. The RFomics combines the preprocessed multi-omics data in a single matrix for constructing the prediction model. As described in the Supplementary methods, a gene-based risk score is calculated based on SNPs for each gene. Then the risk scores and other multi-omics data are normalized so that they can be evaluated on the same scale by the random forest (RF) algorithm. In contrast, CANetwork calculates a graph matrix to measure the distance between samples using the composite association network algorithm [24], and the prediction model is created based on the distance matrix using the graph-based semi-supervised learning algorithm [25]. Finally, ATHENA uses Grammatical Evolution Neural Networks (GENN), which optimize artificial neural networks based on genetic programming, to construct a meta-dimensional model from multi-omics data for prediction. The parameters for RFomics and ATHENA used in our simulations are shown in Supplementary Table S1.

Table 1 shows the area under the curve (AUC) for the four methods under three scenarios. Scenario 1 had 500 cases and 500 controls in the training set, and 100 cases and 100 controls in the validation set. Scenario 2 had the same sample sizes as those in Scenario 1, but the multi-omics data had less strong effects on the disease compared to Scenario 1. The effects of the multi-omics data were the same in Scenarios 3 as those in Scenario 1, but Scenarios 3 had

larger sample size (i.e., 1,500 cases and 1,500 controls in the training data and 500 cases and 500 controls in the validation data). More details of the three scenarios are provided in the Methods section. Prediction models for the four methods were created based on the training data, and their prediction accuracies were evaluated by the validation data. In Scenarios 1 and 3, ATHENA had a significantly higher AUC than the other three methods. The three-stage method had an AUC similar to RFomics, and CANetwork had the lowest AUC. In Scenario 2 where the effects were less strong on the disease, the four methods had similar AUCs. Table 2 shows the runtime for the three methods. In Scenario 1, RFomics and CANetwork had similar performance, whereas ATHENA had more than 500-times the runtime of RFomics and CANetwork. In Scenario 3, CANetwork was the most efficient method followed by the RFomics and the three-stage method, and ATHENA had a significantly longer runtime than the other three methods.

We also used OmicsSIMLA to simulate a multi-omics dataset based on the TCGA OV data. The TCGA OV data included focal CNV, methylation, RNA-seq, and RPPA data in 66 individuals with short-term survival (i.e., survival time of less than 3 years) and 107 individuals with long-term survival (i.e., survival time of more than 3 years). Further details of the OV data are provided in the Methods section. Our simulation results showed that ATHENA had a higher AUC than the other three multi-omics analysis methods; hence, we

applied ATHENA to the TCGA OV data several times with different random seeds to identify a GENN model that can classify the short-term and long-term survivals with the highest AUC. As shown in Figure 3, the best GENN model constructed by ATHENA (having an AUC of 0.826) comprised five features, including methylation of a CpG at the KIF13B gene and gene expression levels at the LRRN4, MARCH9, LRIG1, and TCEAL8 genes. By examining the correlation structures in the five features, we hypothesized that the correlations among the gene expression levels of MARCH9, LRIG1, and TCEAL8 were caused by the CpG at KIF13B (i.e., an eQTM), and the gene expression of LRRN4 exhibited an independent effect on the survival time. We then used OmicsSIMLA to simulate similar numbers of focal CNVs and genes with RNA-seq and RPPA data as those in the OV data. We also simulated methylation levels at CpGs on chromosome 1. A CpG with differential methylation was specified as an eQTM which affected the gene expression of three genes. The three genes were also differentially expressed. Furthermore, an independent gene was specified to be differentially expressed as well. The model for the survival time is illustrated in Figure 4. A total of 50 replicates of 500 cases (i.e., the short-term survival group) and 500 controls (i.e., the long-term survival group) were simulated. Each replicate included 2,884 focal CNVs, 2,753 CpGs, gene expression levels for 12,004 genes, and protein expression levels for 200 genes. Finally, ATHENA was applied to the 50 replicates. We found that up to three of the five causal features can be selected in the same model by ATHENA in the 50 replicates. The

average AUC calculated from 5-fold cross-validation in ATHENA over the 50 replicates was 0.757, which was comparable to the AUC of 0.826 calculated from the real data. Figure 5 shows a GENN model from 1 of the 50 replicates that connected the features with a PDIV node similar to the one in Figure 3. The results demonstrated that OmicsSIMLA can simulate multi-omics data with a scale similar to the real dataset and incorporate a model with similar effects on the survival time.

Discussion

We have developed OmicsSIMLA, which simulates multi-omics data (i.e., genomics, epigenomics, transcriptomics, and proteomics data) with disease status. OmicsSIMLA simulates multiple types of omics data while the relationships between different types of omics data and the relationships between the omics data and the disease are modeled. As the development of integrative methods for analyzing multi-omics data has attracted substantial interest from researchers, OmicsSIMLA will be very useful to simulate benchmark datasets for comparisons of these methods. Furthermore, as more and more disease studies take advantages of multi-omics data, OmicsSIMLA will also be very useful for power calculations and sample size estimations when planning a new study.

Performing simulation studies using OmicsSIMLA has several advantages and disadvantages

as compared to using HIBACHI, InterSIM, and MOSim. For example, the mathematical framework in HIBACHI for the 6 genetic variants and an environmental factor allows the user to generate complicated genetic models, including high-order gene-gene interactions, but only pairwise gene-gene interactions are modeled in OmicsSIMLA. However, an advantage of OmicsSIMLA over HIBACHI is that not only genomics data and disease status but also other types of omics data can be simulated. On the other hand, InterSIM models the correlations within and among the methylation, gene expression, and protein expression data based on correlation structures observed in the TCGA OV data. OmicsSIMLA can model the correlations of methylation levels at local CpGs [11]. The gene and protein expression levels for different genes are generally independently simulated in OmicsSIMLA. The correlations of gene and protein expression levels between different genes can be modeled by a common regulatory variant, such as an eQTL or an eQTM in OmicsSIMLA. By contrast, MOSim is more flexible in simulating different experimental designs, such as different numbers of experimental groups, data at different time points, and different numbers of replicates under an experimental condition. However, InterSIM and MOSim do not simulate genomics data; hence, eQTLs and meQTLs cannot be generated. From the above discussion, we emphasized that the four simulators have their own capabilities, advantages, and disadvantages; hence, the choice of a proper simulator must be based on the purpose of the study performed.

We used OmicsSIMLA to simulate a multi-omics dataset for breast cancer based on hypothetical pathways. Four analysis tools were compared using the dataset. The results showed that the neural network-based method ATHENA achieved the highest AUC when the effects of the multi-omics features on the disease were strong. However, the AUCs of the four methods were similar when the effects were modest. Furthermore, RFomics and CANetwork had comparable runtimes, and ATHENA was the most computationally expensive approach. In practice, ATHENA is the ideal tool to perform multi-omics data analysis because of its high AUC if the executing time is acceptable.

We also used OmicsSIMLA to simulate a multi-omics dataset with a scale similar to the TCGA OV data. The effects of the multi-omics features on the phenotype were estimated and modeled based on the OV data. As shown in the simulations of the breast cancer pathways and the simulations based on the OV data, three main components are generally required to perform the simulations in OmicsSIMLA. These include a biological model (i.e., the disease model), reference sequences and profiles, and parameter values to model the effects of the multi-omics features on the disease and to model the relationships between multi-omics data. The biological model is usually hypothesized based on the literature results and observations in real data. Profiles such as the CNV, methylation, gene expression, and protein expression profiles are then required to simulate the multi-omics data. Since we have compiled profiles

for many tissue types, the user can conveniently choose the profiles to perform the simulations. Note that the profiles were compiled using the tumor tissue data from the TCGA project, except for the methylation profiles and some of the gene expression profiles. If multi-omics data for other tissue types are available, the user can also compile the profiles based on the data following the instructions in the user manual. To simulate genomics data, we compiled several sets of reference sequences for the African, Asian, and European populations based on allele frequencies and LD structures in human sequences. Finally, several parameters are needed in OmicsSIMLA to specify the disease model and to model the relationships among the multi-omics data; recommended values of some of these parameters are provided in the user manual. For example, the ORs of common SNPs (i.e., minor allele frequencies (MAFs) $\geq$ 5%) for complex diseases were generally observed to be between 0.5 and 2 according to the GWAS catalog [26], and the ORs of rare SNPs (MAFs < 5%) can be a function of the MAFs [27]. Note that, performing the simulations using the pre-compiled profiles, reference sequences, and recommended parameter values is simplified, but the user can still opt to customize the profiles and reference sequences with specific parameter values to flexibly perform the simulations.

Currently, OmicsSIMLA focuses on simulating the dichotomous trait (i.e., affection status). As studies for quantitative traits are also important, it is our future work to extend

OmicsSIMLA to simulate quantitative traits based on the classic quantitative genetics model [28]. Furthermore, environmental factors and the interactions between genes and environments can also play important roles in complex disease etiology. Therefore, simulating exposome data such as the climate and air quality data and modeling their interactions with genes are also important in the future extensions of OmicsSIMLA.

Conclusions

In conclusion, we developed a useful multi-omics data simulator, OmicsSIMLA, for complex disease studies. Benchmark datasets can be simulated by OmicsSIMLA for evaluating different multi-omics data analysis methods for disease studies. OmicsSIMLA can also be used to estimate sample sizes and statistical power when designing a new multi-omics disease study. OmicsSIMLA is freely available at https://omicssimla.sourceforge.io.

Methods

Simulation of DNA sequences

The SeqSIMLA2 package [18] is integrated in OmicsSIMLA to generate DNA sequences in unrelated/related individuals. Similar to SeqSIMLA2, OmicsSIMLA expects a set of external reference sequences (i.e., haplotypes) generated by an external sequence generator, such as COSI [20] or HAPGEN2 [21] that has been widely adopted in genetics studies. Generally, a

set of 10,000 or more reference sequences are expected. Optional files consisting of recombination rate information and pedigree structures are also accepted. A gene dropping algorithm assuming random mating with crossovers is performed based on the reference sequences, recombination rates, and pedigree structures to generate haplotypes in each individual.

Simulation of CNVs

For the simulation of CNVs, we considered four CNV states including deletion (D), normal (N), one duplication (U), and two duplications (UU) on a chromosome. Therefore, there are 10 types of CNV states on the two chromosomes in an individual, as shown in Supplementary Table S2, and the total copy numbers on the two chromosomes range from 0 to 6. During meiosis, we use the single-copy crossover model, assuming all crossovers occurred between CNVs [29]. We compiled profiles of CNV frequencies of D and U for 2,884 focal CNVs observed in the TCGA data for 33 cancers. Further details on how the CNV profiles were generated are provided in the Supplementary methods. Alternatively, the user can provide frequencies and ranges of the four CNV states for different CNV regions.

Simulation of affection status

Genetic variants, including SNPs and CNVs, are used to determine the affection status of an individual based on a logistic penetrance function as follows:

$$\mathrm{logit}(P(affected)) = \beta_0 + \sum_{i\in\Omega} \beta_{C_{i1}} C_{i1} + \sum_{i\in\Omega} \beta_{C_{i2}} C_{i2} + \sum_{j\in\Psi} \beta_{G_j} G_j + \sum_{m,n\in\Upsilon} \beta_{mn} G_{mn}$$

where $P(affected)$ is the probability of being affected, β_0 determines the baseline prevalence, Ω, Ψ, and Υ are sets of causal CNVs, SNPs with main effects, and SNPs with interaction effects, respectively, specified by the user, C_{i1} and C_{i2} are the CNV states for the first and second haplotypes at CNV *i*, respectively, G_j is the genotype coding at SNP *j*, and G_{mn} is the genotype coding at SNPs *m* and *n*. C_{i1} *and* C_{i2} have values of -1, 0, 1, and 2 for CNV states *D*, *N*, *U*, and *UU*, respectively, where *N* is the baseline state. The coding of G_j is based on a dominant, additive or recessive model, and the coding of G_{mn} is based on several interaction models. If SNP *j* is in a CNV region, allelic CNV [30] is considered in the coding of G_j. More details of the coding of G_j and G_{mn} are provided in the Supplementary methods. The parameters β_C and β_G are the effect sizes of the main effects for CNVs and SNPs, respectively, and β_{mn} determines the effect size of the interaction effect between SNPs *m* and *n*. These parameters are specified by the user.

Simulation of DNA methylation data

The pWGBSSimla package [11] is integrated into OmicsSIMLA to generate the WGBS data. The pWGBSSimla algorithm simulates data using methylation profiles generated based on 41 WGBS datasets for 29 human cell and tissue types. The profiles contain the information for each CpG, such as its distance to the next site, methylation rate, methylation status (i.e., methylated, unmethylated, and fuzzily methylated), and read counts for each type of

methylation status. CpGs and the distances between the CpGs are first determined based on the profiles, and then the total read count and methylated read count are simulated for each CpG. Methylation level at a CpG influenced by a meQTL is simulated based on a genotype-specific methylation probability, which is the methylation rate of the CpG in the profiles multiplied by a user-specified ratio. Furthermore, ASMs are simulated based on father- and mother-specific methylation rates for paternal and maternal alleles, respectively. Finally, a DMR is generated by simulating the same genomic region using profiles for different cell or tissue types. OmicsSIMLA currently simulates methylation data for CpGs on the same chromosome per run. Multiple runs of OmicsSIMLA can be executed to simulate CpGs on different chromosomes. More details of the pWGBSSimla algorithm can be found in Chung and Kang [11].

Simulation of RNA-seq data

We implemented a parametric simulation procedure for simulating the RNA-seq data similar to that described in Benidt and Nettleton [13]. A negative binomial (NB) distribution with mean μ_{ij} and dispersion parameter ω_i is used to simulate the read count for gene *i* in individual *j*. The mean is calculated as $\mu_{ij} = \lambda_i c_j$, where λ_i is the common mean for gene *i* and c_j is the individual-specific normalization factor for individual *j*. The individual-specific normalization factor is used to model systematic variations among individuals due to technical variation [31]. We compiled RNA-seq

profiles for normal and tumor tissues of 31 cancers based on the TCGA whole genome RNA-seq data. For each tissue type, the profiles comprised a vector $\boldsymbol{c}$ of individual-specific normalization factors calculated based on the TCGA samples and vectors $\boldsymbol{\lambda}$ and $\boldsymbol{\omega}$ for genes across the genome. Further details on how the RNA-seq profiles were generated are provided in the Supplementary methods. Note that when there are no technical variations to simulate, the user can replace the vector $\boldsymbol{c}$ with a vector of $\boldsymbol{1}$, where the length of the vector is the number of samples to be simulated. The parameters λ_i and ω_i are then randomly sampled with replacement from $\boldsymbol{\lambda}$ and $\boldsymbol{\omega}$. If more samples than those in the TCGA data are simulated, we use the smoothed bootstrap procedure [32] to calculate c_j^* for individual *j*, and μ_{ij} is calculated as $\lambda_i c_j^*$. More details of the calculation of c_j^* are also provided in the Supplementary methods. The user can specify *n* differentially expressed (DE) genes between cases and controls and their fold changes, and the read count for DE gene *i* in individual *j* is simulated based on a NB distribution with mean $f_i \mu_{ij}$ and dispersion parameter ω_i, where f_i is the fold change for gene *i*.

<u>Simulation of eQTL and allele-specific reads</u>

We followed the procedure in the simulation study in Sun [33] to simulate eQTL and read counts for ASE. For eQTL *l* with a user-specified fold change h_l, the means for the three genotypes *AA*, *Aa*, and *aa* at the eQTL are μ_{ij}, $h_l \mu_{ij}$, and $(2h_l - 1)\mu_{ij}$, respectively, and the

dispersion parameter is ω_i in the NB distribution for gene *i* influenced by the eQTL. ASE for a gene caused by a cis-eQTL is simulated by assuming reads were mapped to heterozygous SNPs (i.e., allele-specific reads) in the gene. A cis-eQTL refers to the eQTL being located in the cis-regulatory elements of the gene. Because the alleles at the cis-eQTL can be in the same haplotype as the alleles of the gene, ASE can be observed using the allele-specific reads of the gene. Furthermore, only heterozygous SNPs can be tested for cis-eQTL with the allele-specific reads. Therefore, we simulate allele-specific reads for heterozygous eQTLs. Assuming t_{ij} is the total read count for gene *i* in individual *j*, the total number of allele-specific reads is calculated as $0.005t_{ij}$, where 0.005 was estimated from real data by Sun [33]. Furthermore, also suggested by Sun [33], the number of allele-specific reads for a haplotype is simulated using a beta-binomial distribution with a mean determined by the effect size of the cis-eQTL and an overdispersion parameter of 0.1. The effect size is defined as $\log_2$(expression of the alternative allele at the eQTL/expression of the reference allele at the eQTL) [34] for a heterozygous cis-eQTL and is set to 0 for a homozygous cis-eQTL.

Simulation of eQTM

We used linear regression to model the relationship between gene expression and methylation:

$\mu_i^{'} = E(y_{ij}) = \alpha_i + \beta_i x_{ij}$, where y_{ij} and x_{ij} are the RNA-seq read count and the proportion of methylated reads, respectively, for gene *i* influenced by methylation in individual *j*. Assuming

that the NB parameters for gene *i* are μ_i and ϕ_i, the parameter α_i is specified as μ_i, and β_i is assumed to follow a normal distribution with a mean and a standard deviation specified by the user. Then the gene expression of gene *i* is simulated by an NB distribution with parameters of $\mu_i^{'}$ and ϕ_i.

Protein expression simulation

We assumed that the protein expression level for protein *k* at a time point *t* in sample *j* follows a normal distribution with a mean η_{kjt} and a standard deviation τ_k after normalization. We used the mass-action kinetic action model [19] to simulate protein expression at a certain time point. The mean $\eta_{kj,t+1}$ for the protein expression at time *t*+1 was determined as follows:

$$\eta_{kj,t+1} = \eta_{kjt} + (x_{kjt}\kappa_{jt}^{s} - \eta_{kjt}\kappa_{jt}^{d}),$$

where x_{kjt} is the normalized gene expression for the gene encoding protein *k*, and κ_{jt}^{s} and κ_{jt}^{d} are the protein synthesis and degradation rates, respectively, in individual *j* at time *t*. The normalized gene expression x_{kjt} is calculated using the median absolute deviation (MAD) scale normalization [35] based on the RNA-seq data simulated from the previous section. Similar to the simulation study in Teo et al. [19], κ_{jt}^{d} is fixed to be 1, and κ_{jt}^{s} with a default value of 1 can be changed by the user. We compiled protein expression profiles consisting of a vector of standard deviations $\boldsymbol{\tau}$ for 26 cancers from the TCGA project. The standard deviations were estimated from the level 4 protein expression data of each tissue type with

more than 50 patients. The level 4 data consisted of protein expression data that have been normalized across the samples as well as across the proteins, and a replication-based method was used to account for differences in protein expression among different batches. More details about the generation of the profiles are provided in the Supplementary methods. The parameter τ_j is then randomly sampled with replacement from $\boldsymbol{\tau}$.

Simulation studies based on the hypothetical breast cancer disease model

We used OmicsSIMLA to evaluate the performance of the three-staged method, RFomics, CANetwork and ATHENA. A hypothetical disease model for breast cancer involving multi-omics data [9] was simulated, as shown in Figure 2. To be more specific, a deletion with a frequency of 20%, which had a protective effect with an odds ratio (OR) of 0.67, in the CYP1A1 gene and 3 common variants, which had main effects (ORs = 1.5) with minor allele frequencies (MAFs) > 10%, in the CYP1B1 gene were simulated. We also simulated 5 rare variants with MAFs < 3% in the COMT gene, which had interaction effects (ORs = 5) with a meQTL for the XRCC1 gene. The CpG in XRCC1 influenced by the meQTL caused a difference in methylation rates of 10% between cases and controls. Furthermore, we simulated 5 rare variants in the GSTM1 gene, which had interaction effects (ORs = 5) with a cis-eQTL for the XRCC3 gene, and 5 rare variants in the GSTT1 gene, which had interaction effects (ORs = 5) with a SNP located in the same region as that of the meQTL and eQTL. The eQTL caused a fold change of 1.5 in the XRCC3 gene expression compared to the reference

genotype, and a fold change of 1.5 was simulated for the differential gene expression of XRCC3 between cases and controls. In summary, the total variables consisted of 200, 687, 264, and 176 SNPs in the CYP1B1, COMT, GSTM1, and GSTT1 genes, respectively, and 695 SNPs harboring the meQTL, eQTL, and the SNP interacting with GSTT1 in the regulatory region, a variable for CNV status in CYP1A1, methylation levels at 688 CpGs in XRCC1, and gene and protein expression levels for 100 genes and their encoded proteins. More details for generating the reference sequences in the genes and the simulations for each omics data type are provided in the Supplementary methods.

We simulated a training dataset consisting of 500 cases and 500 controls as well as a validation dataset consisting of 100 cases and 100 controls. The training dataset was used by the three-staged method, RFomics, CANetwork, or ATHENA to construct a prediction model. The validation dataset was then used to calculate the AUC based on the prediction model. Note that a 5-fold cross-validation was performed in ATHENA, and a best model based on the testing dataset (i.e., one of the five random 20% of the training dataset) was created for each cross-validation. The model with the highest AUC based on the testing dataset was selected and applied to the validation dataset. This simulation scenario was referred to as Scenario 1. We also simulated a scenario with less strong genetic effects (Scenario 2) and a scenario with larger sample size (Scenario 3). More details about Scenarios 2 and 3 are

provided in the Supplementary methods. For each scenario, 100 batches of training and validation datasets were simulated, and the AUC for each algorithm was averaged over the 100 batches.

Simulation studies based on the TCGA OV data

We also used OmicsSIMLA to simulate a multi-omics dataset based on the OV data from the TCGA project. The data were downloaded using RTCGAToolbox [36], an R package that allows the retrieval of the TCGA pre-processed data from the Firehose pipeline (http://gdac.broadinstitute.org). A total of 173 tumor samples with clinical (i.e., the survival time), CNV, methylation, RNA-seq, and RPPA data available were extracted. Same as the definition used by Kim et al. [37], patients with a survival time shorter than 3 years were referred to as short-term survival, whereas patients with a survival time longer than 3 years were referred to as long-term survival. The CNV data consisted of the discrete CNV statuses at focal CNVs based on GISTIC2 [38] calls in 2,884 genes. The methylation data had methylation rates at 25,794 CpG sites in 13,157 genes. The RNA-seq data comprised the RNA-Seq by Expectation Maximization (RSEM) [39] counts of 17,946 genes, and the RPPA data comprised the normalized protein expression data of 204 genes.

Each of the features in each type of omics data was first tested for association with the survival time (i.e., short-term and long-term survival) by fitting a logistic regression model.

For each type of omics data, the 50 most significant features sorted via the association p-values were used in ATHENA. The parameter values shown in Supplementary Table S1 were specified in ATHENA. We then examined the pairwise correlations among the five features identified by the best model shown in Figure 3 from ATHENA. We found that the gene expression of LRRN4 had low Pearson correlation coefficients with the other four features. However, there were significant correlations between the methylation level of KIF13B and the gene expression levels of MARCH9 and TCEAL8. There were also significant correlations among the gene expression levels of MARCH9, LRIG1, and TCEAL8. The Pearson correlation coefficients among the five features are shown in Supplementary Table S3. To establish the correlations between methylation and gene expression features as well as the correlations among gene expression features using OmicsSIMLA, we hypothesized that the methylation of KIF13B was an eQTM, which affected the gene expression levels of MARCH9, LRIG1, and TCEAL8. We selected a CpG with a similar methylation rate (i.e., 2%) to that in KIF13B. The CpG was assumed to have a different methylation rate (i.e., 2.3%) in the simulated long-term survival group compared to the 2% rate in the short-term survival group. The rates were similar to those observed in the OV data. Furthermore, the parameter β, which was used to model the relationship between the methylation of the eQTM and gene expression levels of three genes, was assumed to have a mean of 50 and a standard deviation of 20. The parameters were estimated from the OV data. The fold changes of the

three genes influenced by the eQTM were all specified to be 0.6 in the short-term survival group relative to the long-term survival group. Finally, the fold change of the independent gene expression was specified to be 2 in the short-term survival group relative to the long-term survival group. The fold changes were also similar to those observed in the OV data. The simulations were performed based on the CNV, methylation, gene expression, and protein expression profiles compiled according to the TCGA OV data. Further details on how the profiles were generated are provided in the Supplementary methods. A total of 50 batches of data were simulated, with each having 2,884 focal CNVs, 2,753 CpGs on chromosome 1, gene expression levels for 12,004 genes, and protein expression levels for 200 genes in 500 cases (i.e., the short-term survival group) and 500 controls (i.e., the long-term survival group).

Availability of supporting source code and requirements

Project name: OmicsSIMLA

Project home page: https://omicssimla.sourceforge.io

Operating system: Linux

Programming language: C++

Other requirements: C++11 compiler and Eigen and boost libraries if directly compiling the source code.

License: GPL-3.0

RRID: SCR_017011

Availability of supporting data

The simulated datasets supporting the conclusions of this article are available from the OmicsSIMLA website (https://omicssimla.sourceforge.io/download.html).

Declarations

Funding

This work has been supported by a grant from the Ministry of Science and Technology (MOST 106-2221-E-400-005-MY3) in Taiwan.

Authors' contributions

RHC and CYK both designed the framework of the simulation tool and implemented the software. RHC designed the simulation study and CYK performed the simulation analysis. Both authors read and approved the final manuscript.

Competing interests

The authors declare that they have no competing interests.

References

1. Timpson NJ, Greenwood CMT, Soranzo N, Lawson DJ and Richards JB. Genetic architecture: the shape of the genetic contribution to human traits and disease. Nature reviews Genetics. 2018;19 2:110-24. doi:10.1038/nrg.2017.101.
2. Karczewski KJ and Snyder MP. Integrative omics for health and disease. Nature reviews Genetics. 2018;19 5:299-310. doi:10.1038/nrg.2018.4.
3. Hasin Y, Seldin M and Lusis A. Multi-omics approaches to disease. Genome biology. 2017;18 1:83. doi:10.1186/s13059-017-1215-1.
4. Holzinger ER, Dudek SM, Frase AT, Pendergrass SA and Ritchie MD. ATHENA: the analysis tool for heritable and environmental network associations. Bioinformatics. 2014;30 5:698-705. doi:10.1093/bioinformatics/btt572.
5. Yan KK, Zhao H and Pang H. A comparison of graph- and kernel-based -omics data integration algorithms for classifying complex traits. BMC bioinformatics. 2017;18 1:539. doi:10.1186/s12859-017-1982-4.
6. Ruffalo M, Koyuturk M and Sharan R. Network-Based Integration of Disparate Omic Data To Identify "Silent Players" in Cancer. PLoS computational biology. 2015;11 12:e1004595. doi:10.1371/journal.pcbi.1004595.
7. Jennings EM, Morris JS, Carroll RJ, Manyam GC and Baladandayuthapani V. Bayesian methods for expression-based integration of various types of genomics data. EURASIP J Bioinform Syst Biol. 2013;2013 1:13. doi:10.1186/1687-4153-2013-13.
8. Tyekucheva S, Marchionni L, Karchin R and Parmigiani G. Integrating diverse genomic data using gene sets. Genome biology. 2011;12 10:R105. doi:10.1186/gb-2011-12-10-r105.
9. Ritchie MD, Holzinger ER, Li R, Pendergrass SA and Kim D. Methods of integrating data to uncover genotype-phenotype interactions. Nature reviews Genetics. 2015;16 2:85-97. doi:10.1038/nrg3868.
10. Rackham OJ, Dellaportas P, Petretto E and Bottolo L. WGBSSuite: simulating whole-genome bisulphite sequencing data and benchmarking differential DNA methylation analysis tools. Bioinformatics. 2015;31 14:2371-3. doi:10.1093/bioinformatics/btv114.
11. Chung R-H and Kang C-Y. pWGBSSimla: a profile-based whole-genome bisulphite sequencing data simulator incorporating methylation QTLs, allele-specific methylations and differentially methylated regions. bioRxiv. 2018; doi:10.1101/390633.
12. Frazee AC, Jaffe AE, Langmead B and Leek JT. Polyester: simulating RNA-seq datasets with differential transcript expression. Bioinformatics. 2015;31 17:2778-84. doi:10.1093/bioinformatics/btv272.
13. Benidt S and Nettleton D. SimSeq: a nonparametric approach to simulation of RNA-

sequence datasets. Bioinformatics. 2015;31 13:2131-40. doi:10.1093/bioinformatics/btv124.

14. Moore JH, Amos R, Kiralis J and Andrews PC. Heuristic identification of biological architectures for simulating complex hierarchical genetic interactions. Genetic epidemiology. 2015;39 1:25-34. doi:10.1002/gepi.21865.
15. Chalise P, Raghavan R and Fridley BL. InterSIM: Simulation tool for multiple integrative 'omic datasets'. Comput Methods Programs Biomed. 2016;128:69-74. doi:10.1016/j.cmpb.2016.02.011.
16. Cancer Genome Atlas Research N. Comprehensive genomic characterization defines human glioblastoma genes and core pathways. Nature. 2008;455 7216:1061-8. doi:10.1038/nature07385.
17. Martínez-Mira C, Conesa A and Tarazona S. MOSim: Multi-Omics Simulation in R. bioRxiv. 2018:421834. doi:10.1101/421834.
18. Chung RH, Tsai WY, Hsieh CH, Hung KY, Hsiung CA and Hauser ER. SeqSIMLA2: simulating correlated quantitative traits accounting for shared environmental effects in user-specified pedigree structure. Genetic epidemiology. 2015;39 1:20-4. doi:10.1002/gepi.21850.
19. Teo G, Vogel C, Ghosh D, Kim S and Choi H. A Mass-Action-Based Model for Gene Expression Regulation in Dynamic Systems. Cambridge University Press; 2015.
20. Schaffner SF, Foo C, Gabriel S, Reich D, Daly MJ and Altshuler D. Calibrating a coalescent simulation of human genome sequence variation. Genome research. 2005;15 11:1576-83. doi:10.1101/gr.3709305.
21. Su Z, Marchini J and Donnelly P. HAPGEN2: simulation of multiple disease SNPs. Bioinformatics. 2011;27 16:2304-5. doi:10.1093/bioinformatics/btr341.
22. Consortium TGP. A map of human genome variation from population-scale sequencing. Nature. 2010;467 7319:1061-73. doi:10.1038/nature09534.
23. Holzinger ER and Ritchie MD. Integrating heterogeneous high-throughput data for meta-dimensional pharmacogenomics and disease-related studies. Pharmacogenomics. 2012;13 2:213-22. doi:10.2217/pgs.11.145.
24. Mostafavi S, Ray D, Warde-Farley D, Grouios C and Morris Q. GeneMANIA: a real-time multiple association network integration algorithm for predicting gene function. Genome biology. 2008;9 Suppl 1:S4. doi:10.1186/gb-2008-9-s1-s4.
25. Tsuda K, Shin H and Scholkopf B. Fast protein classification with multiple networks. Bioinformatics. 2005;21 Suppl 2:ii59-65. doi:10.1093/bioinformatics/bti1110.
26. Buniello A, MacArthur JAL, Cerezo M, Harris LW, Hayhurst J, Malangone C, et al. The NHGRI-EBI GWAS Catalog of published genome-wide association studies, targeted arrays and summary statistics 2019. Nucleic acids research. 2019;47 D1:D1005-D12. doi:10.1093/nar/gky1120.

27. Wu MC, Lee S, Cai T, Li Y, Boehnke M and Lin X. Rare-variant association testing for sequencing data with the sequence kernel association test. American journal of human genetics. 2011;89 1:82-93. doi:10.1016/j.ajhg.2011.05.029.
28. Falconer DS and Mackay TF. Quantitative genetics. San Francisco: Benjamin Cummings; 1996.
29. Hartasanchez DA, Valles-Codina O, Braso-Vives M and Navarro A. Interplay of interlocus gene conversion and crossover in segmental duplications under a neutral scenario. G3. 2014;4 8:1479-89. doi:10.1534/g3.114.012435.
30. Usher CL and McCarroll SA. Complex and multi-allelic copy number variation in human disease. Briefings in functional genomics. 2015;14 5:329-38. doi:10.1093/bfgp/elv028.
31. McIntyre LM, Lopiano KK, Morse AM, Amin V, Oberg AL, Young LJ, et al. RNA-seq: technical variability and sampling. BMC genomics. 2011;12:293. doi:10.1186/1471-2164-12-293.
32. Efron B and Tibshirani RJ. An Introduction to the Bootstrap. Chapman and Hall/CRC; 1993.
33. Sun W. A statistical framework for eQTL mapping using RNA-seq data. Biometrics. 2012;68 1:1-11. doi:10.1111/j.1541-0420.2011.01654.x.
34. Mohammadi P, Castel SE, Brown AA and Lappalainen T. Quantifying the regulatory effect size of cis-acting genetic variation using allelic fold change. Genome research. 2017;27 11:1872-84. doi:10.1101/gr.216747.116.
35. Fundel K, Kuffner R, Aigner T and Zimmer R. Normalization and gene p-value estimation: issues in microarray data processing. Bioinform Biol Insights. 2008;2:291-305.
36. Samur MK. RTCGAToolbox: a new tool for exporting TCGA Firehose data. PloS one. 2014;9 9:e106397. doi:10.1371/journal.pone.0106397.
37. Kim D, Li R, Dudek SM and Ritchie MD. ATHENA: Identifying interactions between different levels of genomic data associated with cancer clinical outcomes using grammatical evolution neural network. BioData mining. 2013;6 1:23. doi:10.1186/1756-0381-6-23.
38. Mermel CH, Schumacher SE, Hill B, Meyerson ML, Beroukhim R and Getz G. GISTIC2.0 facilitates sensitive and confident localization of the targets of focal somatic copy-number alteration in human cancers. Genome biology. 2011;12 4:R41. doi:10.1186/gb-2011-12-4-r41.
39. Li B and Dewey CN. RSEM: accurate transcript quantification from RNA-Seq data with or without a reference genome. BMC bioinformatics. 2011;12:323. doi:10.1186/1471-2105-12-323.

Figures

Figure 1. Simulation framework of OmicsSIMLA. The black solid lines represent the relationships among different types of omics data. The black dotted lines represent the causal effects of genomics data to the disease. The red dotted lines represent the retrospective simulations of the methylation, gene expression and protein expression levels conditional on the disease status.

Figure 2. Hypothetical pathways involved in breast cancer. The brown solid lines represent the main effects of SNPs and CNVs on the disease, while the green solid lines represent the interaction effects of SNPs on the disease. The black sold lines represent the regulatory effects of the meQTL and eQTL on methylation and gene expression, respectively. The red dotted lines represent the retrospective simulations of the methylation, gene expression and protein expression levels conditional on the disease status.

Figure 3. The GENN model with the best AUC for the TCGA ovarian cancer dataset. Green and orange boxes represent gene expression and methylation features, respectively. W is the weight associated with the feature and PDIV is a division node.

Figure 4. Hypothetical model for the survival time (short-term and long-term) of ovarian cancer. The black sold lines represent the regulatory effects of the eQTM on gene expression. The red dotted lines represent the retrospective simulations of the methylation and gene expression levels conditional on the survival status.

Figure 5. The GENN model constructed by ATHENA using the simulated ovarian cancer data which has a similar structure to the GENN model in Figure 3.

Tables

Table 1. Area under the curve (AUC) for the Three-stage, RFomics, CANetwork, and ATHENA methods under different scenarios

	Three-stage	RFomics	CANetwork	ATHENA
Scenario 1	0.821 (0.028)[1]	0.825 (0.028)	0.626 (0.037)	0.964 (0.017)
Scenario 2	0.501 (0.042)	0.511 (0.038)	0.529 (0.027)	0.509 (0.041)
Scenario 3	0.825 (0.013)	0.845 (0.013)	0.679 (0.019)	0.969 (0.005)

[1]The mean AUC and its standard error estimated based on 100 batches

Table 2. Run time (in seconds) for the Three-stage, RFomics, CANetwork, and ATHENA methods under Scenarios 1 and 3

	Three-stage	RFomics	CANetwork	ATHENA
Scenario 1	72.90	37.78	40.54	39533.91
Scenario 3	230.32	143.91	94.16	113872.50

[1]The mean time (in seconds) was estimated based on 100 batches

 Click here to access/download;Figure;Figure1.jpg

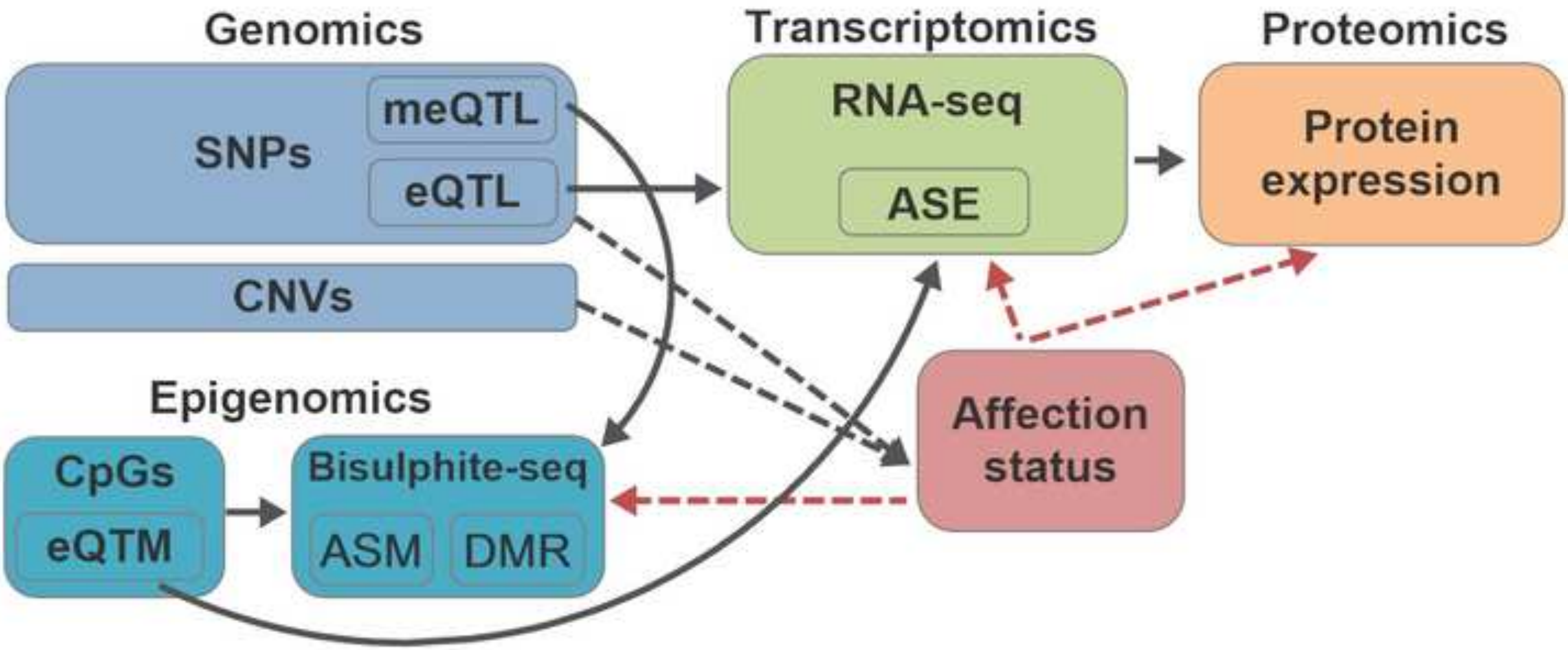

 Click here to access/download;Figure;Figure2.jpg

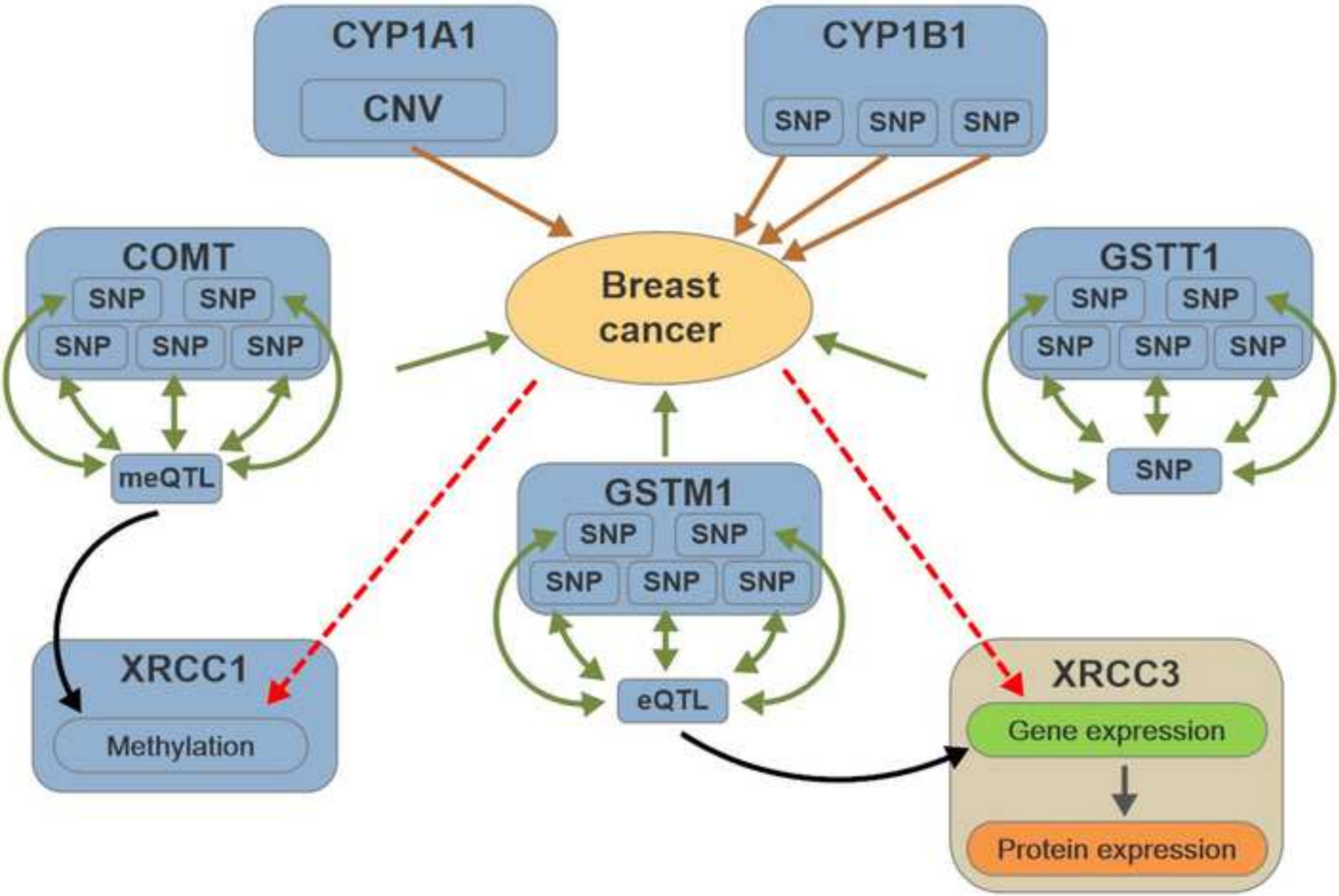

Figure3 Click here to access/download;Figure;Figure3.jpg

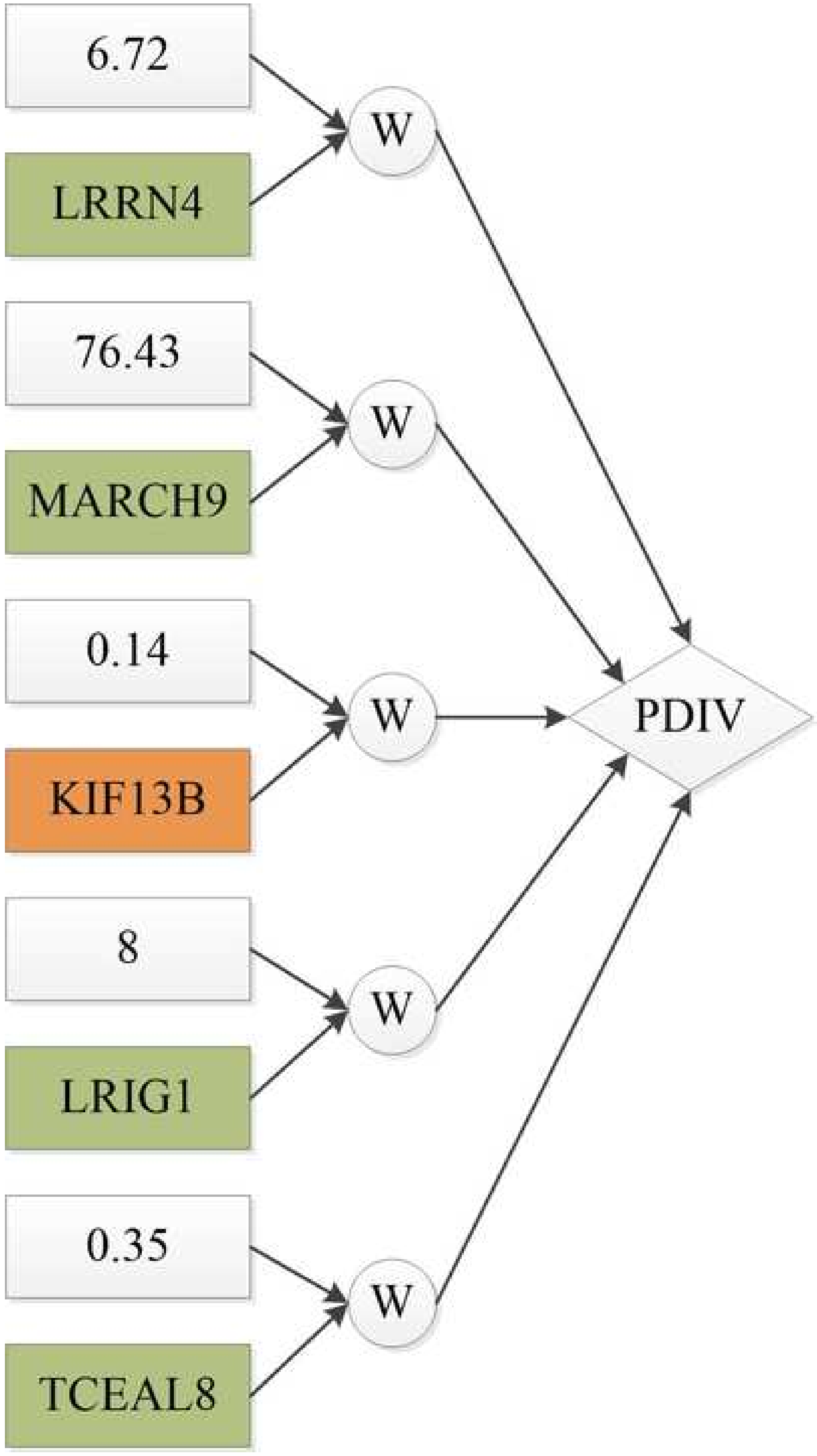

 Click here to access/download;Figure;Figure4.jpg

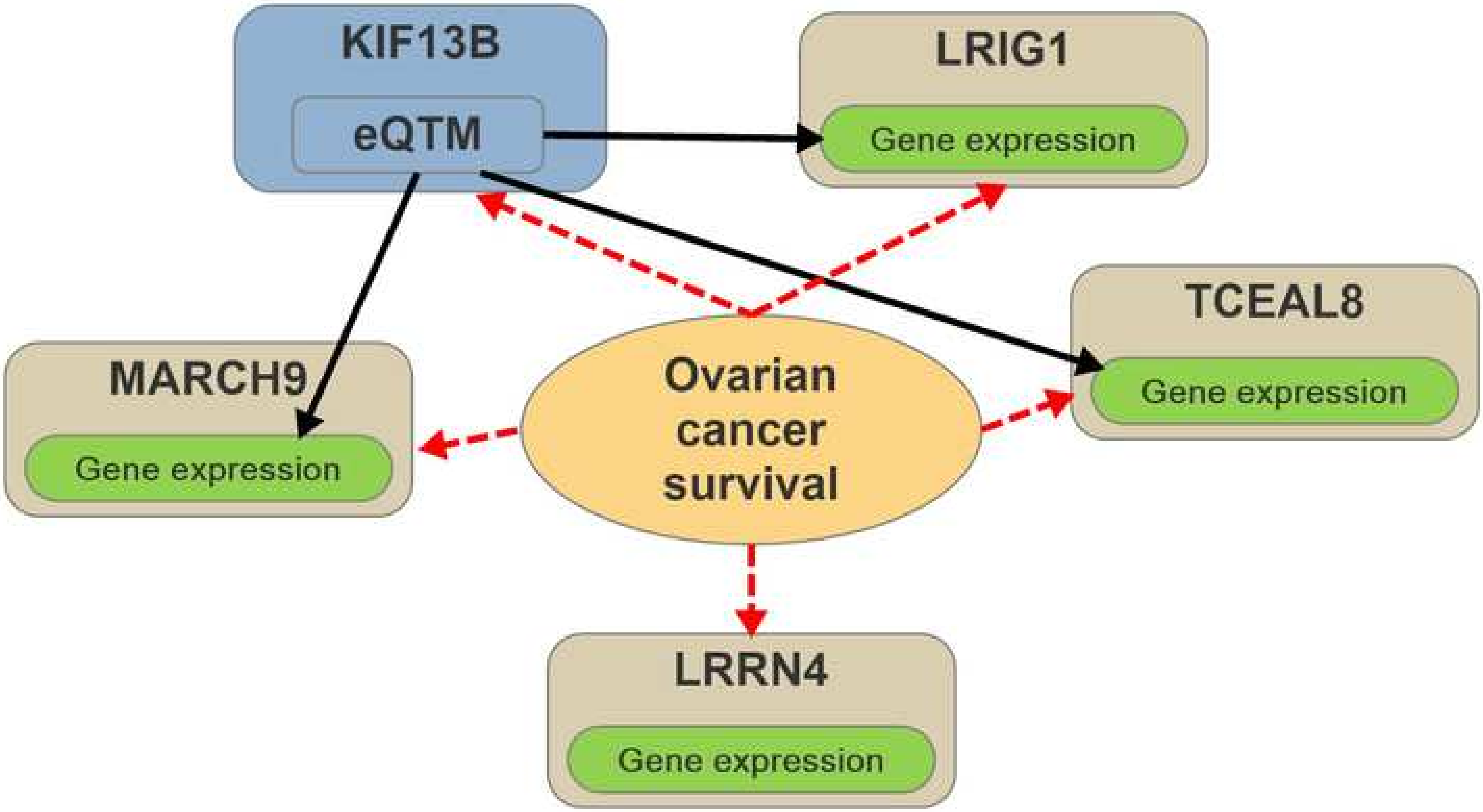

Figure5

Click here to access/download;Figure;Figure5.jpg

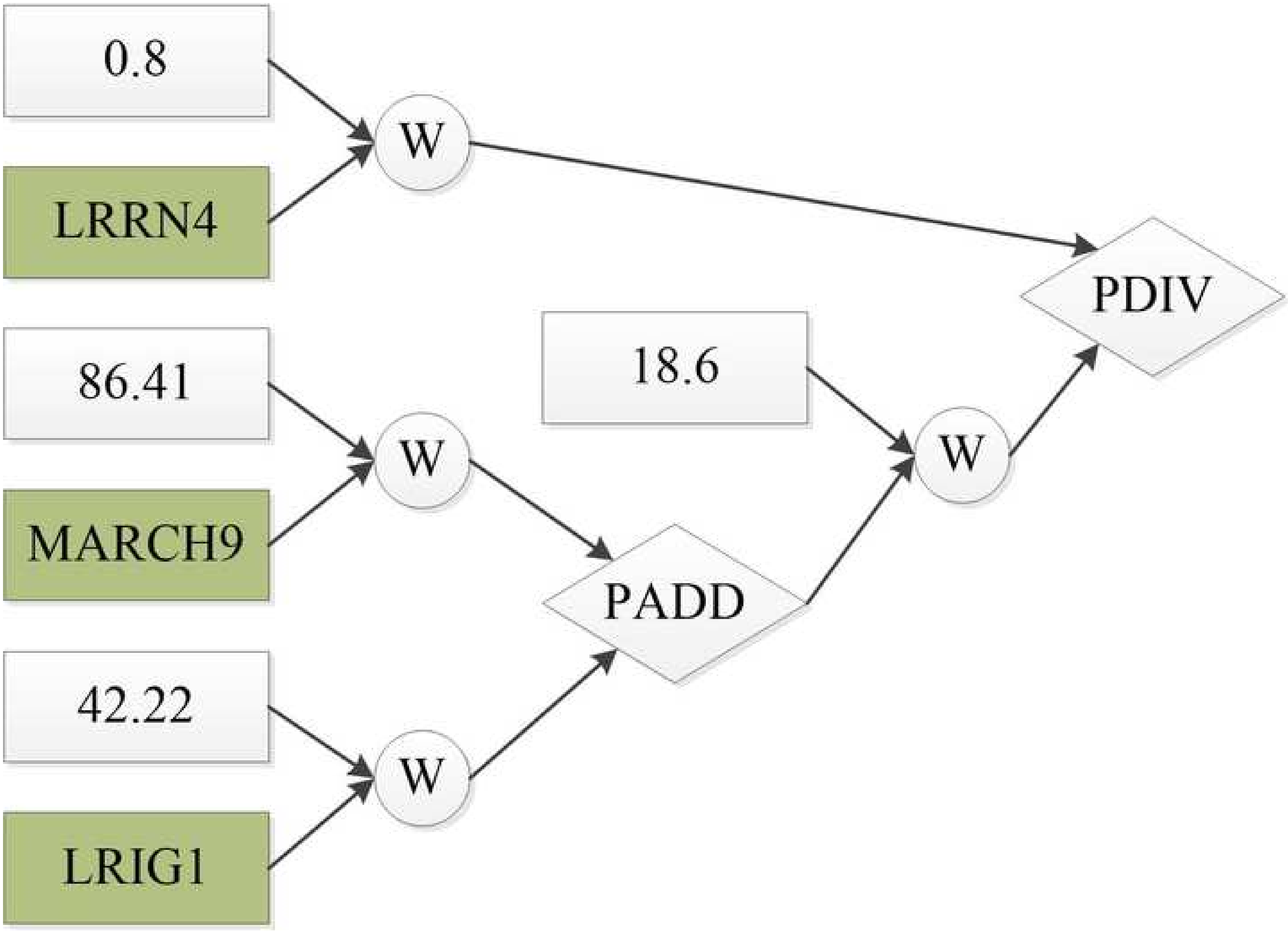

Click here to access/download
Supplementary Material
Supplementary_material_clean.docx
