# Supplementary material for: A multi-omics data simulator for complex disease studies and its application to evaluate multi-omics data analysis methods for disease classification
Source: Gigascience. 2019 Apr 26;8(5):giz045. doi: 10.1093/gigascience/giz045 (PMC6486474; doi:10.1093/gigascience/giz045)
Supplement: Supplemental File [file giz045_supplemental_file.docx]

# Supplementary Methods

## The implementation of the three-stage method

We first performed single-SNP association tests for the 2,022 SNPs with the disease and tested the association between the CNV and the disease using the training dataset. The tests were performed using a logistic regression model. SNPs and CNV with association p-values < 0.05 were identified as significant SNPs and CNV. We performed inverse normal transformation on the methylation rates of the 688 CpG sites. Moreover, the normalized gene and protein expression data produced by OmicsSIMLA were used. Then, a linear regression model was used to test for the associations of the significant SNPs and CNV with the normalized methylation, gene expression, and protein expression data in the training dataset. Each significant SNP and each significant CNV were fitted as a predictor with each methylation, gene expression, and protein expression feature as a response variable in the regression model. A CpG site where at least one SNP and CNV had association p-values < 0.001 (accounting for the multiple testing of approximately 50 CpG sites with variations in methylation rates) with the site was defined as a significant CpG. Similarly, a gene or protein expression feature where at least one SNP and CNV had association p-values < 0.0005 (accounting for the multiple testing of the 100 genes with gene and protein expression data) with the feature was defined as a significant gene or protein expression feature. The significant CpGs and gene and protein expression features were then jointly fitted with a logistic regression model with the disease as the outcome. The model was applied to the validation dataset, and the AUC was calculated.

## Coding of *G_j_* and *G_mn_*

Assume SNP *j* has three genotypes, *AA*, *Aa*, and *aa*, where *a* is the minor allele. *G_j_* are 0, 1, and 2 under an additive model, 0, 2, and 2 under a dominant model, and 0, 0, and 2 under a recessive model. If SNP *j* is in a CNV region, *G_j_* is coded as (*I1* + *I1*$\times$*CNV1* + *I2* + *I2*$\times$*CNV2*). *I1* is 1 if there is a minor allele on the first chromosome and is 0 otherwise. Similarly, *I2* is 1 if there is a minor allele on the second chromosome and is 0 otherwise. *CNV1* is -1, 0, 1, and 2 for deletion, normal, one duplication, and two duplications, respectively, on the first chromosome, and *CNV2* is also -1, 0, 1, and 2 for deletion, normal, one duplication, and two duplications, respectively, on the second chromosome.

For the coding of *G_mn_* at SNPs *m* and *n*, we implemented four epistasis models, including the additive, exclusive OR (XOR), classical epistasis, and a model for describing the genetics of handedness and the color of swine (the color model) [1, 2]. Assume SNP *m* has two alleles *A* and *a* and SNP *n* has two alleles *B* and *b*, where *a* and *b* are the minor alleles. The following tables show the coding of *G_mn_* under the four models:

Additive model:

|  | AA | Aa | aa |
| --- | --- | --- | --- |
| BB | 0 | 0 | 0 |
| Bb | 0 | 1 | 2 |
| bb | 0 | 2 | 4 |

XOR model:

|  | AA | Aa | aa |
| --- | --- | --- | --- |
| BB | 0 | 1 | 0 |
| Bb | 1 | 0 | 1 |
| bb | 0 | 1 | 0 |

Classical epistasis model:

|  | AA | Aa | aa |
| --- | --- | --- | --- |
| BB | 0 | 0 | 1 |
| Bb | 0 | 1 | 0 |
| bb | 1 | 0 | 0 |

Color model:

|  | AA | Aa | aa |
| --- | --- | --- | --- |
| BB | 0 | 1 | 1 |
| Bb | 1 | 0 | 0 |
| bb | 1 | 0 | 0 |

## Generation of the CNV profiles

We downloaded the TCGA focal CNV data for the tumor tissue type using RTCGAToolbox [3], an R package that allows the retrieval of the TCGA pre-processed data from the Firehose pipeline (<http://gdac.broadinstitute.org>). A total of 2,884 genes with significantly altered CNVs across a set of cancer patients based on the GISTIC method were downloaded from cBioPortal (<https://www.cbioportal.org>). We then estimated the CNV frequencies of deletion and duplication in the 2,884 genes for each cancer type in the TCGA data with more than 50 samples. The following table shows the number of samples for each cancer used for the generation of the profiles.

| Disease | Number | Disease | Number |
| --- | --- | --- | --- |
| Adrenocortical carcinoma | 90 | Bladder urothelial carcinoma | 408 |
| Breast invasive carcinoma | 1080 | Cervical and endocervical cancers | 295 |
| Colon adenocarcinoma | 451 | Colorectal adenocarcinoma | 616 |
| Esophageal carcinoma | 184 | Glioblastoma multiforme | 577 |
| Glioma | 1090 | Head and Neck squamous cell carcinoma | 522 |
| Kidney Chromophobe | 66 | Pan-kidney cohort | 882 |
| Kidney renal clear cell carcinoma | 528 | Kidney renal papillary cell carcinoma | 288 |
| Brain Lower Grade Glioma | 80 | Liver hepatocellular carcinoma | 370 |
| Lung adenocarcinoma | 516 | Lung squamous cell carcinoma | 501 |
| Mesothelioma | 87 | Ovarian serous cystadenocarcinoma | 579 |
| Pancreatic adenocarcinoma | 184 | Pheochromocytoma and Paraganglioma | 162 |
| Prostate adenocarcinoma | 492 | Rectum adenocarcinoma | 165 |
| Sarcoma | 257 | Stomach adenocarcinoma | 80 |
| Stomach and Esophageal carcinoma | 625 | Testicular Germ Cell Tumors | 150 |
| Thyroid carcinoma | 499 | Thymoma | 123 |
| Uterine Corpus Endometrial Carcinoma | 539 | Uterine Carcinosarcoma | 56 |
| Uveal Melanoma | 80 |  |  |

## Generation of the RNA-seq profiles

We also used RTCGAToolbox to download the TCGA RNA-seq data (i.e., RSEM counts) for both tumor and normal tissue types. A tumor or normal tissue type with more than 50 samples was used for the generation of the profiles. The following table shows the numbers of samples for the tumor and normal tissue types.

| Disease | Type | Number | Disease | Type | Number |
| --- | --- | --- | --- | --- | --- |
| Bladder urothelial carcinoma | Tumor | 408 | Breast invasive carcinoma | Normal | 112 |
| Breast invasive carcinoma | Tumor | 1100 | Cervical and endocervical cancers | Tumor | 306 |
| Colorectal adenocarcinoma | Normal | 51 | Colorectal adenocarcinoma | Tumor | 382 |
| Colon adenocarcinoma | Tumor | 287 | Esophageal carcinoma | Tumor | 185 |
| Glioma | Tumor | 696 | Glioblastoma multiforme | Tumor | 166 |
| Head and Neck squamous cell carcinoma | Tumor | 522 | Kidney Chromophobe | Tumor | 66 |
| Pan-kidney cohort | Normal | 129 | Pan-kidney cohort | Tumor | 891 |
| Kidney renal clear cell carcinoma | Normal | 72 | Kidney renal clear cell carcinoma | Tumor | 534 |
| Kidney renal papillary cell carcinoma | Tumor | 291 | Acute Myeloid Leukemia | Tumor | 173 |
| Brain Lower Grade Glioma | Tumor | 530 | Liver hepatocellular carcinoma | Normal | 50 |
| Liver hepatocellular carcinoma | Tumor | 373 | Lung adenocarcinoma | Normal | 59 |
| Lung adenocarcinoma | Tumor | 517 | Lung squamous cell carcinoma | Normal | 51 |
| Lung squamous cell carcinoma | Tumor | 501 | Ovarian serous cystadenocarcinoma | Normal | 51 |
| Ovarian serous cystadenocarcinoma | Tumor | 307 | Pancreatic adenocarcinoma | Tumor | 179 |
| Pheochromocytoma and Paraganglioma | Tumor | 184 | Prostate adenocarcinoma | Normal | 52 |
| Prostate adenocarcinoma | Tumor | 498 | Rectum adenocarcinoma | Tumor | 95 |
| Sarcoma | Tumor | 263 | Skin Cutaneous Melanoma | Tumor | 472 |
| Stomach adenocarcinoma | Tumor | 415 | Stomach and Esophageal carcinoma | Tumor | 600 |
| Testicular Germ Cell Tumors | Tumor | 156 | Thyroid carcinoma | Normal | 59 |
| Thyroid carcinoma | Tumor | 509 | Thymoma | Tumor | 120 |
| Uterine Corpus Endometrial Carcinoma | Tumor | 177 |  |  |  |

The parameters ***c*** (a vector of normalization factors for the individuals), $\lambda$ and $\omega$ for all genes were estimated using the R package edgeR [4]. Genes with *λ* values (i.e., means of the expression count) less than 10 were excluded from the profiles.

## Calculating the individual-specific normalization factor

We used the smoothed bootstrap procedure [5] to calculate the individual-specific normalization factor $c_{j}^{*}$. Assume ***c*** =$(c_{1},c_{2},...,c_{k})$ is a vector consisting of the individual-specific normalization factors estimated based on *k* individuals from the TCGA project. To simulate *n* individual-specific normalization factors, we first sample *n* values ($y_{1}^{*},y_{2}^{*},...,y_{n}^{*}$) from ***c*** with replacement. Then $c_{j}^{*}$ is calculated as

$$c_{j}^{*}=\bar{y}^{*}+\frac{y_{j}^{*}-\bar{y}^{*}+\hat{h}_{1}\varepsilon_{j}}{\sqrt{1+\hat{h}_{1}^{2}/\hat{\sigma}^{2}}}$$

where $\bar{y}^{*}$ is the mean of ($y_{1}^{*},y_{2}^{*},...,y_{n}^{*}$), $\hat{\sigma}^{2}$ is the sample variance of $(c_{1},c_{2},...,c_{k})$, $\hat{h}_{1}$ is 0.05, and $\varepsilon_{j}$ is simulated from a standard normal distribution. Assuming the sample variance of ($y_{1}^{*},y_{2}^{*},...,y_{n}^{*}$) is approximately $\hat{\sigma}^{2}$, it can be easily shown that the mean and variance of ($c_{1}^{*},c_{2}^{*},...,c_{n}^{*}$) will be approximately $\bar{y}^{*}$and $\hat{\sigma}^{2}$, respectively.

## Generation of the RPPA profiles

We also used RTCGAToolbox to download the TCGA RPPA data for different tumor tissue types. A tumor tissue type with more than 50 samples was used for the generation of the profiles. The following table shows the numbers of samples for the tumor types used for the calculations.

| Disease | Number | Disease | Number |
| --- | --- | --- | --- |
| Bladder urothelial carcinoma | 344 | Breast invasive carcinoma | 937 |
| Colorectal adenocarcinoma | 493 | Colon adenocarcinoma | 362 |
| Esophageal carcinoma | 126 | Glioma | 679 |
| Glioblastoma multiforme | 244 | Head and Neck squamous cell carcinoma | 212 |
| Kidney Chromophobe | 63 | Pan-kidney cohort | 757 |
| Kidney renal clear cell carcinoma | 478 | Kidney renal papillary cell carcinoma | 216 |
| Brain Lower Grade Glioma | 435 | Liver hepatocellular carcinoma | 184 |
| Lung adenocarcinoma | 365 | Lung squamous cell carcinoma | 328 |
| Ovarian serous cystadenocarcinoma | 436 | Pheochromocytoma and Paraganglioma | 82 |
| Prostate adenocarcinoma | 352 | Skin Cutaneous Melanoma | 355 |
| Stomach adenocarcinoma | 357 | Stomach and Esophageal carcinoma | 483 |
| Thyroid carcinoma | 224 | Thymoma | 90 |
| Uterine Corpus Endometrial Carcinoma | 440 | Uterine Carcinosarcoma | 48 |

## Calculating the risk score of a gene in RFomics

For a set of SNPs in a gene, we first use LASSO logistic regression to select a subset of SNPs associated with the disease. Assume that *k* SNPs are selected by the LASSO regression and their regression coefficients are $\left(\beta_{1},\beta_{2},...,\beta_{k}\right)$, where their minor alleles are the reference alleles. We define risk alleles as the alleles with risk effects (i.e., the alleles with positive regression coefficients) on the disease. The risk score *G* of the gene is calculated as

$G=\sum_{i=1}^{k} \left|\beta_{i}\right|g_{i}$, where *g_i_* is the risk allele count at SNP *i*. Then each variable from different omics data, including the gene-based risk scores, CNV statuses of genes, methylation proportions at CpGs, gene and protein expression levels, is normalized so that it has a mean 0 and a standard deviation of 1. The RFomics used the normalized variables for classification.

## Simulation studies for the hypothetical breast cancer pathways

We used COSI [6] to simulate 10,000 haplotypes with lengths of 8,577, 28,235, 10,410, and 8,149 bps for the CYP1B1, COMT, GSTM1, and GSTT1 genes, respectively. The sizes of the genes are based on the GRCh37.p13 assembly. We also simulated a region with length of 30,000 bps containing regulatory variants. There were 200, 687, 264, and 176 SNPs in the CYP1B1, COMT, GSTM1, and GSTT1 genes, respectively, and 695 SNPs in the regulatory region. The haplotypes were then used as the reference sequences in OmicsSIMLA to simulate samples. As described in the manuscript, 3 common variants in CYP1B1, 5 rare variants in COMT, 5 rare variants in GSTM1, and 5 rare variants in GSTT1 were randomly selected as disease sites. The 3 common variants in CYP1B1 had main effects (ORs = 1.5), the 5 variants in COMT each had an interaction effect (ORs = 5) with a meQTL (with an MAF of 34% located in the regulatory region) for the XRCC1 gene, the 5 variants in GSTM1 each had an interaction effect (ORs = 5) with a cis-eQTL (with an MAF of 33% located in the regulatory region) for XRCC3, and the 5 variants in GSTT1 each had an interaction effect (ORs = 5) with a trans-eQTL (with an MAF of 23% located in the regulatory region) for XRCC3. We also simulated a deletion (with a frequency of 20%), which had an OR of 0.67 in the CYP1A1 gene. The CNV and the disease SNPs were used to generate the disease status for each sample. Furthermore, CpGs in the XRCC1 gene region (19:44038000-44079730) were simulated, and their methylation rates were based on the “H1 derived mesenchymal stem cells” profile in pWGBSSimla. We assumed 20% of unmethylated blocks had differential methylation and the difference in methylation rates between cases and controls was assumed to be 10%. We also simulated gene expressions for 100 genes, consisting of the expression for XRCC3 and 99 other equivalently expressed genes between cases and controls. The cis- and trans-eQTLs for XRCC3 both caused 1.5 fold changes of the gene expression for the non-reference genotypes relative to the reference genotypes. We also assumed that there were 1.5 fold changes of the gene expression for XRCC3 in cases relative to that in controls. Finally, protein expression levels for the 100 genes were simulated incorporating the simulated gene expression data.

For Scenario 1, we simulated 500 cases and 500 controls for the training dataset and 100 cases and 100 controls for the validation dataset. For Scenario 2, the same sample sizes were simulated but we changed the effects of CNV, SNPs, and gene expression on the disease. To be more specific, the 3 common variants in CYP1B1 had main effects with ORs of 1.2, and the rare variants in COMT, GSTM1, and GSTT1 all had interaction effects with ORs of 3. The difference in methylation rates between cases and controls was assumed to be 5%. The cis- and trans-eQTL for XRCC3 both caused 1.2 fold changes of the gene expression for the non-reference genotypes relative to the reference genotypes, and there were 1.2 fold changes of the gene expression for XRCC3 in cases relative to controls. For Scenario 3, we simulated 1,500 cases and 1,500 controls for the training dataset, and 500 cases and 500 controls for the validation dataset. The effects of CNV, SNPs, and gene expression were the same as those in Scenario 1.

**Generation of the TCGA ovarian cancer profiles**

A CNV information file for OmicsSIMLA should have the CNV types, CNV frequencies, and odds ratios of the CNVs for the phenotype. The CNV frequencies for the CNV types (i.e., deletions and duplication) were estimated based on the 2,884 focal CNVs. The odds ratios for all CNV types were specified to be 1 because no significant CNVs were identified by ATHENA in the OV data. The methylation profiles for OmicsSIMLA have the information for the distances between CpGs, methylation rates, methylation status, and read counts for each type of methylation status. Methylation data were generated based on the Illumina Infinium HumanMethylation27 BeadChip, and the data contained 25,794 CpGs. To generate NGS read counts in the methylation profiles, we identified overlapping CpGs between the OV data and the colon cancer WGBS data from Ziller et al. [7]. A total of 24,743 overlapping CpGs were identified. The methylation rates and read counts were calculated for the 24,743 CpGs based on the colon cancer data. The gene expression profiles for OmicsSIMLA have the normalization factors for a group of samples and the mean and dispersion parameter of the negative binomial distribution for each gene. These parameters were estimated based on the gene expression levels of the 17,946 genes in the OV data. Finally, the protein expression profiles have the standard deviations of the normalized protein expression levels. They were estimated based on the protein expression levels of the 204 genes in the OV data.

## References

1. Wan X, Yang C, Yang Q, Xue H, Fan X, Tang NL, et al. BOOST: A fast approach to detecting gene-gene interactions in genome-wide case-control studies. American journal of human genetics. 2010;87 3:325-40. doi:10.1016/j.ajhg.2010.07.021.

2. Chung RH and Kang CY. A Powerful Gene-Based Test Accommodating Common and Low-Frequency Variants to Detect Both Main Effects and Gene-Gene Interaction Effects in Case-Control Studies. Frontiers in genetics. 2017;8:228. doi:10.3389/fgene.2017.00228.

3. Samur MK. RTCGAToolbox: a new tool for exporting TCGA Firehose data. PloS one. 2014;9 9:e106397. doi:10.1371/journal.pone.0106397.

4. Robinson MD, McCarthy DJ and Smyth GK. edgeR: a Bioconductor package for differential expression analysis of digital gene expression data. Bioinformatics. 2010;26 1:139-40. doi:10.1093/bioinformatics/btp616.

5. Efron B and Tibshirani RJ. An Introduction to the Bootstrap. Chapman and Hall/CRC; 1993.

6. Schaffner SF, Foo C, Gabriel S, Reich D, Daly MJ and Altshuler D. Calibrating a coalescent simulation of human genome sequence variation. Genome research. 2005;15 11:1576-83. doi:10.1101/gr.3709305.

7. Ziller MJ, Gu H, Muller F, Donaghey J, Tsai LT, Kohlbacher O, et al. Charting a dynamic DNA methylation landscape of the human genome. Nature. 2013;500 7463:477-81. doi:10.1038/nature12433.

# Supplementary Tables

## Table S1. Parameters for ATHENA and random forest

| **Parameter** | **Value** |
| --- | --- |
| **ATHENA** | |
| Number of demes (CPUs) | 20 |
| Population size/deme | 5000 |
| Number of generations | 300 |
| Number of migrations | 15 |
| Probability of crossover | 0.9 |
| Probability of mutation | 0.01 |
| Fitness function | Balanced accuracy in case/control set |
| **Random forest** | |
| Number of trees | 500 |
| Number of variables at each split | Square root of the total number of variables |
| Minimum size of terminal nodes | 1 feature |

## Table S2. CNV states considered in OmicsSIMLA

| CNV states on the two chromosomes | Total copy number on the two chromosomes | Description |
| --- | --- | --- |
| D/D | 0 | Both genes are deleted |
| D/N | 1 | One gene is deleted on one chromosome, and the gene on the other chromosome is normal (one copy) |
| D/U | 2 | The gene is deleted on one chromosome but has two copies on the other chromosome |
| N/N | 2 | Normal copies of the genes on both chromosomes |
| D/UU | 3 | The gene is deleted on a chromosome but has three copies on the other chromosome |
| N/U | 3 | The gene has two copies on one chromosome, and normal copy on the other chromosome |
| N/UU | 4 | The gene has three copies on one chromosome, and normal copy on the other chromosome |
| U/U | 4 | The gene has two copies on each chromosome |
| U/UU | 5 | The gene has 2 copies on one chromosome and 3 copies on the other chromosome |
| UU/UU | 6 | The gene has 3 copies on each chromosome |

D: deletion, N: normal (one copy), U: one duplication (two copies), UU: two duplication (three copies)

## Table S3. Pearson correlation coefficients among the features selected by ATHENA

|  | LRRN4 | MARCH9 | KIF13B | LRIG1 | TCEAL8 |
| --- | --- | --- | --- | --- | --- |
| LRRN4 | 1 | -0.0036 | -0.0009 | 0.0011 | 0.0975 |
| MARCH9 | -0.0036 | 1 | **0.1833^1^** | **0.2746** | **0.5690** |
| KIF13B | -0.0009 | **0.1833** | 1 | 0.0617 | **0.1842** |
| LRIG1 | 0.0011 | **0.2746** | 0.0617 | 1 | **0.2560** |
| TCEAL8 | 0.0975 | **0.5690** | **0.1842** | **0.2560** | 1 |

^1^Correlation coefficients marked with bold have significant correlations (p-values < 0.05)
