# Supplementary material for: A multi-omics data simulator for complex disease studies and its application to evaluate multi-omics data analysis methods for disease classification
Source: Gigascience. 2019 Apr 26;8(5):giz045. doi: 10.1093/gigascience/giz045 (PMC6486474; doi:10.1093/gigascience/giz045)
Supplement: Response_to_Reviewer_Comments_Original_Submission.pdf [file giz045_response_to_reviewer_comments_original_submission.pdf]

Author's Response To Reviewer Comments

Close

Editor’s comments:
1. The manuscript addresses an important problem. However, overall, you need to provide more practical examples in order to demonstrate its validity. Because there are several papers that already demonstrate the data integration effect predicting outcomes in a TCGA dataset, we ask that you simulate multi-omics data using OmicsSIMLA and compare the results between the simulation data and real dataset based on previous literature. You also need to highlight how OmicsSIMLA compares to other similar tools, for example, HIBACHI (https://www.ncbi.nlm.nih.gov/pmc/articles/PMC4270828/).
Response:
Thank the Editor for the comments. We used OmicsSIMLA to simulate a multi-omics dataset based on the ovarian cancer (OV) data, consisting of CNVs, methylation, gene expression, and protein expression data, downloaded from the TCGA project. More details can be found in the response to the first comment of Reviewer 1. We have also highlighted how OmicsSIMLA compares to other similar tools, which can be seen in the response to the first comment of Reviewer 2.

2. In addition, please register any new software application in the SciCrunch.org database to receive a RRID (Research Resource Identification Initiative ID) number, and include this in your manuscript. This will facilitate tracking, reproducibility and re-use of your tool.
Response:
We have registered our software in the SciCrunch database with a RRID of SCR_017011. We have also included the RRID in the manuscript.

Reviewer 1’s comments:
1. The evaluation scheme for the proposed simulation tool is not strong. Simulating multi-omics data based on the hypothetical example is great, but they should provide more practical examples in order to demonstrate its validity. There have been many papers that show the data integration effect predicting outcomes using TCGA dataset. So, I would suggest that they should simulate multi-omics data using OmicsSIMLA and compared the results between the simulation data and real dataset based on the previous literature.
Response:
Thank the Reviewer for the very constructive comments. We have used OmicsSIMLA to simulate a multi-omics dataset based on the ovarian cancer (OV) data, consisting of CNVs, methylation, gene expression, and protein expression data, downloaded from the TCGA project. We first applied ATHENA to the TCGA OV data for classifying the survival time (i.e., long-term and short-term survival) similar to the analysis in Kim et al. (BioData Mining 2013). We then simulated a multi-omics data based on the OV data with the model identified by ATHENA. Finally, ATHENA was applied to the simulated data. Our analysis results demonstrated that OmicsSIMLA can generate data with a scale similar to the OV data, and the analysis results from ATHENA between the real and simulated data were comparable. The results for the simulations are described starting from the last paragraph on page 14, and how we performed the simulations is described from page 29.

2. As they introduced multi-staged and meta-dimensional approaches in data integration, they need to show the simulation results based on two different approaches (scenarios). Researchers have been developing data integration methods based on either multi-staged or meta-dimensional models, they might need OmicsSIMLA with different purpose.
Response:
We have included a three-staged method (Holzinger and Ritchie, Pharmacogenomics, 2012) in our simulation studies to compare with the meta-dimensional approaches. The implementation of the three-stage method is described in the last paragraph on page 12 and the Supplementary methods. The results for the comparisons of the three-staged and meta-dimensional approaches are described starting from the last paragraph on page 13.

3. It is good to use three different machine learning methods, but specific sets of parameters (such as population size, generation number, migration, etc for ATHENA) per method are missing. Depending on

different sets of parameters, results can be very different. They should provide this info in the methods section.
Response:
We have provided the parameter values we used for simulations for ATHENA and random forest in Supplementary Table S1. The Reviewer is correct that the analysis results can be very different depending on different sets of parameters. When we increased the number of demes, the number of generations, and the number of migrations in ATHENA, the AUC for ATHENA increased significantly, as shown in Table 1, and ATHENA now showed the highest AUC compared to other methods. The results are described in the last paragraph on page 13.

4. So, it is the classification problem, but what it eh label?
Response:
The label is the disease status. We have clarified this in the second paragraph on page 12.

5. I think there is a wrong subsection header name in the method section. For the normalization section: A random-forest based method for integrating multi-omics data for disease studies?
Response:
The section was intended to describe how we normalized data for random forest. Since we have now emphasized that ATHENA would be the best tool to perform multi-omics data analysis, we have moved the section to the Supplementary methods and integrated the section with "Calculating the risk score of a gene in RFomics."

6. The manual was well described. It would be great if they add the tutorial for the hypothetical example as well as additional scenarios based on TCGA real dataset.
Response:
We have added the tutorials for the hypothetical example and the scenario based on TCGA real dataset in the user manual, which can be accessed here:
https://omicssimla.sourceforge.io/simuomicsTCGA.html.

Reviewer 2's comments:
1. This manuscript presents a very interesting expansion to existing simulation tools by adding various 'omics simulations possible. This is definitely a requirement in the field, though I want to mention that there are other tools like HIBACHI (https://www.ncbi.nlm.nih.gov/pmc/articles/PMC4270828/) which also aim to achieve similar integrated framework to mimic the biology and interactions among various omic datasets to simulate the data. Authors must revise the sentences to mention how OmicSIMLA is different and also is not the first comics simulator.
Response:
Thank the Reviewer for the very constructive comments. We have revised the statement and reviewed the currently available multi-omics data simulators, such as HIBACHI, InterSIM, and MOSim. The review is provided in the second paragraph on page 6. Furthermore, we discussed the advantages and disadvantages of using these tools starting from the last paragraph on page 16.

2. Authors do a nice job of breaking down what the required input files are for simulating each specific data type (genotype, CNV, WGBSS, gene expression, and protein expression); however, the role of "Profiles" is a bit unclear. For example, gene expression "Profile" data contains gene expression beta values, but whether a user is to input their own beta values or if there is a standard is unclear. Additionally, the gene expression profile data contains individual-specific normalization factors (mean and dispersion parameters) to create the negative binomial distribution. The file contains values for 103 normal tissue but there is no information on how to actually use or interpret this data. Please add more clarity on the same.
Response:
The beta values in the gene expression profiles are no longer used. We have removed them from the gene expression profiles. We have explained in more details about how to use or interpret the individual-specific normalization factors. The individual-specific normalization factor is used to model the systematic variations among individuals due to technical variation. If no technical variation will be simulated, the user can replace c with a vector of 1, where the length of the vector is the number of samples to be simulated. We have clarified the use and interpretation of the normalization factors and the parameters for the negative binomial distributions in the last paragraph on page 23.

3. There is methylation profile data which is specific to 29 cell types and tissues. This is quite easy to maneuver when generating methylation simulations. There is a protein expression profile which contains standard deviations for the normal distribution. The parameters are based on 874 breast cancer patients

from the TCPA. If the gene expression and protein expression profiles were also based on tissue type (maybe something like GTEx), that would be really useful.
Response:
Thank the Reviewer for the suggestion. We have compiled profiles of different cancer tissue types for the CNV, gene expression, and protein expression profiles. To be more specific, the CNV profiles are specific to 33 cancer tissues, the gene expression profiles are specific to normal and tumor tissues for 31 cancers, and the protein expression profiles are specific to 26 cancers. These profiles were compiled based on the TCGA data. We described the profiles in the last paragraph on page 9 and how we generated the profiles in the Supplementary Methods.

4. To summarize above two points, more details about the role and application of "profile" data would be helpful and why the data is skewed for certain distributions. Do users need to use the same profiles to generate simulations, and if not, does their data need to fit a certain distribution?
Response:
We provided profiles for simulating the CNV, methylation, gene expression, and protein expression data. To use the profiles, the user simply specify the tissue types and the number of samples to be simulated and OmicsSIMLA will simulate datasets based on the parameters in the profiles. Alternatively, the user can compile their own profiles with the formats and assumptions of data distributions that are clearly described in the user manual. For example, negative binomial distributions are assumed for the RNA-seq read counts, and normal distributions are assumed for the protein expression data. We described the use of the profiles starting from the last paragraph on page 9.

5) For the input file in SeqSIMLA, a variety of reference files can be input to bolster specificity of the simulated genotypes, i.e. refseq, penetrance, pedigree structure, etc. Most of these are in PLINK file format so that is good. However, what is a bit unclear is how much of this information is required to generate simulated data. For example, in HIBACHI, you could specify the bare minimum parameters to generate a simulation, of course with the drawback being that the data had no biological context. Here it is evident the data has biological context, but there should be more information for use case; how much/which type of data is required to generate the genotype simulation? It is not very clear what is "required" and what is "optional".
Response:
As there are many input files and parameters in OmicsSIMLA, some are required or optional depending on the simulation scenarios. The best way for the user to learn the required and optional files and parameters in OmicsSIMLA is to use the web interface (https://omicssimla.sourceforge.io/generateCommand.html). The required fields are marked with red * on the web interface. If certain simulations are selected by the user, more options will appear for inputs. The interface will also check the inputs from the user. If some required fields are missing from the inputs, the interface will remind the user. We have described the input files and parameters for OmicsSIMLA starting from the last paragraph on page 9 to page 11. On page 11, we encourage the user to use the web interface to learn the required and optional files and parameters for OmicsSIMLA.

6) It appears that this tool is best used if you have datasets already but are missing a specific desired dataset. For example, if you have genotype and methylation, and you know the data well enough to expect values for missing protein expression data type. Or if you have very specific requirements for the type of simulated data you want. This tool doesn't seem like the best option if you are simulating all datatypes from scratch, authors should clarify if this is the case because this point is not mentioned in the manuscript. And if this is not the case, more clarification on how all data types can be generated from scratch would be very useful.
Response:
Three main components are required to perform the simulations in OmicsSIMLA, including a biological model (i.e., the disease model), reference sequences and profiles, and parameter values to model the effects of the multi-omics features on the disease and to model the relationships between multi-omics data. A biological model should be hypothesized by the user before performing the simulations. We have compiled the reference sequences for different human populations and profiles for many tissue types so that the user can conveniently choose the appropriate files for the simulations. Furthermore, we have made suggestions for many of the parameter values based on the literatures or the observations in real data. The pre-compiled files and recommended values will minimize the efforts for the user to perform the simulation from scratch. We have discussed the best use of OmicsSIMLA starting from the last paragraph on page 18.

7) Another example of the last comment is that for the tutorial, they provide the following 3 files: 1) a reference .bim file containing 2022 SNPs, 2) a .txt file specifying site interaction effects between SNPs

using an odds ratio, and 3) a .txt file containing information on CNVs between sites and the frequency of deletions/odds ratio of deletion. Therefore, it seems to me you are required to have a good amount of input data in order to generate the simulations.
Response:
Same as the response to the previous comment, we have pre-compiled several files to minimize the efforts for the user to generate the simulations.

8) For simulating gene expression data, it seems that beta values are required. It would be beneficial to mention where to obtain beta values, should these always be constant from public resources like GTex?
Response:
The beta values are no longer used. We have removed them from the profiles.

9) I used the tutorials to generate simulations: for the most part they were as expected. However, when simulating multi-omics data, I was unable to generate a .cnv file as is outlined in the tutorial under "Several files will be generated:".
Response:
We have executed the command again as described in the tutorials and it seemed that the cnv file can be generated. Please contact us for any technical supports to simulate the file.

10) Additionally, upon generating successful simulations, I received the following error every time:
sh: ../bin/bug_reporter: cannot execute binary file
However, I have not found any documentation on bug_reporter after compilation. I am not sure exactly what is meant by the error.
Response:
We have fixed the bug.

Close
