# Supplementary material for: A multi-omics data simulator for complex disease studies and its application to evaluate multi-omics data analysis methods for disease classification
Source: Gigascience. 2019 Apr 26;8(5):giz045. doi: 10.1093/gigascience/giz045 (PMC6486474; doi:10.1093/gigascience/giz045)
Supplement: Response_to_Reviewer_Comments_Revision_1.pdf [file giz045_response_to_reviewer_comments_revision_1.pdf]

Author's Response To Reviewer Comments

Close

Dear Dr. Nogoy,
We have revised the manuscript based on your comments. Thank you very much.
Sincerely,
Ren-Hua

Ren-Hua Chung, PhD
Associate Investigator
Division of Biostatistics and Bioinformatics
Institute of Population Health Sciences
National Health Research Institutes
Taiwan

Close
