# Supplementary material for: A multi-omics data simulator for complex disease studies and its application to evaluate multi-omics data analysis methods for disease classification
Source: Gigascience. 2019 Apr 26;8(5):giz045. doi: 10.1093/gigascience/giz045 (PMC6486474; doi:10.1093/gigascience/giz045)
Supplement: Reviewer_1_Report_Original_Submission -- Dokyoon Kim — 11/11/2018 Reviewed [file giz045_reviewer_1_report_original_submission.pdf]

Reviewer Report

Title: A multi-omics data simulator for complex disease studies and its application to evaluate multi-omics data analysis methods for disease classification

Version: Original Submission **Date:** 11/11/2018

Reviewer name: Dokyoon Kim

Reviewer Comments to Author:

This paper addresses very important problem. As data integration is getting really important, there is no available simulation tool.
I have personally searched the existing simulation tools for generating multi-omics data, but I was not able to find the relevant one.
Thus, I think OmicsSIMLA could be a great tool for many researchers, however, there are a couple of concerns and comments in order to improve the manuscript.
- The evaluation scheme for the proposed simulation tool is not strong.
Simulating multi-omics data based on the hypothetical example is great, but they should provide more practical examples in order to demonstrate its validity.
There have been many papers that show the data integration effect predicting outcomes using TCGA dataset.
So, I would suggest that they should simulate multi-omics data using OmicsSIMLA and compared the results between the simulation data and real dataset based on the previous literature.
- As they introduced multi-staged and meta-dimensional approaches in data integration, they need to show the simulation results based on two different approaches (scenarios).
Researchers have been developing data integration methods based on either multi-staged or meta-dimensional models, they might need OmicsSIMLA with different purpose.
- It is good to use three different machine learning methods, but specific sets of parameters (such as population size, generation number, migration, etc for ATHENA) per method are missing.
Depending on different sets of parameters, results can be very different.
They should provide this info in the methods section.
- So, it is the classification problem, but what it eh label?
- I think there is a wrong subsection header name in the method section.
For the normalization section: A random-forest based method for integrating multi-omics data for disease studies?
- The manual was well described.
It would be great if they add the tutorial for the hypothetical example as well as additional scenarios based on TCGA real dataset.

Methods

Are the methods appropriate to the aims of the study, are they well described, and are necessary controls included? Choose an item.

Conclusions

Are the conclusions adequately supported by the data shown? Choose an item.

Reporting Standards

Does the manuscript adhere to the journal's guidelines on minimum standards of reporting? Choose an item.

Choose an item.

Statistics

Are you able to assess all statistics in the manuscript, including the appropriateness of statistical tests used? Choose an item.

Quality of Written English

Please indicate the quality of language in the manuscript: Choose an item.

Declaration of Competing Interests

Please complete a declaration of competing interests, considering the following questions:

- Have you in the past five years received reimbursements, fees, funding, or salary from an organisation that may in any way gain or lose financially from the publication of this manuscript, either now or in the future?
- Do you hold any stocks or shares in an organisation that may in any way gain or lose financially from the publication of this manuscript, either now or in the future?
- Do you hold or are you currently applying for any patents relating to the content of the manuscript?
- Have you received reimbursements, fees, funding, or salary from an organization that holds or has applied for patents relating to the content of the manuscript?
- Do you have any other financial competing interests?
- Do you have any non-financial competing interests in relation to this paper?

If you can answer no to all of the above, write 'I declare that I have no competing interests' below. If your reply is yes to any, please give details below.

I declare that I have no competing interests.

I agree to the open peer review policy of the journal. I understand that my name will be included on my report to the authors and, if the manuscript is accepted for publication, my named report including any attachments I upload will be posted on the website along with the authors' responses. I agree for my

report to be made available under an Open Access Creative Commons CC-BY license (http://creativecommons.org/licenses/by/4.0/). I understand that any comments which I do not wish to be included in my named report can be included as confidential comments to the editors, which will not be published.

I agree to the open peer review policy of the journal

To further support our reviewers, we have joined with Publons, where you can gain additional credit to further highlight your hard work (see: https://publons.com/journal/530/gigascience). On publication of this paper, your review will be automatically added to Publons, you can then choose whether or not to claim your Publons credit. I understand this statement.

Yes Choose an item.
