# Supplementary material for: A multi-omics data simulator for complex disease studies and its application to evaluate multi-omics data analysis methods for disease classification
Source: Gigascience. 2019 Apr 26;8(5):giz045. doi: 10.1093/gigascience/giz045 (PMC6486474; doi:10.1093/gigascience/giz045)
Supplement: Reviewer_2_Report_Original_Submission -- Shefali Setia Verma — 11/11/2018 Reviewed [file giz045_reviewer_2_report_original_submission.pdf]

Reviewer Report

Title: A multi-omics data simulator for complex disease studies and its application to evaluate multi-omics data analysis methods for disease classification

Version: Original Submission **Date:** 11/11/2018

Reviewer name: Shefali Setia Verma

Reviewer Comments to Author:

1) This manuscript presents a very interesting expansion to existing simulation tools by adding various 'omics simulations possible. This is definitely a requirement in the field, though I want to mention that there are other tools like HIBACHI (https://www.ncbi.nlm.nih.gov/pmc/articles/PMC4270828/) which also aim to achieve similar integrated framework to mimic the biology and interactions among various omic datasets to simulate the data. Authors must revise the sentences to mention how OmicSIMLA is different and also is not the first comics simulator.
OmicsSIMLA Tool Review for GigaScience
*just want to preface that some of these points may be more intuitive to others, but from my perspective these were some of the challenges
2) Authors do a nice job of breaking down what the required input files are for simulating each specific data type (genotype, CNV, WGBSS, gene expression, and protein expression);
however, the role of "Profiles" is a bit unclear. For example, gene expression "Profile" data contains gene expression beta values, but whether a user is to input their own beta values or if there is a standard is unclear. Additionally, the gene expression profile data contains individual-specific normalization factors (mean and dispersion parameters) to create the negative binomial distribution. The file contains values for 103 normal tissue but there is no information on how to actually use or interpret this data. Please add more clarity on the same.
3) There is methylation profile data which is specific to 29 cell types and tissues. This is quite easy to maneuver when generating methylation simulations. There is a protein expression profile which contains standard deviations for the normal distribution. The parameters are based on 874 breast cancer patients from the TCPA. If the gene expression and protein expression profiles were also based on tissue type (maybe something like GTEx), that would be really useful.
4) To summarize above two points, more details about the role and application of "profile" data would be helpful and why the data is skewed for certain distributions. Do users need to use the same profiles to generate simulations, and if not, does their data need to fit a certain distribution?
5) For the input file in SeqSIMLA, a variety of reference files can be input to bolster specificity of the simulated genotypes, i.e. refseq, penetrance, pedigree structure, etc. Most of these are in PLINK file format so that is good. However, what is a bit unclear is how much of this information is required to generate simulated data. For example, in HIBACHI, you could specify the bare minimum parameters to generate a simulation, of course with the drawback being that the data had no biological context. Here it is evident the data has biological context, but there should be more information for use case; how much/which type of data is required to generate the genotype simulation? It is not very clear what is

"required" and what is "optional".
6) It appears that this tool is best used if you have datasets already but are missing a specific desired dataset. For example, if you have genotype and methylation, and you know the data well enough to expect values for missing protein expression data type. Or if you have very specific requirements for the type of simulated data you want. This tool doesn't seem like the best option if you are simulating all datatypes from scratch, authors should clarify if this is the case because this point is not mentioned in the manuscript. And if this is not the case, more clarification on how all data types can be generated from scratch would be very useful.
7) Another example of the last comment is that for the tutorial, they provide the following 3 files: 1) a reference .bim file containing 2022 SNPs, 2) a .txt file specifying site interaction effects between SNPs using an odds ratio, and 3) a .txt file containing information on CNVs between sites and the frequency of deletions/odds ratio of deletion. Therefore, it seems to me you are required to have a good amount of input data in order to generate the simulations.
8) For simulating gene expression data, it seems that beta values are required. It would be beneficial to mention where to obtain beta values, should these always be constant from public resources like GTex?
9) I used the tutorials to generate simulations: for the most part they were as expected. However, when simulating multi-omics data, I was unable to generate a .cnv file as is outlined in the tutorial under "Several files will be generated:".
10) Additionally, upon generating successful simulations, I received the following error every time:
sh: ../bin/bug_reporter: cannot execute binary file
However, I have not found any documentation on bug_reporter after compilation. I am not sure exactly what is meant by the error.

Methods

Are the methods appropriate to the aims of the study, are they well described, and are necessary controls included? Choose an item.

Conclusions

Are the conclusions adequately supported by the data shown? Choose an item.

Reporting Standards

Does the manuscript adhere to the journal's guidelines on [minimum standards of reporting?]() Choose an item.

Choose an item.

Statistics

Are you able to assess all statistics in the manuscript, including the appropriateness of statistical tests used? Choose an item.

Quality of Written English

Please indicate the quality of language in the manuscript: Choose an item.

Declaration of Competing Interests

Please complete a declaration of competing interests, considering the following questions:

- Have you in the past five years received reimbursements, fees, funding, or salary from an organisation that may in any way gain or lose financially from the publication of this manuscript, either now or in the future?
- Do you hold any stocks or shares in an organisation that may in any way gain or lose financially from the publication of this manuscript, either now or in the future?
- Do you hold or are you currently applying for any patents relating to the content of the manuscript?
- Have you received reimbursements, fees, funding, or salary from an organization that holds or has applied for patents relating to the content of the manuscript?
- Do you have any other financial competing interests?
- Do you have any non-financial competing interests in relation to this paper?

If you can answer no to all of the above, write 'I declare that I have no competing interests' below. If your reply is yes to any, please give details below.

I declare that I have no competing interests

I agree to the open peer review policy of the journal. I understand that my name will be included on my report to the authors and, if the manuscript is accepted for publication, my named report including any attachments I upload will be posted on the website along with the authors' responses. I agree for my report to be made available under an Open Access Creative Commons CC-BY license (http://creativecommons.org/licenses/by/4.0/). I understand that any comments which I do not wish to be included in my named report can be included as confidential comments to the editors, which will not be published.

I agree to the open peer review policy of the journal

To further support our reviewers, we have joined with Publons, where you can gain additional credit to further highlight your hard work (see: https://publons.com/journal/530/gigascience). On publication of this paper, your review will be automatically added to Publons, you can then choose whether or not to claim your Publons credit. I understand this statement.

Yes Choose an item.
